# The effects of rhythm control strategies versus rate control strategies for atrial fibrillation and atrial flutter: A systematic review with meta-analysis and Trial Sequential Analysis

**DOI:** 10.1371/journal.pone.0186856

**Published:** 2017-10-26

**Authors:** Naqash J. Sethi, Joshua Feinberg, Emil E. Nielsen, Sanam Safi, Christian Gluud, Janus C. Jakobsen

**Affiliations:** 1 Copenhagen Trial Unit, Centre for Clinical Intervention Research, Department, Rigshospitalet, Copenhagen University Hospital, Copenhagen, Denmark; 2 The Cochrane Hepato-Biliary Group, Copenhagen Trial Unit, Centre for Clinical Intervention Research, Department, Rigshospitalet, Copenhagen University Hospital, Copenhagen, Denmark; 3 Department of Cardiology, Holbæk Hospital, Holbæk, Denmark; Universita degli Studi di Perugia, ITALY

## Abstract

**Background:**

Atrial fibrillation and atrial flutter may be managed by either a rhythm control strategy or a rate control strategy but the evidence on the clinical effects of these two intervention strategies is unclear. Our objective was to assess the beneficial and harmful effects of rhythm control strategies versus rate control strategies for atrial fibrillation and atrial flutter.

**Methods:**

We searched CENTRAL, MEDLINE, Embase, LILACS, Web of Science, BIOSIS, Google Scholar, clinicaltrials.gov, TRIP, EU-CTR, Chi-CTR, and ICTRP for eligible trials comparing any rhythm control strategy with any rate control strategy in patients with atrial fibrillation or atrial flutter published before November 2016. Our primary outcomes were all-cause mortality, serious adverse events, and quality of life. Our secondary outcomes were stroke and ejection fraction. We performed both random-effects and fixed-effect meta-analysis and chose the most conservative result as our primary result. We used Trial Sequential Analysis (TSA) to control for random errors. Statistical heterogeneity was assessed by visual inspection of forest plots and by calculating inconsistency (I^2^) for traditional meta-analyses and diversity (D^2^) for TSA. Sensitivity analyses and subgroup analyses were conducted to explore the reasons for substantial statistical heterogeneity. We assessed the risk of publication bias in meta-analyses consisting of 10 trials or more with tests for funnel plot asymmetry. We used GRADE to assess the quality of the body of evidence.

**Results:**

25 randomized clinical trials (n = 9354 participants) were included, all of which were at high risk of bias. Meta-analysis showed that rhythm control strategies versus rate control strategies significantly increased the risk of a serious adverse event (risk ratio (RR), 1.10; 95% confidence interval (CI), 1.02 to 1.18; P = 0.02; I^2^ = 12% (95% CI 0.00 to 0.32); 21 trials), but TSA did not confirm this result (TSA-adjusted CI 0.99 to 1.22). The increased risk of a serious adverse event did not seem to be caused by any single component of the composite outcome. Meta-analysis showed that rhythm control strategies versus rate control strategies were associated with better SF-36 physical component score (mean difference (MD), 6.93 points; 95% CI, 2.25 to 11.61; P = 0.004; I^2^ = 95% (95% CI 0.94 to 0.96); 8 trials) and ejection fraction (MD, 4.20%; 95% CI, 0.54 to 7.87; P = 0.02; I^2^ = 79% (95% CI 0.69 to 0.85); 7 trials), but TSA did not confirm these results. Both meta-analysis and TSA showed no significant differences on all-cause mortality, SF-36 mental component score, Minnesota Living with Heart Failure Questionnaire, and stroke.

**Conclusions:**

Rhythm control strategies compared with rate control strategies seem to significantly increase the risk of a serious adverse event in patients with atrial fibrillation. Based on current evidence, it seems that most patients with atrial fibrillation should be treated with a rate control strategy unless there are specific reasons (e.g., patients with unbearable symptoms due to atrial fibrillation or patients who are hemodynamically unstable due to atrial fibrillation) justifying a rhythm control strategy. More randomized trials at low risk of bias and low risk of random errors are needed.

**Trial registration:**

PROSPERO CRD42016051433

## Introduction

Atrial fibrillation is the most common arrhythmia of the heart with a prevalence of approximately 2% in the western world [1, 2]. Atrial fibrillation and atrial flutter are both associated with an increased risk of morbidity and death [3–9]. The risks of both cerebral stroke and heart failure are increased nearly fivefold in patients with atrial fibrillation and atrial flutter and about 20% of every stroke may be due to atrial fibrillation [3–8]. Atrial fibrillation and atrial flutter also have a significant impact on healthcare costs and account for approximately 1% of the National Health Service budget in the United Kingdom and approximately 26 billion dollars of annual expenses in the United States [10, 11].

Two different overall intervention strategies may be used for atrial fibrillation and atrial flutter–a rhythm control strategy and a rate control strategy [12, 13]. When using any rhythm control strategy, the aim is to obtain and maintain sinus rhythm, while the overall aim when using any rate control strategy is to lower the ventricular frequency [14].

No former systematic review assessing the effects of rhythm control strategies versus rate control strategies for atrial fibrillation or atrial flutter has searched all relevant databases and has considered both risks of systematic errors and risks of random errors [15–18].

## Methods

We conducted this systematic review based on the Preferred Reporting Items for Systematic Reviews and Meta-Analysis guidelines (PRISMA) (S1 Text) [19, 20], and the updated Cochrane methodology used in this systematic review is described in detail in our protocol (S2 Text), which was registered prior to the systematic literature search [18, 21, 22].

### Search strategy and selection criteria

We searched for trials comparing any rhythm control strategy with any rate control strategy in patients with atrial fibrillation or atrial flutter. We searched for eligible trials published before November 2016 in the Cochrane Central Register of Controlled Trials (CENTRAL), MEDLINE, Embase, LILACS, Science Citation Index Expanded on Web of Science, BIOSIS, Google Scholar, clinicaltrials.gov, Trip Medical Database (TRIP), EU Clinical Trial Register (EU-CTR), Chinese Clinical Trial Registry (ChiCTR), and WHO International Clinical Trials Registry Platform (ICTRP) [21]. The search strategy can be found in the supplementary material (S3 Text). Additionally, we checked the reference lists of relevant publications for any unidentified trials. Trials were included irrespective of trial design, setting, publication status, publication year, language, and the reporting of one of our outcomes.

### Data extraction and risk of bias assessment

Three authors (NJS, JF, EEN) independently selected relevant trials, and four authors (NJS, SS, JF, EEN) extracted data using a standardized data extraction sheet and assessed the risk of bias according to the Cochrane Handbook for Systematic Reviews of Interventions and Lundh et al. [18, 23]. Any discrepancies were discussed with a fifth review author (JCJ). We attempted to contact trial authors if relevant data were unclear or missing.

#### Outcomes and subgroup analysis

Our primary outcomes were all-cause mortality, serious adverse events (as defined by the ICH guidelines) [24], and quality of life. Our secondary outcomes were stroke and ejection fraction. All outcomes were analyzed as proportions of participants in each intervention group except for quality of life and ejection fraction which were both analyzed as continuous outcomes. For all outcomes, we used the trial results reported at maximal follow-up. However, if the trialists reported results at multiple time-points, we used the results reported at the time-point closest to 24 months.

We planned the following subgroup analyses on our primary outcomes:

- comparison of different types of rhythm control interventions;
- comparison of different types of rate control interventions;
- comparison of different mean ages of participants;
- comparison of different durations of atrial fibrillation;
- comparison of different durations of anticoagulation therapy;
- comparison of participants with atrial fibrillation to participants with atrial flutter; and
- comparison of trials only randomizing men to trials only randomizing women.

Additionally, we performed a post-hoc subgroup analysis:

- comparison of participants with heart failure to participants without heart failure.

### Assessment of statistical and clinical significance

We performed our meta-analyses according to the recommendations stated in the *Cochrane Handbook for Systematic Reviews of Interventions* [18], Keus et al. [17], and the eight-step assessment suggested by Jakobsen et al. [15] for better validation of meta-analytic results in systematic reviews. Review Manager 5 and Stata 15 were used for all meta-analyses [25, 26]. We used risk ratios (RR) for dichotomous outcomes and mean differences (MD) for continuous outcomes. We did not use standardized mean difference (SMD) when analyzing continuous outcomes due to the fact that the outcomes assessed were not homogeneous and the several methodological limitations of using this approach [18]. We performed both random-effects (DerSimonian-Laird model) and fixed-effect meta-analysis with the Mantel-Haenszel method and chose the most conservative result as our primary result [15]. The more conservative result was the result with the highest P value and the widest 95% confidence interval (CI). If there was substantial discrepancy between the results of the two methods, we reported and discussed the results [15]. We used Trial Sequential Analysis (TSA) to control for random errors and reported TSA-adjusted CI if the cumulative Z-curves did not reach the futility area or passed the diversity-adjusted required information size (DARIS) [15, 16, 21, 22, 27–35]. TSA estimates the DARIS (that is the number of participants needed in a meta-analysis to detect or reject a certain intervention effect). When analyzing dichotomous outcomes, we pragmatically anticipated an intervention effect of 15% risk ratio reduction (RRR). When analyzing continuous outcomes, we pragmatically anticipated an intervention effect equal to the MD of the observed SD/2 [36]. Statistical heterogeneity was assessed by visual inspection of forest plots and by calculating inconsistency (I^2^) for traditional meta-analyses and diversity (D^2^) for TSA [31]. We calculated the 95% CI of the inconsistency (I^2^) with the TSA software [27]. Sensitivity analyses and subgroup analyses were conducted to explore the reasons for substantial statistical heterogeneity [15]. We assessed the risk of publication bias in meta-analyses consisting of 10 trials or more with tests for funnel plot asymmetry. We assessed three primary outcomes and, hence, considered a P value of 0.025 or less as the threshold for statistical significance for the primary outcomes [15, 37]. We assessed two secondary outcomes and, hence, considered a P value of 0.033 as the threshold for statistical significance for the secondary outcomes [15, 37]. We used ‘best-worst case’ analyses and ‘worst-best case’ analyses to assess the potential impact of missing data (incomplete outcome data bias) [15]. We calculated Bayes factor to show if the meta-analysis results fitted better with the null hypotheses or the anticipated intervention effects [15]. We used GRADE to assess the quality of the body of evidence [15, 38–40].

## Results

### Study characteristics

**Fig 1:**
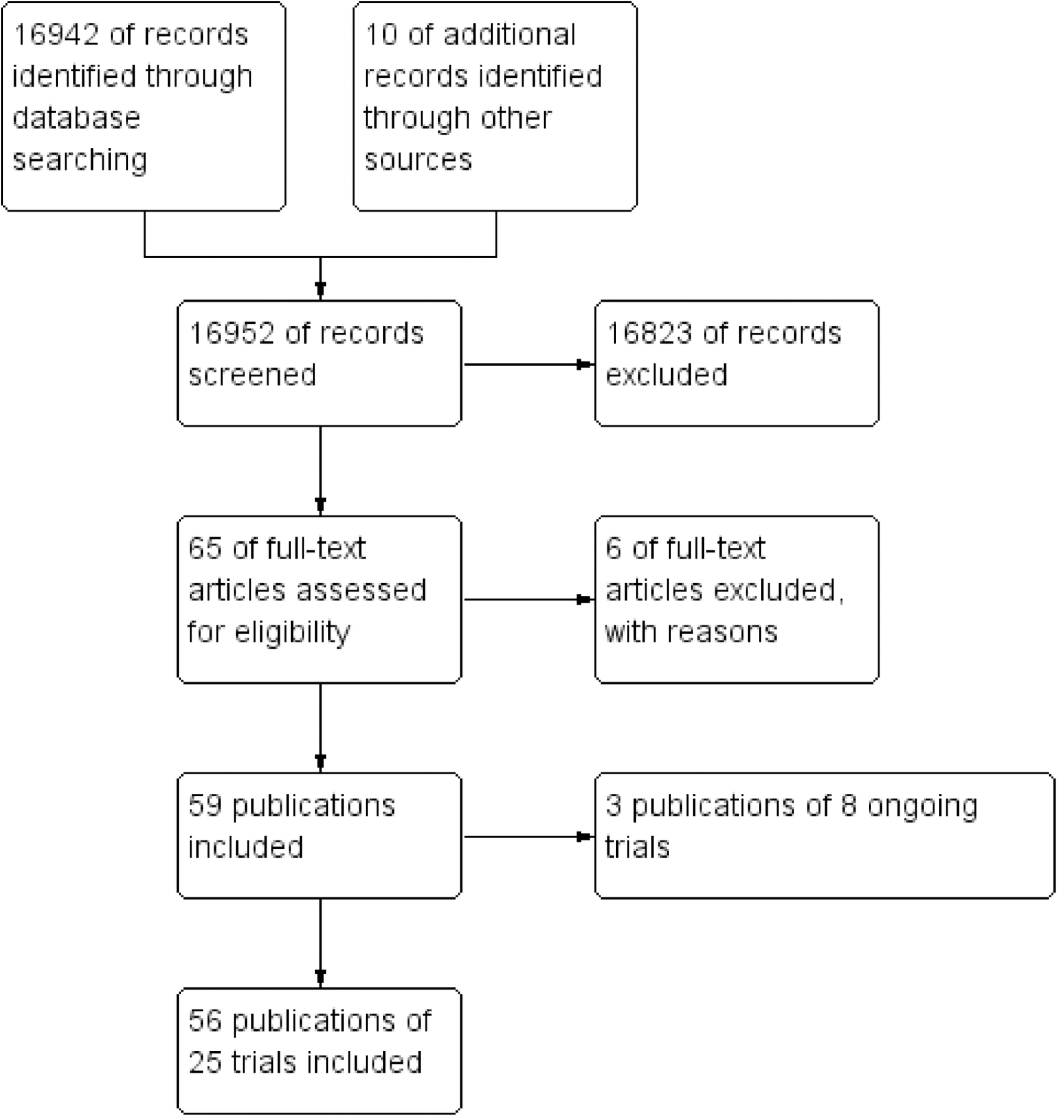
PRISMA flow diagram. We screened 16 952 records and included 56 publications of 25 trials in this systematic review.

Our literature search identified a total of 16 952 papers. We included 25 randomized clinical trials with 26 trial comparisons including a total of 9354 participants (Fig 1) [41–96]. All trials were at high risk of bias (S1 Table). All trials included participants with atrial fibrillation and three trials included both participants with atrial fibrillation and flutter [50, 62, 86]. The individual trials used various types of rhythm control interventions and rate control interventions. The rhythm control interventions used were: amiodarone with or without electrical cardioversion (6/26 trial comparisons); not specified (6/26 trial comparisons); electrical cardioversion with antiarrhythmic drug therapy following sinus rhythm restoration (5/26 trial comparisons); catheter ablation (4/26 trial comparisons); antiarrhythmic therapy with or without electrical cardioversion (2/26 trial comparisons); ibutilide (1/26 trial comparisons); propafenone (1/26 trial comparisons); and total endoscopic ablation (1/26 trial comparisons). The rate control interventions used were: beta-blockers, calcium channel blockers, digoxin, or a combination of these (11/26 trial comparisons); not specified (5/26 trial comparisons); AV-node ablation (4/26 trial comparisons); digoxin and/or beta blockers (2/26 trial comparisons); diltiazem (2/26 trial comparisons); beta blockers, calcium channel blockers, digoxin, and/or AV-node ablation (1/26 trial comparisons); and digoxin, carvedilol, and/or bisoprolol (1/26 trial comparisons). We have summarized the inclusion- and exclusion criteria for each included trial in S2 Table and other trial characteristics in S3 Table. Additionally, we have summarized the characteristics of excluded studies [97–102] and characteristics of ongoing trials [103–105] in S3 Table.

### All-cause mortality

**Fig 2:**
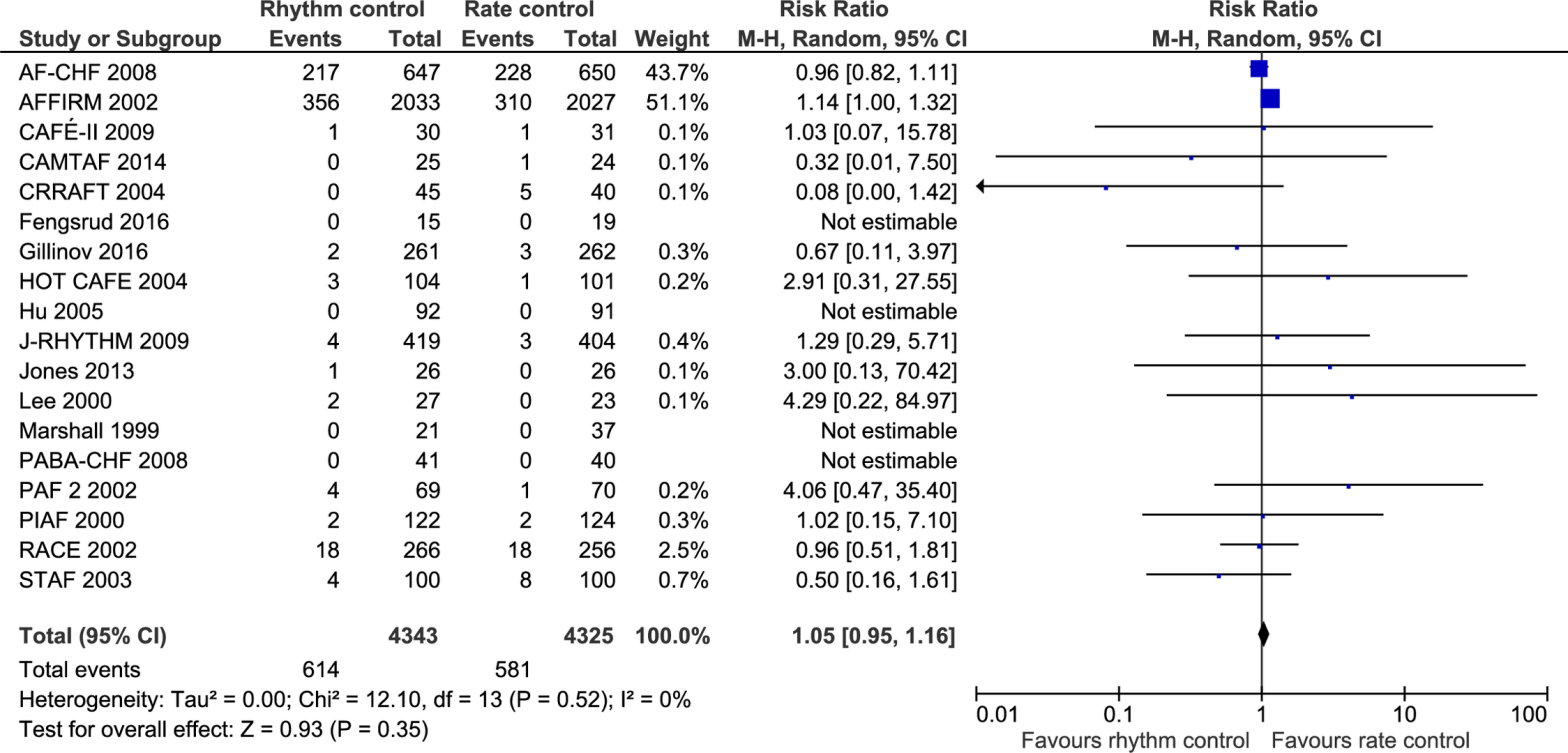
Forest plot of the meta-analysis of all-cause mortality. Meta-analysis showed no significant difference between rhythm control strategies and rate control strategies when assessing all-cause mortality.

**Fig 3:**
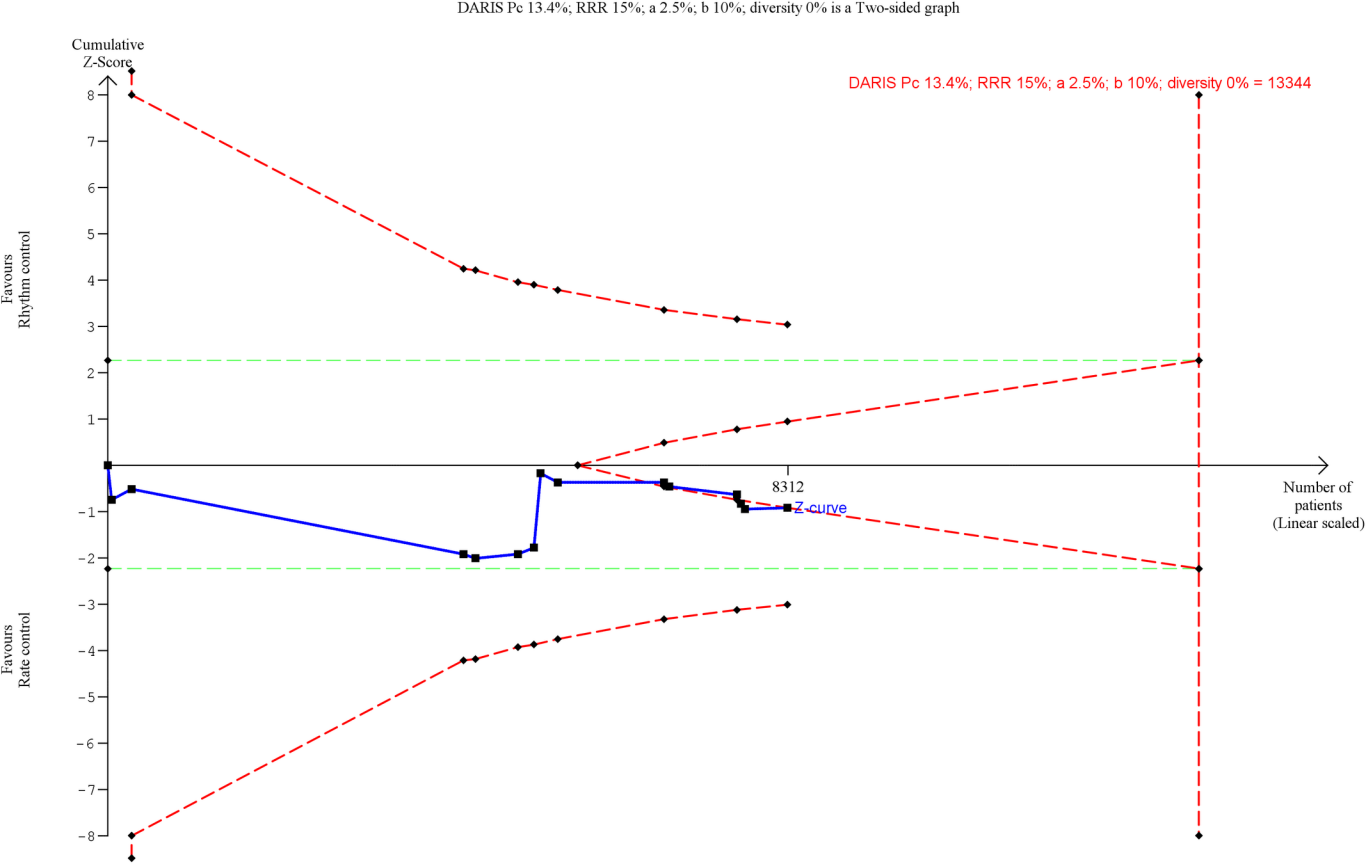
Trial Sequential Analysis of all-cause mortality. Trial Sequential Analysis (TSA) showed that there was not enough information to confirm or reject a risk ratio reduction of 15% (TSA-adjusted confidence interval 0.90 to 1.22). The Z-curve (the blue line) does not cross any boundaries.

18 trials randomizing a total of 8668 participants reported all-cause mortality. Meta-analysis showed no significant difference between rhythm control strategies and rate control strategies (RR, 1.05; 95% CI, 0.95 to 1.16; P = .35; I^2^ = 0% (95% CI 0 to 33%); Bayes factor = 3438; Fig 2). Visual inspection of the forest plot showed no signs of heterogeneity (Fig 2). The TSA showed that there was not enough information to confirm or reject a RRR of 15% (TSA-adjusted CI 0.90 to 1.22) (Fig 3). Incomplete outcome data bias alone had the potential to influence the results in the ‘worst-best case’-scenario (S1 and S2 Figs). Visual inspection of the funnel plot showed no clear signs of asymmetry (S3 Fig).

### Serious adverse events

**Fig 4:**
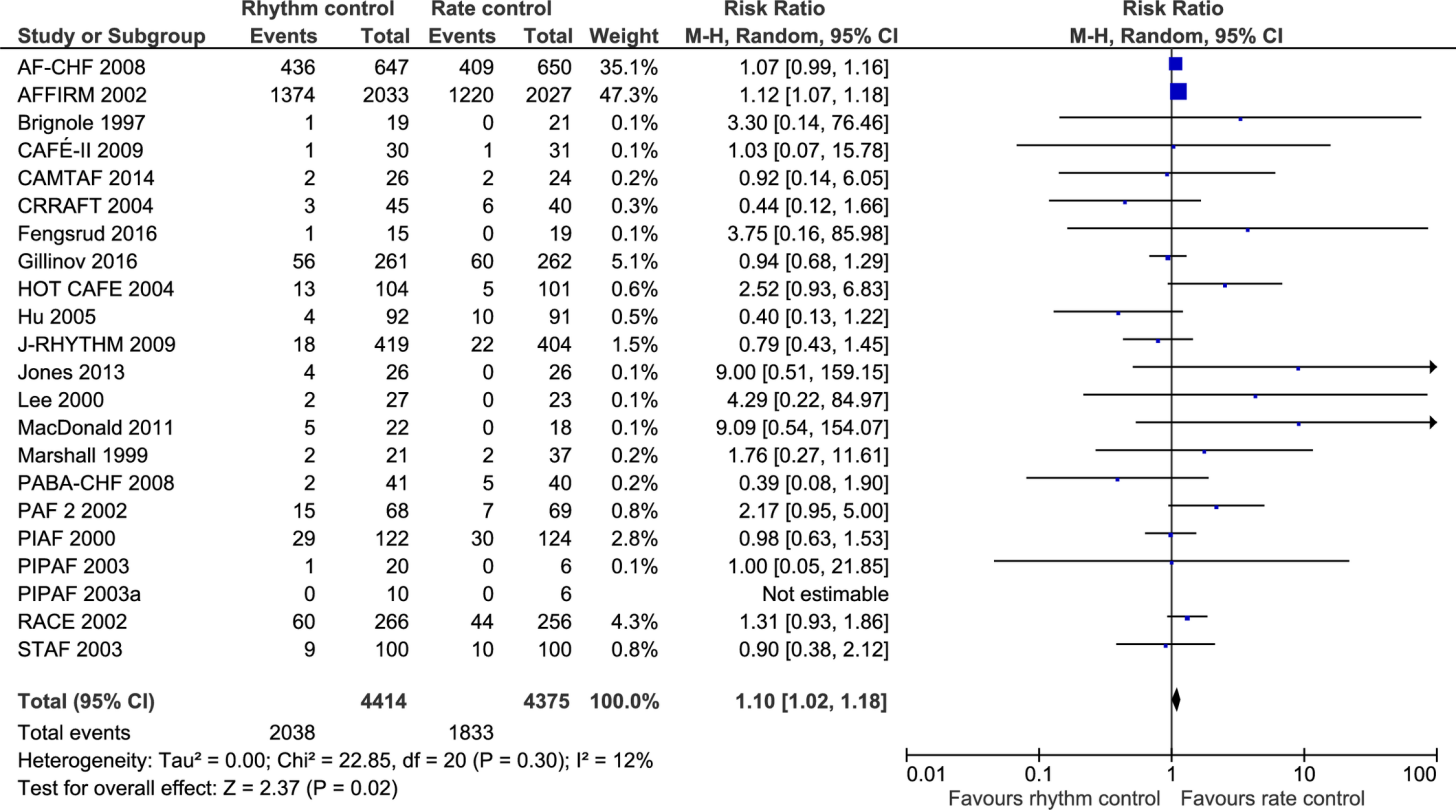
Forest plot of the meta-analysis of serious adverse events. Meta-analysis showed that rhythm control strategies versus rate control strategies significantly increased the risk of a serious adverse event.

**Fig 5:**
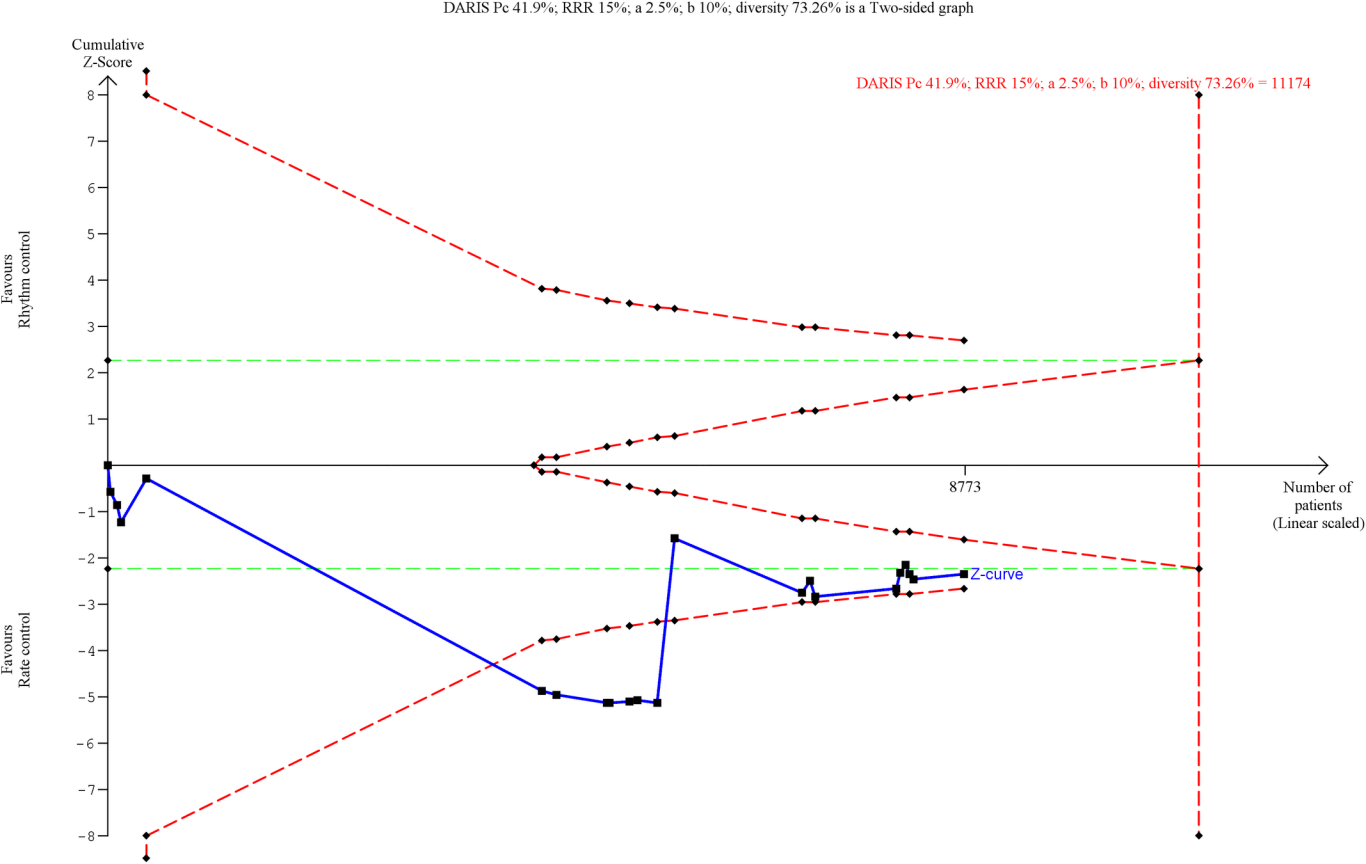
Trial Sequential Analysis of serious adverse events. Trial Sequential Analysis (TSA) of serious adverse events showed that there was not enough information to confirm or reject a RRR of 15% (TSA-adjusted confidence interval 0.99 to 1.22). The Z-curve (the blue line) does not cross any boundaries.

21 trials randomizing a total of 8789 participants reported the proportion of participants with a serious adverse event. Meta-analysis showed that rhythm control strategies versus rate control strategies significantly increased the risk of a serious adverse event (RR, 1.10; 95% CI, 1.02 to 1.18; P = 0.02; I^2^ = 12% (95% CI 0 to 32%); Bayes factor = 1.05^e9^; Fig 4). Visual inspection of the forest plot did not show signs of heterogeneity (Fig 4). The TSA showed that there was not enough information to confirm or reject a RRR of 15% (TSA-adjusted CI 0.99 to 1.22) (Fig 5). Incomplete outcome data bias alone had the potential to influence the results in the ‘best-worst case’-scenario (S4 and S5 Figs). Visual inspection of the funnel plot showed a bit asymmetry (S6 Fig), but Harbord’s test showed no significance (P = 0.63).

We have summarized the specific types of serious adverse events in each trial in S4 Table.

We did not include hospitalization for non-acute electrical cardioversion or hospitalization for elective antiarrhythmic drug loading as a serious adverse event, as readmission for non-acute electrical cardioversion and readmission for elective antiarrhythmic drug loading in most trials was mandated by the individual trial protocols (see ‘Discussion‘).

The four primary components of the composite outcome serious adverse event (excluding all-cause mortality and stroke) were myocardial infarction, heart failure, ventricular tachycardia, and hospitalization (excluding hospitalization for non-acute electrical cardioversion and hospitalization for elective antiarrhythmic drug loading). Meta-analysis of either myocardial infarction, heart failure, ventricular tachycardia, or hospitalization (excluding hospitalization for non-acute electrical cardioversion and hospitalization for elective antiarrhythmic drug loading) showed no significant difference between rhythm control strategies and rate control strategies (S7–S10 Figs).

### Subgroup analysis for all-cause mortality and serious adverse events

All the planned tests for subgroup differences when analyzing both all-cause mortality (S11–S15 Figs) and serious adverse events (S16–S20 Figs) showed no significant differences. Two of the planned subgroup analyses (comparison of participants with atrial fibrillation to those with atrial flutter; and comparison of trials only randomizing men to trials only randomizing women) were not possible to conduct due to lack of relevant data. The post hoc subgroup analysis (comparison of participants with heart failure to participants without heart failure) also showed no significant differences when analyzing all-cause mortality (S21 Fig) and serious adverse events (S22 Fig).

### Quality of life

Quality of life was only assessed in 13 out of 24 trials and different assessment scales were used, including SF-36, Minnesota Living with Heart Failure Questionnaire, Kansas City Cardiomyopathy Questionnaire, Psychological General Well-Being Index, and Mental Health Inventory. All trials reported standard deviations for their analyses. Hence, we did not need to impute standard deviations.

**Fig 6:**
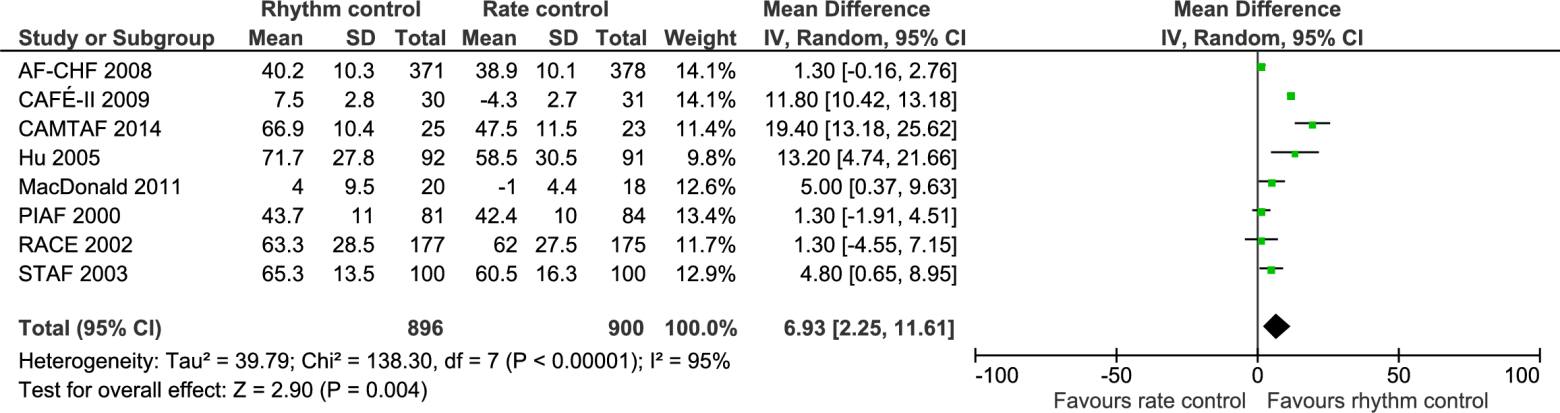
Forest plot of the meta-analysis of quality of life (the Short Form (36) physical component score (SF-36 PCS)). Meta-analysis showed that rhythm control strategies versus rate control strategies significantly increased the quality of life measured by SF-36 PCS.

**Fig 7:**
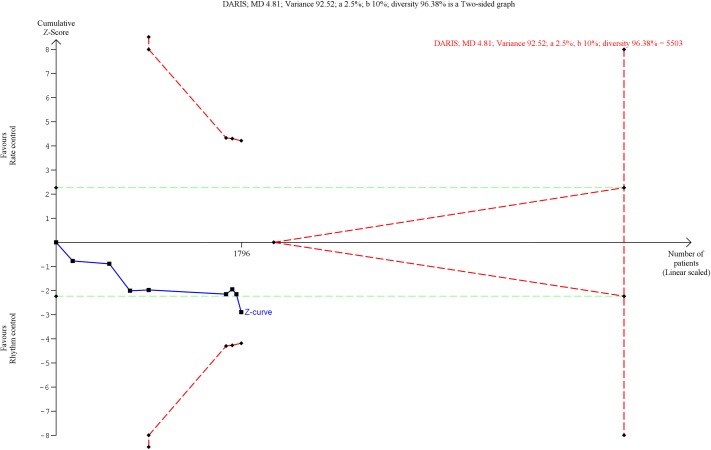
Trial Sequential Analysis of quality of life (the Short Form (36) physical component score (SF-36 PCS)). Trial Sequential Analysis showed that there was not enough information to confirm or reject a mean difference of 4.81 points (TSA-adjusted confidence interval -3.16 to 17.02). The Z-curve (the blue line) does not cross any boundaries.

Only data from SF-36 physical component score, SF-36 mental component score, and Minnesota Living with Heart Failure Questionnaire could be used in meta-analyses. The only meta-analysis showing a statistically significant result was the analysis of the results of SF-36 physical component score (MD, 6.93 points in favor of the rhythm control group; 95% CI, 2.25 to 11.61; P = 0.004; I^2^ = 95% (95% CI 94 to 96%); Bayes factor = 0.022; Fig 6). However, both visual inspection of the forest plot (Fig 6) and the statistical tests showed considerable heterogeneity (I^2^ = 95% (95% CI 94 to 96%)), and the TSA showed that there was not enough information to confirm or reject a MD of 4.81 points (TSA-adjusted CI -3.16 to 17.02) (Fig 7). Furthermore, incomplete outcome data bias alone had the potential to influence the results (S23 and S24 Figs). The remaining meta-analyses (analysis of SF-36 mental component score and Minnesota Living with Heart Failure Questionnaire) showed no significant differences (S25 and S26 Figs), and the TSAs showed that there was not enough information to confirm or reject our anticipated intervention effects (S27 Fig). We have summarized all these results in Table 1.

**Table 1:** Quality of life, results for each type of scale.

	Trials	Participants	Mean difference (points)	95% confidence interval (CI)	Trial Sequential Analysis—adjusted CI	P-value	I^2^ [95% CI]	Bayes factor	Best-worst case scenario (MD [95% CI])	Worst-best case scenario (MD [95% CI])
**SF-36 mental component score**	8	1796	3.33	-0.75 to 7.41	-4.47 to 11.13	0.11	93% [92 to 95%]	0.35	8.16 [5.45 to 10.87]	-1.25 [-8.55 to 6.04]
**Minnesota Living With Heart Failure Questionnaire**	6	404	-7.13	-16.19 to 1.94	-	0.12	95% [93 to 96%]	3.73	-8.51 [-17.84 to 0.82]	-5.41 [-14.55 to 3.73]
**Kansas City Cardiomyopathy Questionnaire**	1	38	1.50	-9.78 to 12.78	-	0.79	-	-	-	-
**Psychological General Well-Being Index**	1	56	-8.9	-18.16 to 0.36	-	0.06	-	-	-	-
**Mental Health Inventory**	1	56	-0.4	-2.1 to 1.3	-	0.64	-	-	-	-

### Stroke

**Fig 8:**
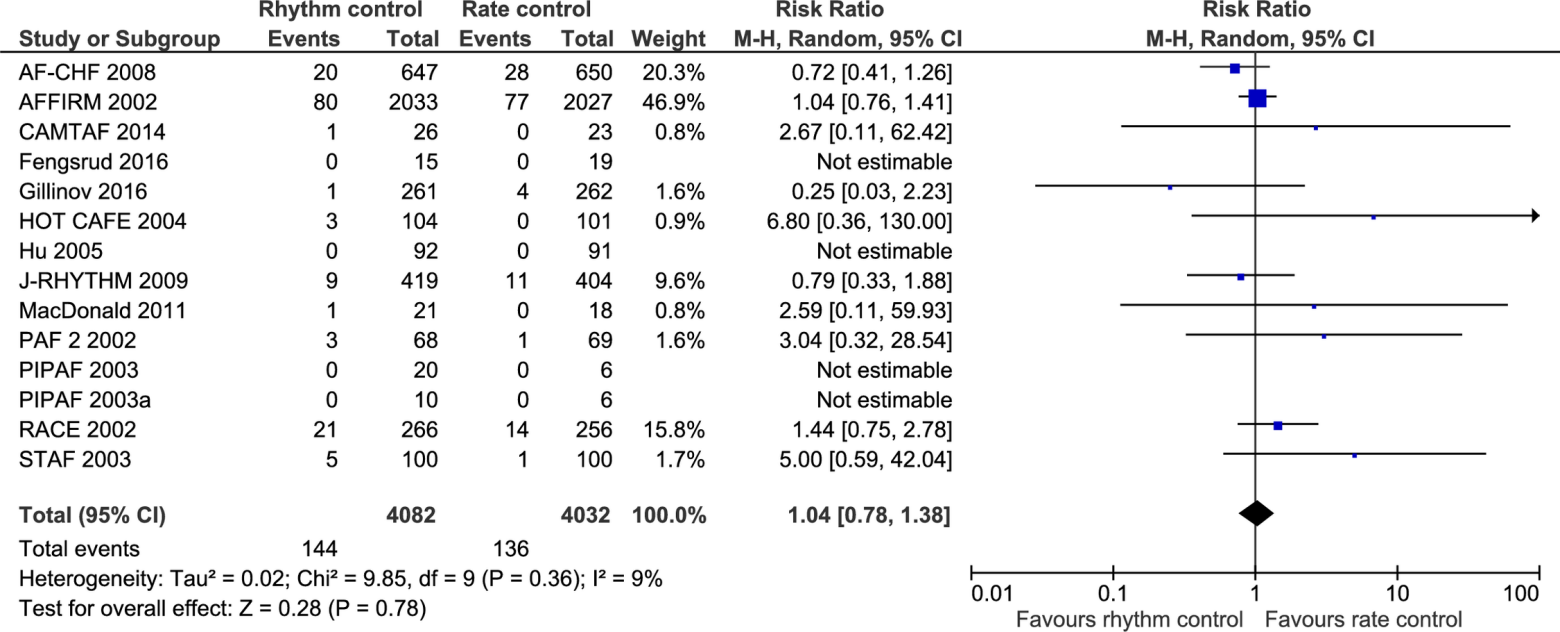
Forest plot of the meta-analysis of stroke. Meta-analysis showed no significant difference between rhythm control strategies and rate control strategies when assessing stroke.

**Fig 9:**
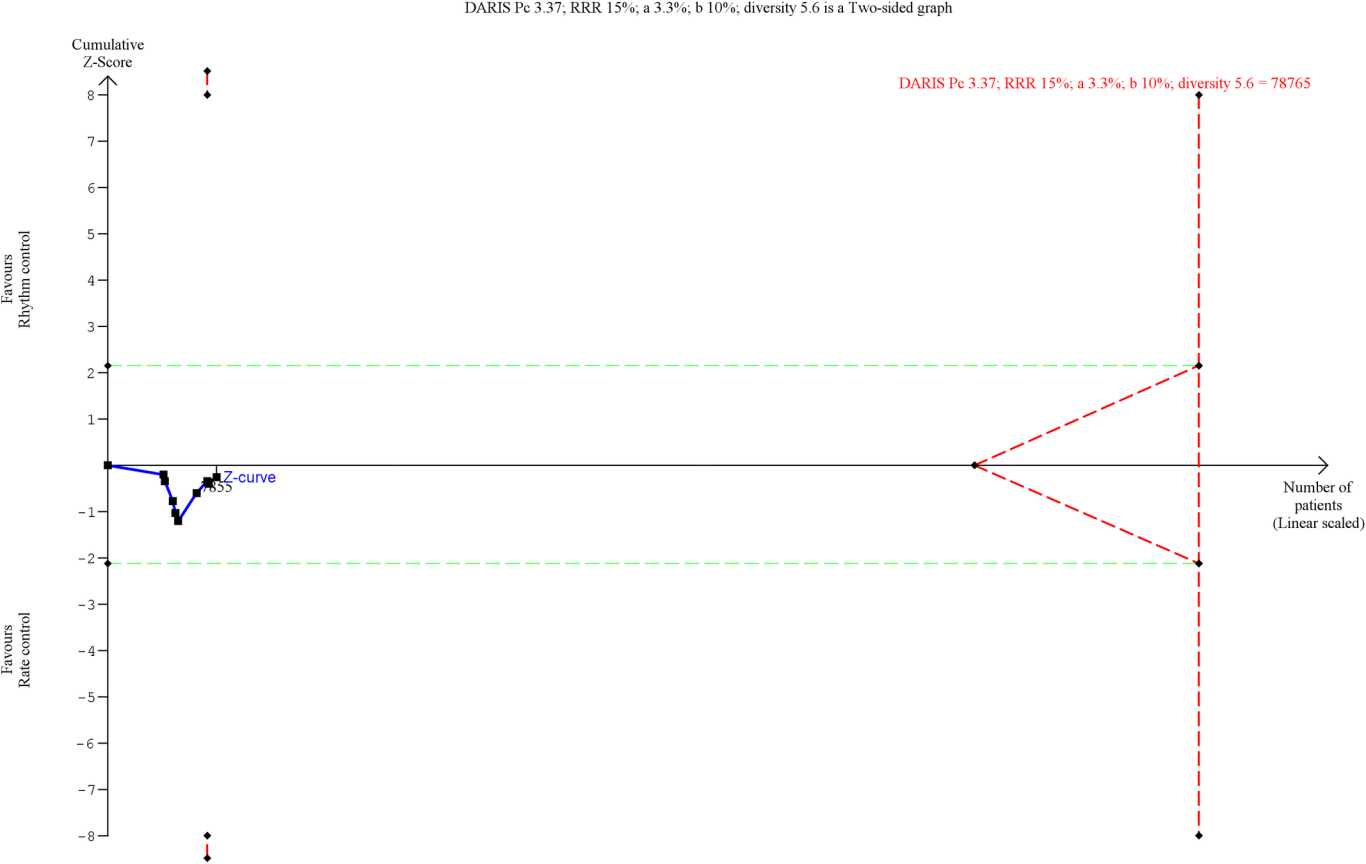
Trial Sequential Analysis of stroke. Trial Sequential Analysis (TSA) showed that there was not enough information to confirm or reject a risk ratio reduction of 15% (TSA-adjusted confidence interval 0.33 to 3.28). The Z-curve (the blue line) does not cross any boundaries.

13 trials randomizing a total of 8114 participants reported the proportion of participants with stroke. Meta-analysis showed no significant difference between rhythm control strategies versus rate control strategies (RR, 1.04; 95% CI, 0.78 to 1.38; P = .78; I^2^ = 9% (95% CI 0 to 44%); Fig 8). Visual inspection of the forest plot did not show signs of heterogeneity (Fig 8). The TSA showed that there was not enough information to confirm or reject a RRR of 15% (TSA-adjusted CI 0.33 to 3.28) (Fig 9). Incomplete outcome data bias alone did not have the potential to influence the results (S28 and S29 Figs).

### Ejection fraction

**Fig 10:**
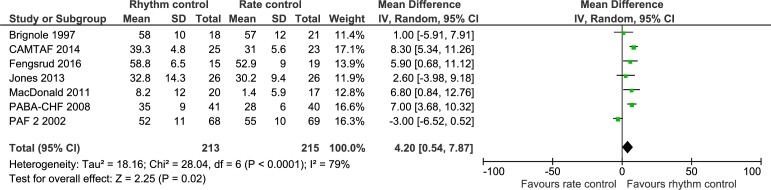
Forest plot of the meta-analysis of ejection fraction. Meta-analysis showed that rhythm control strategies versus rate control strategies significantly increased the ejection fraction.

**Fig 11:**
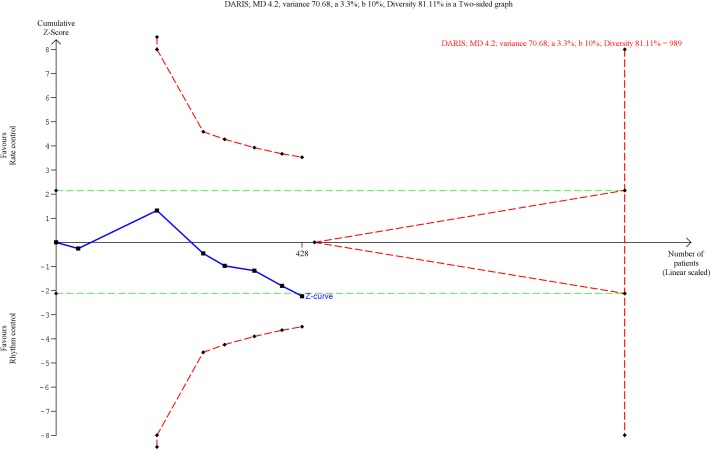
Trial Sequential Analysis of ejection fraction. Trial Sequential Analysis (TSA) showed that there was not enough information to confirm or reject a mean difference of 4.20% (TSA-adjusted confidence interval -2.37 to 10.77). The Z-curve (the blue line) does not cross any boundaries.

Seven trials randomizing a total of 428 participants assessed the effects of rhythm control strategies versus rate control strategies on ejection fraction. Meta-analysis showed that rhythm control strategies versus rate control strategies significantly increased the mean ejection fraction (MD, 4.20%; 95% CI, 0.54 to 7.87; P = 0.02; I^2^ = 79% (95% CI 69 to 85%); Fig 10). Visual inspection of the forest plot showed some signs of heterogeneity (Fig 10). The TSA showed that there was not enough information to confirm or reject a MD of 4.20% (TSA-adjusted CI -2.37 to 10.77) (Fig 11). Incomplete outcome data bias alone had the potential to influence the results in the ‘worst-best case’ scenario (S30 and S31 Figs).

We have summarized our main results in the Summary of Findings table (Table 2).

**Table 2:** Summary of Findings table.

Summary of Findings table						
Outcomes	Anticipated absolute effects		Relative effect (Trial Sequential Analysis-adjusted confidence interval)	№ of participants (trials)	Quality of the evidence (GRADE)	Comments
	Risk with rhythm control strategy	Risk with rate control strategy				
All-cause mortality	141 per 1000	134 per 1000	1.05 (0.90 to 1.122)	8668 (18 trials)	⊕⊝⊝⊝ - Very low quality of evidence caused by risk of bias (-2) and imprecision (-1).	Trial Sequential Analysis showed that there was not enough information to confirm or reject a RRR of 15% or more. All trials had high risk of bias, mostly because of ‘blinding of participants and personnel’, ‘incomplete outcome data bias’, and ‘for-profit bias’.
Serious adverse events	462 per 1000	419 per 1000	1.10 (0.99 to 1.22)	8789 (21 trials)	⊕⊝⊝⊝ - Very low quality of evidence caused by risk of bias (-2) and imprecision (-1).	Trial Sequential Analysis showed that there was not enough information to confirm or reject a RRR of 15% or more. All trials had high risk of bias, mostly because of ‘blinding of participants and personnel’, ‘incomplete outcome data bias’, and ‘for-profit bias’.
Quality of life	Quality of life showed a significant effect of rhythm control versus rate control on the SF-36 physical component score (MD 6.93, Trial Sequential Analysis-adjusted confidence interval -3.16 to 17.02).			1796 (8 trials) for SF-36 physical component score	⊕⊝⊝⊝ - Very low quality of evidence caused by risk of bias (-2), imprecision (-1), and inconsistency (-1).	Trial Sequential Analysis for all 3 meta-analyses showed that there was not enough information to confirm or reject our anticipated intervention effects. All trials had high risk of bias, mostly because of ‘blinding of participants and personnel’, ‘incomplete outcome data bias’, and ‘for-profit bias’. All meta-analysis had high levels of heterogeneity. However, the differences were mostly between low and high intervention effects (i.e., not very serious inconsistency).
	The meta-analyses of SF-36 mental component score showed nonsignificant results (MD 3.33, Trial Sequential Analysis-adjusted confidence interval -4.47 to 11.13).			1796 (8 trials) for SF-36 mental component score		
	The meta-analysis of Minnesota Living With Heart Failure Questionnaire showed nonsignificant results (MD -7.13, 95% CI -16.19 to 1.94).			404 (6 trials) for Minnesota Living With Heart Failure Questionnaire		
Stroke	35 per 1000	34 per 1000	1.04 (0.33 to 3.28)	8114 (13 trials)	⊕⊝⊝⊝ - Very low quality of evidence caused by risk of bias (-2), imprecision (-1), and publication bias (-1).	Trial Sequential Analysis showed that there was not enough information to confirm or reject a RRR of 15% or more. All trials had high risk of bias, mostly because of ‘blinding of participants and personnel’, ‘incomplete outcome data bias’, and ‘for-profit bias’.
Ejection fraction	Rhythm control strategies versus rate control strategies significantly increased the mean ejection fraction (MD 4.20, Trial Sequential Analysis-adjusted confidence interval -2.37 to 10.77).			428 (7 trials)	⊕⊝⊝⊝ - Very low quality of evidence caused by risk of bias (-2), imprecision (-1), and inconsistency (-1).	Trial Sequential Analysis showed that there was not enough information to confirm or reject our anticipated intervention effects. All trials had high risk of bias, mostly because of ‘blinding of participants and personnel’, ‘incomplete outcome data bias’, and ‘for-profit bias’. All meta-analysis had high levels of heterogeneity. However, the differences were mostly between low and high intervention effects (i.e., not very serious inconsistency).

Summary of Findings table based on GRADE [15, 38–40]. The Summary of Findings table summarizes our main results and use five GRADE criteria (risk of bias; inconsistency of results; indirectness of evidence; imprecision; and publication bias) to assess the quality of the body of evidence.

## Discussion

We included 25 trials randomizing a total of 9354 participants with 26 comparisons of rhythm control strategies versus rate control strategies. All trials and outcome results were at high risk of bias and the quality of the evidence according to GRADE was ‘very low’ (see Summary of Findings table (Table 2)).

### Statement of principal findings

The meta-analysis of serious adverse events showed that rhythm control strategies versus rate control strategies significantly increased the risk of serious adverse events by approximately 10%, but TSA did not confirm this result. The increased risk of a serious adverse event did not seem to be driven by a particular component of the composite outcome. The meta-analyses of quality of life (SF-36 physical component score) and ejection fraction both showed a statistically significant effect in favor of the rhythm control group. However, TSAs showed that we did not have enough information to confirm or reject our anticipated intervention effects and the clinical relevance of an increase of 6.93 points on SF-36 physical component score and an increase of 4.20% in ejection fraction is questionable. No significant differences between rhythm control strategies and rate control strategies were found when assessing all-cause mortality, quality of life assessed by SF-36 mental component score, quality of life assessed by Minnesota Living with Heart Failure Questionnaire, or stroke–and all corresponding TSAs showed that there was not enough information to confirm or reject our anticipated intervention effects.

### Strengths and limitations of the systematic review

Our review has several strengths. We included more participants than any previous review which gives us increased power and precision to detect any significant difference between our compared treatment strategies [21, 22]. We followed our protocol which was registered prior to the systematic literature search [21, 22]. Data were double-extracted by independent authors minimizing the risk of inaccurate data-extraction, and we assessed the risk of bias in all trials according to Cochrane [18]. We used GRADE to assess the quality of the evidence [38–40], TSA to assess the risks of random errors [15, 16, 21, 22, 27–35], the eight-step assessment suggested by Jakobsen et al. to assess if the thresholds for significance were crossed [15], and sensitivity analyses (best-worst and worst-best) to test the potential impact of incomplete outcome data bias. Hence, this systematic review considered both risks of random errors and risks of systematic errors which adds further robustness to our results and conclusions. Another strength of our review is that we pragmatically accepted any rhythm control strategy and any rate control strategy–our results may therefore guide a clinician when choosing between the treatment strategies. The main result of this review is the apparent increased risk of a serious adverse event when using rhythm control strategies and the statistical heterogeneity of this meta-analysis result was low (I^2^ = 12% (95% CI 0 to 32%)). Hence, the included trials seem to show very similar results which increase the validity of the meta-analysis result.

As mentioned in the results section, we did not plan to include hospitalization for non-acute electrical cardioversion or hospitalization for elective antiarrhythmic drug loading as a serious adverse event, but it might be argued that any hospitalization ought to be considered a serious adverse event [24]. If we had included hospitalization for non-acute electrical cardioversion and hospitalization for elective antiarrhythmic drug loading, which in multiple trials were mandated by their protocol, the increased risk of a serious adverse event in the rhythm control group would be even greater. A post hoc meta-analysis confirmed this assumption (S32 Fig). Our results after excluding hospitalization for non-acute electrical cardioversion and hospitalization for elective antiarrhythmic drug loading as a serious adverse event were still significant which also increase the validity of our results.

Our review also has several limitations. All trials were at high risk of bias and especially the risk of incomplete blinding of participants and personnel and for-profit bias might bias our review results. Our assessment of especially publication bias was also uncertain, as a relatively low number of trials were included. Furthermore, some of the performed meta-analyses had considerable statistical heterogeneity. Hence, publication bias and heterogeneity might further bias our results. Large meta-epidemiological studies have shown that high risks of bias tend to overestimate benefits and underestimate harms of experimental interventions [106–112]. We hypothesized that the rhythm control strategy in most trials may be regarded as the experimental group and that the risk of a serious adverse event when using rhythm control strategies might be even higher than our results show due to the risk of bias. When assessing the overall quality of the available evidence, GRADE assessment showed that the quality of the evidence was ‘very low’ for all outcomes, mostly due to risk of bias, imprecision, and inconsistency (see Summary of Findings table (Table 2)). Another limitation of our present review is the use of a composite outcome such as serious adverse events. A potential limitation when using composite outcomes is that each component of a composite outcome (in this case serious adverse events) will not necessarily have similar degrees of severity and will not be affected similarly by the interventions [113]. ‘True’ differences in severity between compared groups might therefore not be reflected in review results when using composite outcomes [113]. Several of the included trials did not specify the type of serious adverse events but it was, e.g., often just reported that a given patient was hospitalized (S4 Table). Hence, it was difficult to assess severity differences between the compared groups when assessing risks of serious adverse events. We believe that the clinical relevance of the outcome ‘serious adverse events’ and the resulting increased statistical power when using serious adverse events as an outcome justifies the use of serious adverse events as a primary outcome, but the interpretative limitations ought to be considered. A further limitation of our review is that we considered rhythm control strategies and rate control strategies as two goal oriented intervention strategies. Due to widely varying interventions within the two groups, we were not able to assess the effects of each single intervention. However, even though the specific treatment elements of both rhythm control strategies and rate control strategies differed across trials (S3 Table), our results on both all-cause mortality and serious adverse events showed very limited statistical heterogeneity and test for subgroup differences showed no significant differences. Furthermore, our results show an averaged effect and if certain specific treatment elements have beneficial effects that differ from our overall meta-analysis results then other treatment elements must have more harmful effects. Nevertheless, it might be that certain single treatment elements have effects that are not shown by our analyses. The results on quality of life and ejection fraction had very large degrees of statistical heterogeneity and were especially at high risk of selective outcome reporting bias. Accordingly, these results should be interpreted with great caution.

The higher risk of a serious adverse event in the rhythm control group might be caused by incorrect use of anticoagulation therapy in the rhythm control group, i.e., physicians might avoid prescribing appropriate anticoagulation therapy if the patient has obtained sinus rhythm. We performed several subgroup analyses comparing trials with different recommendations for anticoagulation therapy (anticoagulation therapy until sinus rhythm for at least 4 weeks compared to anticoagulation therapy until sinus rhythm for at least 12 weeks compared to anticoagulation therapy until end of follow-up) (S20 Fig). No subgroup differences were found. Additionally, we found no difference between rhythm control strategies and rate control strategies when assessing stroke and the point estimate was very close to 1.00 (1.04) (Fig 8). If the participants in the rhythm control group had received insufficient anticoagulation therapy, we would have expected a higher risk of stroke in the rhythm control group.

### Strengths and limitations in relation to other systematic reviews and observational studies

We have identified multiple systematic reviews of randomized clinical trials assessing the effects of rhythm control strategies versus rate control strategies in patients with atrial fibrillation or atrial flutter [114–121]. The most recent review, made by Al-Khatib et al., was published in 2014 [114]. They included 16 trials randomizing 7608 participants and showed comparable efficacy of rhythm control strategies and rate control strategies [114]. The other previous reviews showed similar findings [115–119, 121], except Testa et al. who showed that rhythm control strategies versus rate control strategies significantly increased the risk of the combined outcome of all-cause mortality and stroke by OR at 1.15 [120]. However, their meta-analysis only included five trials randomizing 5239 participants [120]. We did not plan to assess this composite outcome but a post hoc meta-analysis assessing this composite outcome did not show any significant difference between rhythm control strategies and rate control strategies (S33 Fig). Our present review is the first systematic review of randomized clinical trials showing that rhythm control strategies versus rate control strategies significantly increases the risk of a serious adverse event. Furthermore, no clinically significant beneficial effect of rhythm control strategies versus rate control strategies was found.

We have also identified multiple observational studies assessing the effects of rhythm control strategies versus rate control strategies in patients with atrial fibrillation or atrial flutter [122–125], but these studies showed conflicting results. Comparable to our findings, Noheria et al. included 6988 participants and showed comparable efficacy of rhythm control strategies and rate control strategies when assessing all-cause mortality, heart failure, and stroke, but rhythm control strategies significantly increased the risk of cardiovascular hospitalizations [122]. Contrary to our findings, Ionescu-Ittu et al. included 26 130 participants and showed comparable efficacy of the strategies when assessing all-cause mortality within four years of treatment onset, but five and eight years after treatment onset rate control strategies significantly increased the risk of dying [123]. Furthermore, Camm et al. included 5604 participants and showed that rhythm control strategies were superior to rate control strategies [124]. A fourth study, Purmah et al., showed comparable efficacy of the strategies when assessing all-cause mortality [125]. The different results between these observational studies might be caused by, e.g., different inclusion- and exclusion criteria, baseline confounding factors, and confounding by unmeasured variables [113, 126]. Accordingly, observational studies may or may not support our findings.

### Comparison to current guidelines and recent narrative reviews

Current guidelines and recent narrative reviews recommend that a rate control strategy should be used in most patients, while a rhythm control strategy is indicated only to improve symptoms in patients who remain symptomatic on adequate rate control therapy [12, 13, 127–129]. Our results confirm this recommendation and further indicate that rhythm control strategies seem to be more harmful than rate control strategies without any meaningful beneficial effect of rhythm control strategies. Nevertheless, January et al. reports that a rhythm control strategy might be favored in specific patient subgroups. We performed several relevant subgroup analyses and found no significant differences. Moreover, no randomized clinical trial has investigated the effect of rhythm control strategies versus rate control strategies in young patients, and the other subgroup analyses had limited data. Hence, we were not able to investigate if specific patient subgroups differed compared to our main results.

### The possible contribution of ongoing trials

We identified eight ongoing trials (see S3 Table) that might contribute to the current evidence on rhythm control strategies versus rate control strategies for atrial fibrillation [103–105]. These ongoing trials will contribute to the evidence on all-cause mortality, hospitalization, stroke, quality of life, and ejection fraction. Furthermore, AFARC-LVF (NCT02509754), EAST-AFNET 4 [103], and RAFT-AF [105] will focus on the effect of catheter ablation as a rhythm control strategy. These three trials will provide evidence on whether or not catheter ablation is superior to rate control. All ongoing and future trials should be conducted with low risk of systematic error and low risk of random errors, and ought to be designed and reported according to the SPIRIT and CONSORT guidelines [130, 131].

## Conclusions

There might be specific reasons why certain patients with atrial fibrillation ought to be offered a rhythm control strategy aiming at obtaining and maintaining sinus rhythm (e.g., patients with unbearable symptoms due to atrial fibrillation, patients who are hemodynamically unstable due to atrial fibrillation, or patients who are symptomatic even after adequate rate control). Nevertheless, a rhythm control strategy often includes multiple interventions (e.g., antiarrhythmic therapy, electrical cardioversion, etc.) and our results show that rhythm control strategies seem to offer more harm than benefit in patients with atrial fibrillation. We conclude that more randomized clinical trials with low risk of bias and low risk of play of chance are needed, but based on current evidence, it seems that most patients with atrial fibrillation should be treated with a rate control strategy unless there are specific reasons justifying a rhythm control strategy.

## Differences between the protocol and the review

We changed our subgroup “age of participants: 0 to 59 years, 60–79 years, and above 80 years” to “mean age of the participants: 0 to 59 years, 60–79 years, and above 80 years”, as the former was not possible to conduct due to lack of data.

## Supporting information

S1 Text PRISMA checklist. (DOCX)

S2 Text Prepublished protocol for this systematic review. (PDF)

S3 Text Search strategy for MEDLINE (OVIDSP; 1946 to October 2016). (PDF)

S1 Fig Forest plot of ‘best-worst case’ scenario for all-cause mortality. (PDF)

S2 Fig Forest plot of ‘worst-best case’ scenario for all-cause mortality. (PDF)

S3 Fig Funnel plot of all-cause mortality. (PDF)

S4 Fig Forest plot of ‘best-worst case’ scenario for serious adverse events. (PDF)

S5 Fig Forest plot of ‘worst-best case’ scenario for serious adverse events. (PDF)

S6 Fig Funnel plot for serious adverse events. (PDF)

S7 Fig Forest plot for myocardial infarction. (PDF)

S8 Fig Forest plot for heart failure. (PDF)

S9 Fig Forest plot for ventricular tachycardia. (PDF)

S10 Fig Forest plot for hospitalization (excluding hospitalization for non-acute electrical cardioversion and hospitalization for elective antiarrhythmic drug loading). (PDF)

S11 Fig Forest plot of the comparison of different types of rhythm control interventions for all-cause mortality. (PDF)

S12 Fig Forest plot of the comparison of different types of rate control interventions for all-cause mortality. (PDF)

S13 Fig Forest plot of the comparison of trials with different mean ages for all-cause mortality. (PDF)

S14 Fig Forest plot of the comparison of different durations of atrial fibrillation for all-cause mortality. (PDF)

S15 Fig Forest plot of the comparison of different durations of anticoagulation therapy for all-cause mortality. (PDF)

S16 Fig Forest plot of the comparison of different types of rhythm control interventions for serious adverse events. (PDF)

S17 Fig Forest plot of the comparison of different types of rate control interventions for serious adverse events. (PDF)

S18 Fig Forest plot of the comparison of trials with different mean ages for serious adverse events. (PDF)

S19 Fig Forest plot of the comparison of trials with different durations of atrial fibrillation for serious adverse events. (PDF)

S20 Fig Forest plot of the comparison of trials with different durations of anticoagulation therapy for serious adverse events. (PDF)

S21 Fig Forest plot of the comparison of participants with heart failure to participants without heart failure for all-cause mortality. (PDF)

S22 Fig Forest plot of the comparison of participants with heart failure to participants without heart failure for serious adverse events. (PDF)

S23 Fig Forest plot of ‘best-worst case’ scenario for quality of life (SF-36 physical component score). (PDF)

S24 Fig Forest plot of ‘worst-best case’ scenario for quality of life (SF-36 physical component score). (PDF)

S25 Fig Forest plot for quality of life (SF-36 mental component score). (PDF)

S26 Fig Forest plot for quality of life (Minnesota Living With Heart Failure Questionnaire). (PDF)

S27 Fig Trial Sequential Analysis for Quality of life (SF-36 mental component score). (PNG)

S28 Fig Forest plot of ‘best-worst case’ scenario for stroke. (PDF)

S29 Fig Forest plot of ‘worst-best case’ scenario for stroke. (PDF)

S30 Fig Forest plot of ‘best-worst case’ scenario for ejection fraction. (PDF)

S31 Fig Forest plot of ‘worst-best case’ scenario for ejection fraction. (PDF)

S32 Fig Forest plot for serious adverse events with hospitalization (including hospitalization for non-acute electrical cardioversion and hospitalization for elective antiarrhythmic drug loading). (PDF)

S33 Fig Forest plot of the composite outcome for all-cause mortality and stroke. (PDF)

S1 Table Bias risk assessment of each included trial. (DOCX)

S2 Table Inclusion- and exclusion criteria for each included trial. (DOCX)

S3 Table Trial characteristics of each included, excluded, and ongoing trial. (DOCX)

S4 Table Specific types of serious adverse events in each trial. (DOCX)

## References

[1] Pistoia F, Sacco S, Tiseo C, Degan D, Ornello R, Carolei A. The epidemiology of atrial fibrillation and stroke. Cardiology Clinics. 2016;34(2):255–68. doi: 10.1016/j.ccl.2015.12.002 27150174 10.1016/j.ccl.2015.12.002

[2] Camm AJ, Lip GY, De Caterina R, Savelieva I, Atar D, Hohnloser SH, et al 2012 focused update of the ESC Guidelines for the management of atrial fibrillation: an update of the 2010 ESC Guidelines for the management of atrial fibrillation. Europace. 2012;14(10):1385–413. doi: 10.1093/europace/eus305 22923145 10.1093/europace/eus305

[3] Stewart S, Hart CL, Hole DJ, McMurray JJ. A population-based study of the long-term risks associated with atrial fibrillation: 20-year follow-up of the Renfrew/Paisley study. American Journal of Medicine. 2002;113(5):359–64. 12401529 10.1016/s0002-9343(02)01236-6

[4] Benjamin EJ, Wolf PA, D'Agostino RB, Silbershatz H, Kannel WB, Levy D. Impact of atrial fibrillation on the risk of death: the Framingham Heart Study. Circulation. 1998;98(10):946–52. 9737513 10.1161/01.cir.98.10.946

[5] Rahman F, Wang N, Yin X, Ellinor PT, Lubitz SA, LeLorier PA, et al Atrial flutter: clinical risk factors and adverse outcomes in the Framingham Heart Study. Heart Rhythm. 2016;13(1):233–40. doi: 10.1016/j.hrthm.2015.07.031 26226213 10.1016/j.hrthm.2015.07.031 PMC4698205

[6] Healey JS, Oldgren J, Ezekowitz M, Zhu J, Pais P, Wang J, et al Occurrence of death and stroke in patients in 47 countries 1 year after presenting with atrial fibrillation: a cohort study. Lancet. 2016. 10.1016/S0140-6736(16)30968-0 27515684

[7] Wolf PA, Abbott RD, Kannel WB. Atrial fibrillation as an independent risk factor for stroke: the Framingham Study. Stroke. 1991;22(8):983–8. 1866765 10.1161/01.str.22.8.983

[8] Odutayo A, Wong CX, Hsiao AJ, Hopewell S, Altman DG, Emdin CA. Atrial fibrillation and risks of cardiovascular disease, renal disease, and death: systematic review and meta-analysis. BMJ. 2016;354:i4482 doi: 10.1136/bmj.i4482 27599725 10.1136/bmj.i4482

[9] Go AS, Hylek EM, Phillips KA, Chang Y, Henault LE, Selby JV, et al Prevalence of diagnosed atrial fibrillation in adults: national implications for rhythm management and stroke prevention: the Anticoagulation and Risk Factors in Atrial Fibrillation (ATRIA) Study. JAMA. 2001;285(18):2370–5. 11343485 10.1001/jama.285.18.2370

[10] Stewart S, Murphy NF, Walker A, McGuire A, McMurray JJ. Cost of an emerging epidemic: an economic analysis of atrial fibrillation in the UK. Heart. 2004;90(3):286–92. doi: 10.1136/hrt.2002.008748 14966048 10.1136/hrt.2002.008748 PMC1768125

[11] Mozaffarian D, Benjamin EJ, Go AS, Arnett DK, Blaha MJ, Cushman M, et al Heart disease and stroke statistics-2016 Update: a report from the American Heart Association. Circulation. 2016;133(4):e38–360. doi: 10.1161/CIR.0000000000000350 26673558 10.1161/CIR.0000000000000350

[12] January CT, Wann LS, Alpert JS, Calkins H, Cigarroa JE, Cleveland JC Jr., et al 2014 AHA/ACC/HRS guideline for the management of patients with atrial fibrillation: a report of the American College of Cardiology/American Heart Association Task Force on Practice Guidelines and the Heart Rhythm Society. Journal of the American College of Cardiology. 2014;64(21):e1–76. doi: 10.1016/j.jacc.2014.03.022 24685669 10.1016/j.jacc.2014.03.022

[13] Kirchhof P, Benussi S, Kotecha D, Ahlsson A, Atar D, Casadei B, et al 2016 ESC Guidelines for the management of atrial fibrillation developed in collaboration with EACTS: The Task Force for the management of atrial fibrillation of the European Society of Cardiology (ESC). Developed with the special contribution of the European Heart Rhythm Association (EHRA) of the ESC. Endorsed by the European Stroke Organisation (ESO). European Heart Journal. 2016.

[14] Markides V, Schilling RJ. Atrial fibrillation: classification, pathophysiology, mechanisms and drug treatment. Heart. 2003;89(8):939–43. 12860883 10.1136/heart.89.8.939 PMC1767799

[15] Jakobsen JC, Wetterslev J, Winkel P, Lange T, Gluud C. Thresholds for statistical and clinical significance in systematic reviews with meta-analytic methods. BMC Medical Research Methodology. 2014;14:120 doi: 10.1186/1471-2288-14-120 25416419 10.1186/1471-2288-14-120 PMC4251848

[16] Wetterslev J, Thorlund K, Brok J, Gluud C. Trial sequential analysis may establish when firm evidence is reached in cumulative meta-analysis. Journal of Clinical Epidemiology. 2008;61(1):64–75. doi: 10.1016/j.jclinepi.2007.03.013 18083463 10.1016/j.jclinepi.2007.03.013

[17] Keus F, Wetterslev J, Gluud C, van Laarhoven CJ. Evidence at a glance: error matrix approach for overviewing available evidence. BMC Medical Research Methodology. 2010;10:90 doi: 10.1186/1471-2288-10-90 20920306 10.1186/1471-2288-10-90 PMC2959031

[18] Higgins J, Green S. Cochrane Handbook for Systematic Reviews of Interventions Version 5.1.0 The Cochrane Collaboration 2011; www.handbook.cochrane.org.

[19] Moher D, Liberati A, Tetzlaff J, Altman DG. Preferred reporting items for systematic reviews and meta-analyses: the PRISMA statement. Annals of Internal Medicine. 2009;151(4):264–9, w64. 19622511 10.7326/0003-4819-151-4-200908180-00135

[20] Liberati A, Altman DG, Tetzlaff J, Mulrow C, Gotzsche PC, Ioannidis JP, et al The PRISMA statement for reporting systematic reviews and meta-analyses of studies that evaluate healthcare interventions: explanation and elaboration. BMJ. 2009;339:b2700 doi: 10.1136/bmj.b2700 19622552 10.1136/bmj.b2700 PMC2714672

[21] Sethi N, Safi S, Nielsen E, Feinberg J, Gluud C, Jakobsen JC. Interventions aiming at rate control versus rhythm control for atrial fibrillation and atrial flutter. A protocol for a systematic review with meta-analysis and trial sequential analysis. PROSPERO. 2016:CRD42016051433. 10.1186/s13643-017-0449-z PMC5340010 28264715

[22] Sethi NJ, Safi S, Nielsen EE, Feinberg J, Gluud C, Jakobsen JC. The effects of rhythm control strategies versus rate control strategies for atrial fibrillation and atrial flutter: a protocol for a systematic review with meta-analysis and Trial Sequential Analysis. Systematic Reviews. 2017;6(1):47 doi: 10.1186/s13643-017-0449-z 28264715 10.1186/s13643-017-0449-z PMC5340010

[23] Lundh A, Lexchin J, Mintzes B, Schroll JB, Bero L. Industry sponsorship and research outcome. Cochrane Database of Systematic Reviews. 2017;2:Mr000033 doi: 10.1002/14651858.MR000033.pub3 28207928 10.1002/14651858.MR000033.pub3 PMC8132492

[24] International conference on harmonisation of technical requirements for registration of pharmaceuticals for human use (ICH) adopts consolidated guideline on good clinical practice in the conduct of clinical trials on medicinal products for human use. International Digest of Health Legislation. 1997;48(2):231–4. 11656783

[25] Review Manager (RevMan) [Computer program]. Version 5.3. Copenhagen: The Nordic Cochrane Centre, The Cochrane Collaboration. 2014.

[26] StataCorp. Stata: Release 14. Statistical Software. College Station, TX: StataCorp LP 2014; http://www.stata.com.

[27] Copenhagen Trial Unit. TSA—Trial Sequential Analysis. http://www.ctu.dk/tsa/.

[28] Brok J, Thorlund K, Gluud C, Wetterslev J. Trial sequential analysis reveals insufficient information size and potentially false positive results in many meta-analyses. Journal of Clinical Epidemiology. 2008;61(8):763–9. doi: 10.1016/j.jclinepi.2007.10.007 18411040 10.1016/j.jclinepi.2007.10.007

[29] Brok J, Thorlund K, Wetterslev J, Gluud C. Apparently conclusive meta-analyses may be inconclusive—Trial sequential analysis adjustment of random error risk due to repetitive testing of accumulating data in apparently conclusive neonatal meta-analyses. International Journal of Epidemiology. 2009;38(1):287–98. doi: 10.1093/ije/dyn188 18824466 10.1093/ije/dyn188

[30] Thorlund K, Devereaux PJ, Wetterslev J, Guyatt G, Ioannidis JP, Thabane L, et al Can trial sequential monitoring boundaries reduce spurious inferences from meta-analyses? International Journal of Epidemiology. 2009;38(1):276–86. doi: 10.1093/ije/dyn179 18824467 10.1093/ije/dyn179

[31] Wetterslev J, Thorlund K, Brok J, Gluud C. Estimating required information size by quantifying diversity in random-effects model meta-analyses. BMC Medical Research Methodology. 2009;9:86 doi: 10.1186/1471-2288-9-86 20042080 10.1186/1471-2288-9-86 PMC2809074

[32] Thorlund K, Anema A, Mills E. Interpreting meta-analysis according to the adequacy of sample size. An example using isoniazid chemoprophylaxis for tuberculosis in purified protein derivative negative HIV-infected individuals. Clinical Epidemiology. 2010;2:57–66. 20865104 10.2147/clep.s9242 PMC2943189

[33] Thorlund K EJ, Wetterslev J, Brok J, Imberger G, Gluud C. User manual for Trial Sequential Analysis (TSA). 2011. http://www.ctu.dk/tsa/files/tsa_manual.pdf.

[34] Imberger G, Gluud C, Boylan J, Wetterslev J. Systematic reviews of anesthesiologic interventions reported as statistically significant: problems with power, precision, and type 1 error protection. Anesthesia and Analgesia. 2015;121(6):1611–22. doi: 10.1213/ANE.0000000000000892 26579662 10.1213/ANE.0000000000000892

[35] Imberger G, Thorlund K, Gluud C, Wetterslev J. False-positive findings in Cochrane meta-analyses with and without application of trial sequential analysis: an empirical review. BMJ Open. 2016;6(8):e011890 doi: 10.1136/bmjopen-2016-011890 27519923 10.1136/bmjopen-2016-011890 PMC4985805

[36] Norman GR, Sloan JA, Wyrwich KW. Interpretation of changes in health-related quality of life: the remarkable universality of half a standard deviation. Medical Care. 2003;41(5):582–92. doi: 10.1097/01.MLR.0000062554.74615.4C 12719681 10.1097/01.MLR.0000062554.74615.4C

[37] Jakobsen JC, Wetterslev J, Lange T, Gluud C. Viewpoint: taking into account risks of random errors when analysing multiple outcomes in systematic reviews. Cochrane Database of Systematic Reviews. 2016;3:Ed000111 doi: 10.1002/14651858.ED000111 27030037 10.1002/14651858.ED000111 PMC10845867

[38] Guyatt GH, Oxman AD, Vist GE, Kunz R, Falck-Ytter Y, Alonso-Coello P, et al GRADE: an emerging consensus on rating quality of evidence and strength of recommendations. BMJ. 2008;336(7650):924–6. doi: 10.1136/bmj.39489.470347.AD 18436948 10.1136/bmj.39489.470347.AD PMC2335261

[39] Schunemann HJ, Best D, Vist G, Oxman AD. Letters, numbers, symbols and words: how to communicate grades of evidence and recommendations. CMAJ. 2003;169(7):677–80. 14517128 PMC202287

[40] Guyatt GH, Oxman AD, Schunemann HJ, Tugwell P, Knottnerus A. GRADE guidelines: a new series of articles in the Journal of Clinical Epidemiology. Journal of Clinical Epidemiology. 2011;64(4):380–2. doi: 10.1016/j.jclinepi.2010.09.011 21185693 10.1016/j.jclinepi.2010.09.011

[41] Roy D, Talajic M, Nattel S, Wyse DG, Dorian P, Lee KL, et al Rhythm control versus rate control for atrial fibrillation and heart failure. NEJM. 2008;358(25):2667–77. doi: 10.1056/NEJMoa0708789 18565859 10.1056/NEJMoa0708789

[42] Suman-Horduna I, Roy D, Frasure-Smith N, Talajic M, Lesperance F, Blondeau L, et al Quality of life and functional capacity in patients with atrial fibrillation and congestive heart failure. Journal of the American College of Cardiology. 2013;61(4):455–60. doi: 10.1016/j.jacc.2012.10.031 23265334 10.1016/j.jacc.2012.10.031

[43] Rationale and design of a study assessing treatment strategies of atrial fibrillation in patients with heart failure: the Atrial Fibrillation and Congestive Heart Failure (AF-CHF) trial. American Heart Journal. 2002;144(4):597–607. 12360154

[44] Talajic M, Khairy P, Levesque S, Connolly SJ, Dorian P, Dubuc M, et al Maintenance of sinus rhythm and survival in patients with heart failure and atrial fibrillation. Journal of the America College of Cardiology. 2010;55(17):1796–802. 10.1016/j.jacc.2010.01.023 20413028

[45] Roy D. Rationale for the Atrial Fibrillation and Congestive Heart Failure (AF-CHF) trial. Cardiac Electrophysiology Review. 2003;7(3):208–10. doi: 10.1023/B:CEPR.0000012383.63580.c8 14739714 10.1023/B:CEPR.0000012383.63580.c8

[46] Chung MK, Shemanski L, Sherman DG, Greene HL, Hogan DB, Kellen JC, et al Functional status in rate- versus rhythm-control strategies for atrial fibrillation: results of the Atrial Fibrillation Follow-Up Investigation of Rhythm Management (AFFIRM) functional status substudy. Journal of the American College of Cardiology. 2005;46(10):1891–9. doi: 10.1016/j.jacc.2005.07.040 16286177 10.1016/j.jacc.2005.07.040

[47] Sherman DG, Kim SG, Boop BS, Corley SD, Dimarco JP, Hart RG, et al Occurrence and characteristics of stroke events in the Atrial Fibrillation Follow-up Investigation of Sinus Rhythm Management (AFFIRM) study. Archives of Internal Medicine. 2005;165(10):1185–91. doi: 10.1001/archinte.165.10.1185 15911734 10.1001/archinte.165.10.1185

[48] Steinberg JS, Sadaniantz A, Kron J, Krahn A, Denny DM, Daubert J, et al Analysis of cause-specific mortality in the Atrial Fibrillation Follow-up Investigation of Rhythm Management (AFFIRM) study. Circulation. 2004;109(16):1973–80. doi: 10.1161/01.CIR.0000118472.77237.FA 15051639 10.1161/01.CIR.0000118472.77237.FA

[49] Jenkins LS, Brodsky M, Schron E, Chung M, Rocco T Jr., Lader E, et al Quality of life in atrial fibrillation: the Atrial Fibrillation Follow-up Investigation of Rhythm Management (AFFIRM) study. American Heart Journal. 2005;149(1):112–20. doi: 10.1016/j.ahj.2004.03.065 15660042 10.1016/j.ahj.2004.03.065

[50] Wyse DG, Waldo AL, DiMarco JP, Domanski MJ, Rosenberg Y, Schron EB, et al A comparison of rate control and rhythm control in patients with atrial fibrillation. NEJM. 2002;347(23):1825–33. doi: 10.1056/NEJMoa021328 12466506 10.1056/NEJMoa021328

[51] Investigators AFFIRM. Baseline characteristics of patients with atrial fibrillation: the AFFIRM Study. American Heart Journal. 2002;143(6):991–1001. 12075254 10.1067/mhj.2002.122875

[52] Wyse DG, Anderson JL, Antman EM, Cooper ES, Dalquist JE, Davis KB, et al Atrial fibrillation follow-up investigation of rhythm management—the AFFIRM study design. The Planning and Steering Committees of the AFFIRM study for the NHLBI AFFIRM investigators. American Journal of Cardiology. 1997;79(9):1198–202. 9164885

[53] Curtis AB, Gersh BJ, Corley SD, DiMarco JP, Domanski MJ, Geller N, et al Clinical factors that influence response to treatment strategies in atrial fibrillation: the Atrial Fibrillation Follow-up Investigation of Rhythm Management (AFFIRM) Study. American Heart Journal. 2005;149(4):645–9. doi: 10.1016/j.ahj.2004.09.038 15990747 10.1016/j.ahj.2004.09.038

[54] Damluji AA, Al-Damluji MS, Marzouka GR, Coffey JO, Viles-Gonzalez JF, Cohen MG, et al New-onset versus prior history of atrial fibrillation: Outcomes from the AFFIRM trial. American Heart Journal. 2015;170(1):156–63. doi: 10.1016/j.ahj.2015.04.020 26093877 10.1016/j.ahj.2015.04.020

[55] Guglin M, Chen R, Curtis AB. Sinus rhythm is associated with fewer heart failure symptoms: insights from the AFFIRM trial. Heart Rhythm. 2010;7(5):596–601. doi: 10.1016/j.hrthm.2010.01.003 20159046 10.1016/j.hrthm.2010.01.003

[56] Shariff N, Desai RV, Patel K, Ahmed MI, Fonarow GC, Rich MW, et al Rate-control versus rhythm-control strategies and outcomes in septuagenarians with atrial fibrillation. American Journal of Medicine. 2013;126(10):887–93. doi: 10.1016/j.amjmed.2013.04.021 24054956 10.1016/j.amjmed.2013.04.021 PMC3818786

[57] Brignole M, Gianfranchi L, Menozzi C, Alboni P, Musso G, Bongiorni MG, et al Assessment of atrioventricular junction ablation and DDDR mode-switching pacemaker versus pharmacological treatment in patients with severely symptomatic paroxysmal atrial fibrillation: a randomized controlled study. Circulation. 1997;96(8):2617–24. 9355902 10.1161/01.cir.96.8.2617

[58] Shelton RJ, Clark AL, Goode K, Rigby AS, Houghton T, Kaye GC, et al A randomised, controlled study of rate versus rhythm control in patients with chronic atrial fibrillation and heart failure: (CAFE-II Study). Heart. 2009;95(11):924–30. doi: 10.1136/hrt.2008.158931 19282313 10.1136/hrt.2008.158931

[59] Hunter RJ, Berriman TJ, Diab I, Kamdar R, Richmond L, Baker V, et al A randomized controlled trial of catheter ablation versus medical treatment of atrial fibrillation in heart failure (the CAMTAF trial). Circulation: Arrhythmia and Electrophysiology. 2014;7(1):31–8. 24382410 10.1161/CIRCEP.113.000806

[60] Vora A, Karnad D, Goyal V, Naik A, Gupta A, Lokhandwala Y, et al Control of heart rate versus rhythm in rheumatic atrial fibrillation: a randomized study. Journal of Cardiovascular Pharmacology and Therapeutics. 2004;9(2):65–73. doi: 10.1177/107424840400900201 15309242 10.1177/107424840400900201

[61] Fengsrud E, Wickbom A, Almroth H, Englund A, Ahlsson A. Total endoscopic ablation of patients with long-standing persistent atrial fibrillation: a randomized controlled study. Interactive CardioVascular and Thoracic Surgery. 2016;23(2):292–8. doi: 10.1093/icvts/ivw088 27068249 10.1093/icvts/ivw088

[62] Gillinov AM, Bagiella E, Moskowitz AJ, Raiten JM, Groh MA, Bowdish ME, et al Rate control versus rhythm control for atrial fibrillation after cardiac surgery. NEJM. 2016;374(20):1911–21. doi: 10.1056/NEJMoa1602002 27043047 10.1056/NEJMoa1602002 PMC4908812

[63] Opolski G, Torbicki A, Kosior D, Szulc M, Zawadzka M, Pierscinska M, et al Rhythm control versus rate control in patients with persistent atrial fibrillation. Results of the HOT CAFE Polish Study. Kardiol Pol. 2003;59(7):1–16. 14560344

[64] Pietrasik A, Kosior DA, Niewada M, Opolski G, Latek M, Kaminski B. The cost comparison of rhythm and rate control strategies in persistent atrial fibrillation. International Journal of Cardiology. 2007;118(1):21–7. doi: 10.1016/j.ijcard.2006.03.085 17055081 10.1016/j.ijcard.2006.03.085

[65] Szulc M, Kosior DA, Jasik M, Wozakowska-Kaplon B, Janion M, Rabczenko D, et al The effect of rate versus rhythm control strategy on the left ventricular function in patients with persistent atrial fibrillation: results of one year follow-up. Folia Cardiologica. 2006;13(4):331–7.

[66] Hu CL, Jiang H, Tang QZ, Zhang QH, Chen JB, Huang CX, et al Comparison of rate control and rhythm control in patients with atrial fibrillation after percutaneous mitral balloon valvotomy: a randomised controlled study. Heart. 2006;92(8):1096–101. doi: 10.1136/hrt.2005.080325 16387819 10.1136/hrt.2005.080325 PMC1861118

[67] Ogawa S, Yamashita T, Yamazaki T, Aizawa Y, Atarashi H, Inoue H, et al Optimal treatment strategy for patients with paroxysmal atrial fibrillation: J-RHYTHM Study. Circulation Journal. 2009;73(2):242–8. 19060419 10.1253/circj.cj-08-0608

[68] Yamashita T, Ogawa S, Aizawa Y, Atarashi H, Inoue H, Ohe T, et al Investigation of the optimal treatment strategy for atrial fibrillation in Japan. Circulation Journal. 2003;67(9):738–41. 12939546 10.1253/circj.67.738

[69] Jones DG, Haldar SK, Hussain W, Sharma R, Francis DP, Rahman-Haley SL, et al A randomized trial to assess catheter ablation versus rate control in the management of persistent atrial fibrillation in heart failure. Journal of the American College of Cardiology. 2013;61(18):1894–903. doi: 10.1016/j.jacc.2013.01.069 23500267 10.1016/j.jacc.2013.01.069

[70] Lee JK, Klein GJ, Krahn AD, Yee R, Zarnke K, Simpson C, et al Rate-control versus conversion strategy in postoperative atrial fibrillation: trial design and pilot study results. Cardiac Electrophysiology Review. 2003;7(2):178–84. 14618047 10.1023/a:1027428003609

[71] Lee JK, Klein GJ, Krahn AD, Yee R, Zarnke K, Simpson C, et al Rate-control versus conversion strategy in postoperative atrial fibrillation: a prospective, randomized pilot study. American Heart Journal. 2000;140(6):871–7. doi: 10.1067/mhj.2000.111104 11099990 10.1067/mhj.2000.111104

[72] MacDonald MR, Connelly DT, Hawkins NM, Steedman T, Payne J, Shaw M, et al Radiofrequency ablation for persistent atrial fibrillation in patients with advanced heart failure and severe left ventricular systolic dysfunction: a randomised controlled trial. Heart. 2011;97(9):740–7. doi: 10.1136/hrt.2010.207340 21051458 10.1136/hrt.2010.207340

[73] Marshall HJ, Harris ZI, Griffith MJ, Holder RL, Gammage MD. Prospective randomized study of ablation and pacing versus medical therapy for paroxysmal atrial fibrillation: effects of pacing mode and mode-switch algorithm. Circulation. 1999;99(12):1587–92. 10096935 10.1161/01.cir.99.12.1587

[74] Khan MN, Jais P, Cummings J, Di Biase L, Sanders P, Martin DO, et al Pulmonary-vein isolation for atrial fibrillation in patients with heart failure. NEJM. 2008;359(17):1778–85. doi: 10.1056/NEJMoa0708234 18946063 10.1056/NEJMoa0708234

[75] Brignole M, Menozzi C, Gasparini M, Bongiorni MG, Botto GL, Ometto R, et al An evaluation of the strategy of maintenance of sinus rhythm by antiarrhythmic drug therapy after ablation and pacing therapy in patients with paroxysmal atrial fibrillation. European Heart Journal. 2002;23(11):892–900. doi: 10.1053/euhj.2001.2971 12042011 10.1053/euhj.2001.2971

[76] Brignole M. Rhythm versus rate control after ablation and pacing for paroxysmal atrial fibrillation: clinical implications of the PAF 2 trial. Cardiac Electrophysiology Review. 2003;7(2):127–9. 14618035 10.1023/a:1027403330926

[77] Gronefeld GC, Lilienthal J, Kuck KH, Hohnloser SH. Impact of rate versus rhythm control on quality of life in patients with persistent atrial fibrillation. Results from a prospective randomized study. European Heart Journal. 2003;24(15):1430–6. 12909072 10.1016/s0195-668x(03)00261-6

[78] Hohnloser SH, Kuck KH. Atrial fibrillation: maintaining stability of sinus rhythm or ventricular rate control? The need for prospective data: the PIAF trial. Pacing and Clinical Electrophysiology. 1997;20(8 Pt 1):1989–92. 9272538 10.1111/j.1540-8159.1997.tb03606.x

[79] Hohnloser SH, Kuck KH, Lilienthal J. Rhythm or rate control in atrial fibrillation—Pharmacological Intervention in Atrial Fibrillation (PIAF): a randomised trial. Lancet. 2000;356(9244):1789–94. 11117910 10.1016/s0140-6736(00)03230-x

[80] Soucier R, Silverman D, Abordo M, Jaagosild P, Abiose A, Madhusoodanan KP, et al Propafenone versus ibutilide for post operative atrial fibrillation following cardiac surgery: neither strategy improves outcomes compared to rate control alone (the PIPAF study). Medical Science Monitor. 2003;9(3):Pi19–23. 12640352

[81] Hagens VE, Crijns HJ, Van Veldhuisen DJ, Van Den Berg MP, Rienstra M, Ranchor AV, et al Rate control versus rhythm control for patients with persistent atrial fibrillation with mild to moderate heart failure: results from the RAte Control versus Electrical cardioversion (RACE) study. American Heart Journal. 2005;149(6):1106–11. doi: 10.1016/j.ahj.2004.11.030 15976795 10.1016/j.ahj.2004.11.030

[82] Hagens VE, Ranchor AV, Van Sonderen E, Bosker HA, Kamp O, Tijssen JG, et al Effect of rate or rhythm control on quality of life in persistent atrial fibrillation. Results from the Rate Control Versus Electrical Cardioversion (RACE) Study. Journal of the American College of Cardiology. 2004;43(2):241–7. 14736444 10.1016/j.jacc.2003.08.037

[83] Rienstra M, Van Veldhuisen DJ, Crijns HJ, Van Gelder IC. Enhanced cardiovascular morbidity and mortality during rhythm control treatment in persistent atrial fibrillation in hypertensives: data of the RACE study. European Heart Journal. 2007;28(6):741–51. doi: 10.1093/eurheartj/ehl436 17272359 10.1093/eurheartj/ehl436

[84] Rienstra M, Van Veldhuisen DJ, Hagens VE, Ranchor AV, Veeger NJ, Crijns HJ, et al Gender-related differences in rhythm control treatment in persistent atrial fibrillation: data of the Rate Control Versus Electrical Cardioversion (RACE) study. Journal of the American College of Cardiology. 2005;46(7):1298–306. doi: 10.1016/j.jacc.2005.05.078 16198847 10.1016/j.jacc.2005.05.078

[85] Van Gelder IC, Hagens VE, Bosker HA, Kingma JH, Kamp O, Kingma T, et al A comparison of rate control and rhythm control in patients with recurrent persistent atrial fibrillation. NEJM. 2002;347(23):1834–40. doi: 10.1056/NEJMoa021375 12466507 10.1056/NEJMoa021375

[86] Van Gelder IC, Hagens VE, Kingma JH, Bosker HA, Kamp O, Kingma T, et al Rate control versus electrical cardioversion for atrial fibrillation: A randomised comparison of two treatment strategies concerning morbidity, mortality, quality of life and cost-benefit—the RACE study design. Netherlands Heart Journal. 2002;10(3):118–24. 25696077 PMC2499705

[87] Hagens VE, Rienstra M, Van Veldhuisen DJ, Crijns H, Van Gelder IC. Determinants of sudden cardiac death in patients with persistent atrial fibrillation in the rate control versus electrical cardioversion (RACE) study. American Journal of Cardiology. 2006;98(7):929–32. doi: 10.1016/j.amjcard.2006.04.038 16996876 10.1016/j.amjcard.2006.04.038

[88] Hagens VE, Van Veldhuisen DJ, Kamp O, Rienstra M, Bosker HA, Veeger NJ, et al Effect of rate and rhythm control on left ventricular function and cardiac dimensions in patients with persistent atrial fibrillation: results from the rate control versus electrical cardioversion for persistent atrial fibrillation (RACE) Study. Heart Rhythm. 2005;2(1):19–24. doi: 10.1016/j.hrthm.2004.09.028 15851259 10.1016/j.hrthm.2004.09.028

[89] Rienstra M, Hagens VE, Van Veldhuisen DJ, Bosker HA, Tijssen JG, Kamp O, et al Clinical characteristics of persistent lone atrial fibrillation in the RACE study. American Journal of Cardiology. 2004;94(12):1486–90. doi: 10.1016/j.amjcard.2004.08.024 15589001 10.1016/j.amjcard.2004.08.024

[90] Rafla SM, Mahmoud K, Kak A, Lotfy M, Soghier I. Comparison of effect of rate control versus rhythm control on LV function in patients with AF and ischemic heart failure. Journal of the Saudi Heart Association. 2013;25(2):165.

[91] Carlsson J, Boos C. Confounding factors in rate versus rhythm control trials in patients with atrial fibrillation: lessons from the strategies of treatment of atrial fibrillation (STAF) pilot study. Cardiac Electrophysiology Review. 2003;7(2):122–6. 14618034 10.1023/a:1027499114087

[92] Carlsson J, Miketic S, Windeler J, Cuneo A, Haun S, Micus S, et al Randomized trial of rate-control versus rhythm-control in persistent atrial fibrillation: the Strategies of Treatment of Atrial Fibrillation (STAF) study. Journal of the American College of Cardiology. 2003;41(10):1690–6. 12767648 10.1016/s0735-1097(03)00332-2

[93] Vijayvergiya R, Jain S, Bahl A. Comparison of rate versus rhyhm control after percutaneous transluminal mitral commissurotomy in patients with chronic atrial fibrillation: a prospective randomized comparative study. American Journal of Cardiology. 2009;103(9A):115B.19101240

[94] Yildiz A, Yigit Z, Okcun B, Baskurt M, Ortak K, Kaya A, et al Comparison of rate and rhythm control in hypertension patients with atrial fibrillation. Circulation Journal. 2008;72(5):705–8. 18441447 10.1253/circj.72.705

[95] Okcun B, Yigit Z, Arat A, Kucukoglu SM. Comparison of rate and rhythm control in patients with atrial fibrillation and nonischemic heart failure. Japanese Heart Journal. 2004;45(4):591–601. 15353870 10.1536/jhj.45.591

[96] Opolski G, Torbicki A, Kosior D, Stolarz P, Dawidowska R, Zawadzka M, et al [Should sinus rhythm be restored in patients with chronic atrial fibrillation? Preliminary results from the Polish "Hot Cafe" study]. [Polish]. Polish Archives of Internal Medicine. 1999;101(5):413–8. 10740421

[97] Beaver TM, Hedna VS, Khanna AY, Miles WM, Price CC, Schmalfuss IM, et al Thoracoscopic ablation with appendage ligation versus medical therapy for stroke prevention: a proof-of-concept randomized trial. Innovations (Phila). 2016;11(2):99–105. 26914668 10.1097/IMI.0000000000000226 PMC6545892

[98] Kanorskii SG, Kruchinova OA, Zingilevskii K. [Advantages of restoration and maintenance of sinus rhythm in middle aged patients with atrial fibrillation and chronic heart failure]. Kardiologiia. 2006;46(9):31–5. 17047620

[99] Kirkutis A, Poviliunas A, Griciene P, Polena S, Yang S, Yalamanchi G, et al Cardiac rate normalization in chronic atrial fibrillation: comparison of long-term efficacy of treatment with amiodarone versus AV node ablation and permanent His-bundle pacing. Proceedings of the Western Pharmacology Society. 2004;47:69–70. 15633616

[100] Petrac D, Radic B, Radeljic V, Hamel D, Filipovic J. Ventricular pacing vs dual chamber pacing in patients with persistent atrial fibrillation after atrioventricular node ablation: open randomized study. Croatian Medical Journal. 2005;46(6):922–8. 16342345

[101] Schwartzman D, Housel D, Bazaz R, Jain S, Saba S, Gorcsan J 3rd, et al A pilot study to assess benefit of atrial rhythm control after cardiac resynchronization therapy and atrioventricular node ablation. Pacing and Clinical Electrophysiology. 2015;38(2):275–81. doi: 10.1111/pace.12535 25431023 10.1111/pace.12535

[102] Okcun B, Yigit Z, Yildiz A, Uzunhasan I, Orta K, Baskurt M, et al What should be the primary treatment in atrial fibrillation: ventricular rate control or sinus rhythm control with long-term anticoagulation? Journal of International Medical Research. 2009;37(2):464–71. doi: 10.1177/147323000903700222 19383241 10.1177/147323000903700222

[103] Kirchhof P, Breithardt G, Camm AJ, Crijns HJ, Kuck KH, Vardas P, et al Improving outcomes in patients with atrial fibrillation: rationale and design of the Early treatment of Atrial fibrillation for Stroke prevention Trial. American Heart Journal. 2013;166(3):442–8. doi: 10.1016/j.ahj.2013.05.015 24016492 10.1016/j.ahj.2013.05.015

[104] Ciszewski J, Maciag A, Kowalik I, Syska P, Lewandowski M, Farkowski MM, et al Comparison of the rhythm control treatment strategy versus the rate control strategy in patients with permanent or long-standing persistent atrial fibrillation and heart failure treated with cardiac resynchronization therapy—a pilot study of Cardiac Resynchronization in Atrial Fibrillation Trial (Pilot-CRAfT): study protocol for a randomized controlled trial. Trials. 2014;15:386 doi: 10.1186/1745-6215-15-386 25281275 10.1186/1745-6215-15-386 PMC4283117

[105] Tang A, Essebag V, Leong-Sit P, Sterns LD, Wilton SB, Parkash R, et al How well are rate or rhythm control achieved in raft-AF patients with atrial fibrillation and heart failure? Canadian Journal of Cardiology. 2015;31(10):S254–S5.

[106] Gluud LL. Bias in clinical intervention research. American Journal of Epidemiology. 2006;163(6):493–501. doi: 10.1093/aje/kwj069 16443796 10.1093/aje/kwj069

[107] Kjaergard LL, Villumsen J, Gluud C. Reported methodologic quality and discrepancies between large and small randomized trials in meta-analyses. Annals of Internal Medicine. 2001;135(11):982–9. 11730399 10.7326/0003-4819-135-11-200112040-00010

[108] Lundh A, Sismondo S, Lexchin J, Busuioc OA, Bero L. Industry sponsorship and research outcome. Cochrane Database of Systematic Reviews. 2012;12:Mr000033 doi: 10.1002/14651858.MR000033.pub2 23235689 10.1002/14651858.MR000033.pub2

[109] Moher D, Pham B, Jones A, Cook DJ, Jadad AR, Moher M, et al Does quality of reports of randomised trials affect estimates of intervention efficacy reported in meta-analyses? Lancet. 1998;352(9128):609–13. doi: 10.1016/S0140-6736(98)01085-X 9746022 10.1016/S0140-6736(98)01085-X

[110] Schulz KF, Chalmers I, Hayes RJ, Altman DG. Empirical evidence of bias. Dimensions of methodological quality associated with estimates of treatment effects in controlled trials. JAMA. 1995;273(5):408–12. 7823387 10.1001/jama.273.5.408

[111] Wood L, Egger M, Gluud LL, Schulz KF, Juni P, Altman DG, et al Empirical evidence of bias in treatment effect estimates in controlled trials with different interventions and outcomes: meta-epidemiological study. BMJ. 2008;336(7644):601–5. doi: 10.1136/bmj.39465.451748.AD 18316340 10.1136/bmj.39465.451748.AD PMC2267990

[112] Savovic J, Jones HE, Altman DG, Harris RJ, Juni P, Pildal J, et al Influence of reported study design characteristics on intervention effect estimates from randomized, controlled trials. Annals of Internal Medicine. 2012;157(6):429–38. doi: 10.7326/0003-4819-157-6-201209180-00537 22945832 10.7326/0003-4819-157-6-201209180-00537

[113] Garattini S, Jakobsen JC, Wetterslev J, Bertele V, Banzi R, Rath A, et al Evidence-based clinical practice: Overview of threats to the validity of evidence and how to minimise them. European Journal of Internal Medicine. 2016;32:13–21. doi: 10.1016/j.ejim.2016.03.020 27160381 10.1016/j.ejim.2016.03.020

[114] Al-Khatib SM, Allen LaPointe NM, Chatterjee R, Crowley MJ, Dupre ME, Kong DF, et al Rate- and rhythm-control therapies in patients with atrial fibrillation: a systematic review. Annals of Internal Medicine. 2014;160(11):760–73. doi: 10.7326/M13-1467 24887617 10.7326/M13-1467

[115] Caldeira D, David C, Sampaio C. Rate versus rhythm control in atrial fibrillation and clinical outcomes: updated systematic review and meta-analysis of randomized controlled trials. Archives of Cardiovascular Diseases. 2012;105(4):226–38. doi: 10.1016/j.acvd.2011.11.005 22633297 10.1016/j.acvd.2011.11.005

[116] Chatterjee S, Sardar P, Lichstein E, Mukherjee D, Aikat S. Pharmacologic rate versus rhythm-control strategies in atrial fibrillation: an updated comprehensive review and meta-analysis. Pacing and Clinical Electrophysiology. 2013;36(1):122–33. doi: 10.1111/j.1540-8159.2012.03513.x 22978656 10.1111/j.1540-8159.2012.03513.x

[117] Cordina J, Mead G. Pharmacological cardioversion for atrial fibrillation and flutter. Cochrane Database of Systematic Reviews. 2005(2):Cd003713 doi: 10.1002/14651858.CD003713.pub2 15846675 10.1002/14651858.CD003713.pub2

[118] de Denus S, Sanoski CA, Carlsson J, Opolski G, Spinler SA. Rate vs rhythm control in patients with atrial fibrillation: a meta-analysis. Archives of Internal Medicine. 2005;165(3):258–62. doi: 10.1001/archinte.165.3.258 15710787 10.1001/archinte.165.3.258

[119] Kumana CR, Cheung BM, Cheung GT, Ovedal T, Pederson B, Lauder IJ. Rhythm vs. rate control of atrial fibrillation meta-analysed by number needed to treat. British Journal of Clinical Pharmacology. 2005;60(4):347–54. doi: 10.1111/j.1365-2125.2005.02449.x 16187966 10.1111/j.1365-2125.2005.02449.x PMC1884833

[120] Testa L, Biondi-Zoccai GG, Dello Russo A, Bellocci F, Andreotti F, Crea F. Rate-control vs. rhythm-control in patients with atrial fibrillation: a meta-analysis. European Heart Journal. 2005;26(19):2000–6. doi: 10.1093/eurheartj/ehi306 15872032 10.1093/eurheartj/ehi306

[121] Chen S, Dong Y, Fan J, Yin Y. Rate vs. rhythm control in patients with atrial fibrillation—an updated meta-analysis of 10 randomized controlled trials. International Journal of Cardiology. 2011;153(1):96–8. doi: 10.1016/j.ijcard.2011.09.009 21963208 10.1016/j.ijcard.2011.09.009

[122] Noheria A, Shrader P, Piccini JP, Fonarow GC, Kowey PR, Mahaffey KW, et al Rhythm control versus rate control and clinical outcomes in patients with atrial fibrillation. JACC: Clinical Electrophysiology. 2016;2(2):221–9. 10.1016/j.jacep.2015.11.001 29766874

[123] Ionescu-Ittu R, Abrahamowicz M, Jackevicius CA, Essebag V, Eisenberg MJ, Wynant W, et al Comparative effectiveness of rhythm control vs rate control drug treatment effect on mortality in patients with atrial fibrillation. Archives of Internal Medicine. 2012;172(13):997–1004. doi: 10.1001/archinternmed.2012.2266 22664954 10.1001/archinternmed.2012.2266

[124] Camm AJ, Breithardt G, Crijns H, Dorian P, Kowey P, Le Heuzey JY, et al Real-life observations of clinical outcomes with rhythm- and rate-control therapies for atrial fibrillation RECORDAF (Registry on Cardiac Rhythm Disorders Assessing the Control of Atrial Fibrillation). Journal of the American College of Cardiology. 2011;58(5):493–501. doi: 10.1016/j.jacc.2011.03.034 21777747 10.1016/j.jacc.2011.03.034

[125] Purmah Y, Proietti M, Laroche C, Mazurek M, Tahmatzidis D, Boriani G, et al Rate vs. rhythm control and adverse outcomes among European patients with atrial fibrillation. Europace. 2017. 10.1093/europace/euw421 28160483

[126] Deeks JJ, Dinnes J, D'Amico R, Sowden AJ, Sakarovitch C, Song F, et al Evaluating non-randomised intervention studies. Health Technology Assessment. 2003;7(27):iii–x, 1–173. 14499048 10.3310/hta7270

[127] Macle L, Andrade J, Atzema C, Bell AD, Cairns JA, Connolly S, et al. Supplementary material: complete guidelines listing—2010 to 2016. Available from https://www.ccs.ca/images/Guidelines/Guidelines_POS_Library/2016%20AF%20Update_Supplement_Final_PDF.pdf

[128] Van Gelder IC, Rienstra M, Crijns HJ, Olshansky B. Rate control in atrial fibrillation. Lancet. 2016;388(10046):818–28. doi: 10.1016/S0140-6736(16)31258-2 27560277 10.1016/S0140-6736(16)31258-2

[129] Kirchhof P. The future of atrial fibrillation management: integrated care and stratified therapy. Lancet. 2017. 10.1016/S0140-6736(17)31072-3 28460828

[130] Chan AW, Tetzlaff JM, Altman DG, Laupacis A, Gotzsche PC, Krleza-Jeric K, et al SPIRIT 2013 statement: defining standard protocol items for clinical trials. Annals of Internal Medicine. 2013;158(3):200–7. doi: 10.7326/0003-4819-158-3-201302050-00583 23295957 10.7326/0003-4819-158-3-201302050-00583 PMC5114123

[131] Schulz KF, Altman DG, Moher D. CONSORT 2010 statement: updated guidelines for reporting parallel group randomised trials. PLoS Medicine. 2010;7(3):e1000251 doi: 10.1371/journal.pmed.1000251 20352064 10.1371/journal.pmed.1000251 PMC2844794

