# Supplementary material for: The effects of rhythm control strategies versus rate control strategies for atrial fibrillation and atrial flutter: A systematic review with meta-analysis and Trial Sequential Analysis
Source: PLoS One. 2017 Oct 26;12(10):e0186856. doi: 10.1371/journal.pone.0186856 (PMC5658096; doi:10.1371/journal.pone.0186856)
Supplement: S2 Text — (PDF) [file pone.0186856.s002.pdf]

Sethi et al. Systematic Reviews (2017) 6:47
DOI 10.1186/s13643-017-0449-z

Systematic Reviews

PROTOCOL Open Access

The effects of rhythm control strategies versus rate control strategies for atrial fibrillation and atrial flutter: a protocol for a systematic review with meta-analysis and Trial Sequential Analysis

Naqash J. Sethi[1*], Sanam Safi[1], Emil E. Nielsen[1], Joshua Feinberg[1], Christian Gluud[1,2] and Janus C. Jakobsen[1,2,3]

Abstract

Background: Atrial fibrillation is the most common arrhythmia of the heart with a prevalence of approximately 2% in the western world. Atrial flutter, another arrhythmia, occurs less often with an incidence of approximately 200,000 new patients per year in the USA. Patients with atrial fibrillation and atrial flutter have an increased risk of death and morbidities. The management of atrial fibrillation and atrial flutter is often based on interventions aiming at either a rhythm control strategy or a rate control strategy. The evidence on the comparable effects of these strategies is unclear. This protocol for a systematic review aims at identifying the best overall treatment strategy for atrial fibrillation and atrial flutter.

Methods: This protocol for a systematic review was performed following the recommendations of the Cochrane Collaboration and the eight-step assessment procedure suggested by Jakobsen and colleagues. We plan to include all relevant randomised clinical trials assessing the effects of any rhythm control strategy versus any rate control strategy. We plan to search the Cochrane Central Register of Controlled Trials (CENTRAL), MEDLINE, EMBASE, LILACS, Science Citation Index Expanded on Web of Science, and BIOSIS to identify relevant trials. Any eligible trial will be assessed and classified as either high risk of bias or low risk of bias, and our conclusions will be based on trials with low risk of bias. The analyses of the extracted data will be performed using Review Manager 5 and Trial Sequential Analysis. For both our primary and secondary outcomes, we will create a 'Summary of Findings' table and use GRADE assessment to assess the quality of the evidence.

Discussion: The results of this systematic review have the potential to benefit thousands of patients worldwide as well as healthcare systems and healthcare economy.

Systematic review registration: PROSPERO CRD42016051433

Keywords: Atrial fibrillation, Atrial flutter, Rhythm control, Rate control, Systematic review, Meta-analysis, Trial Sequential Analysis

* Correspondence: hvg658@alumni.ku.dk
[1]Copenhagen Trial Unit, Centre for Clinical Intervention Research, Department 7812, Rigshospitalet, Copenhagen University Hospital, Copenhagen, Denmark
Full list of author information is available at the end of the article

BioMed Central

© The Author(s). 2017 **Open Access** This article is distributed under the terms of the Creative Commons Attribution 4.0 International License (http://creativecommons.org/licenses/by/4.0/), which permits unrestricted use, distribution, and reproduction in any medium, provided you give appropriate credit to the original author(s) and the source, provide a link to the Creative Commons license, and indicate if changes were made. The Creative Commons Public Domain Dedication waiver (http://creativecommons.org/publicdomain/zero/1.0/) applies to the data made available in this article, unless otherwise stated.

Background

Atrial fibrillation is the most common arrhythmia of the heart with a prevalence of approximately 2% in the western world [1, 2]. Atrial flutter, another arrhythmia, occurs less often with an incidence of approximately 200,000 new patients per year in the USA [3]. The prevalence of both atrial fibrillation and atrial flutter are increasing possibly because of a greater life expectancy in the general population, an increased prevalence of risk factors for atrial fibrillation and atrial flutter, and an improved ability to suspect and diagnose the arrhythmias [1, 4, 5]. Atrial fibrillation and atrial flutter are associated with an increased risk of death and morbidities [6–12]. The risks of both cerebral stroke and heart failure are increased nearly fivefold in patients with atrial fibrillation and atrial flutter, and an estimated 20% of every stroke may be due to atrial fibrillation [6–11]. Atrial fibrillation and atrial flutter also have a significant impact on healthcare costs and account for approximately 1% of the National Health Service budget in the UK and approximately 26 billion dollars of annual expenses in the USA [13, 14].

Definition and classification

The atriums of the heart receive blood returning from the body and pump it further ahead to the ventricles. Atrial fibrillation and atrial flutter are defined as abnormal heart rhythms that arise from improper electrical activity of the heart which lead to ineffective mechanical contraction [15–17]. The ineffective mechanical contraction stresses the muscle cells of the heart which over time may cause heart failure [18, 19]. Persistent rapid rates can also cause or worsen a tachycardia-mediated cardiomyopathy [20].

Atrial fibrillation and atrial flutter can be asymptomatic or lead to symptoms such as palpitations, dyspnoea, and dizziness [21]. Atrial fibrillation may be diagnosed using an electrocardiogram as (1) irregular R-R intervals (when atrioventricular conduction is present), (2) absence of distinct repeating *P*-waves, and/or (3) irregular atrial activity [16, 17]. Atrial flutter may be diagnosed using an electrocardiogram as characteristic flutter waves (*F*-waves) at a regular atrial rate of 250 to 350 beats per minute. The flutter waves may resemble *P*-waves or have a 'saw-tooth' shape [22].

Atrial fibrillation may either be non-valvular or valvular, where the latter form is characterised by rheumatic mitral stenosis, mechanical heart valve, tissue heart valve, or mitral valve repair [1]. However, the definition of the terms non-valvular and valvular lacks consistency in both trials and guidelines [23, 24]. A paper has proposed a new term 'mechanical and rheumatic mitral valvular atrial fibrillation', as they report that only mechanical valves and mitral stenosis have special needs in regard to antithrombotic treatment [24].

The development of atrial fibrillation is associated with various risk factors, e.g. ageing, obesity, smoking, hypertension, diabetes, and other cardiac diseases (valvular or other structural heart diseases) [17, 25]. The development of atrial flutter is presumably associated with prolonged PR interval and some of the same risk factors as atrial fibrillation [8]. However, it has not been demonstrated that atrial flutter is associated with either obesity, diabetes, hypertension, or valvular heart disease [8]. Both atrial fibrillation and atrial flutter may also occur in patients with no risk factors (so called lone atrial fibrillation or lone atrial flutter) [3].

Based on the duration of the arrhythmia, atrial fibrillation may be divided into five different forms [15–17]:

- Recent-onset atrial fibrillation
- Paroxysmal atrial fibrillation
- Persistent atrial fibrillation
- Long-standing persistent atrial fibrillation
- Permanent atrial fibrillation

Based on the re-entrant circuit, atrial flutter may be divided into two different forms:

- Typical atrial flutter is a macro-reentrant atrial tachycardia that can be subdivided based on the rotation of the circuit to counterclockwise atrial flutter (90% of patients) or clockwise atrial flutter (10% of patients) [26].
- Atypical atrial flutter is defined as any atrial tachycardia with an ECG pattern of continuous undulation of the atrial complex, different from typical atrial flutter, at a rate of ≥240 beats per minute [26].

Pathophysiology

The pathogenesis of atrial fibrillation is thought to be an interaction between a trigger for initiation and an abnormal tissue substrate for maintenance [25].

The trigger for initiation is often a rapidly firing focus most often located in the left atrium and the proximal parts of the pulmonary veins [27]. The abnormal tissue substrate for maintenance is often a result of an underlying heart disease like coronary heart disease, valvular heart disease, cardiomyopathies, or heart failure [16]. The pathogenesis of the abnormal tissue substrate is induced by inflammation [28], fibrosis [29], or hypertrophy [30].

Electric remodelling, such as refractory period shortening, occurs after a period of continuous atrial fibrillation that further facilitate atrial fibrillation, i.e. atrial fibrillation leads to atrial fibrillation [30, 31]. Nevertheless, the electric remodelling is often reversible if sinus

rhythm is restored, though it can become permanent if atrial fibrillation persists [31].

Atrial flutter is classified as a macro-reentrant tachycardia. The macro-reentrant tachycardia occurs when an electrical impulse recurrently moves in a self-perpetuating circuit within the heart, rather than moving from one end of the heart to the other and terminating [26].

Antithrombotic treatment

As mentioned in the 'Background' section, the risk of stroke is increased nearly fivefold in patients with atrial fibrillation and atrial flutter [10]. Antithrombotic treatment is necessary to reduce the risk of stroke in high-risk patients with atrial fibrillation and atrial flutter, regardless of the management strategy [16]. The risk of stroke in patients with atrial fibrillation and atrial flutter can be estimated by the CHA2DS2-VASc score [32], while the risk of bleeding can be estimated by the HAS-BLED score [33]. Combined, these may help the physician determine the patient's need for antithrombotic treatment [16].

Antithrombotic drugs aim at reducing the formation of thrombi by affecting different clotting processes. Depending on the mechanism, the drugs are divided into either anticoagulants or antiplatelet drugs. The classification, mechanism, and examples of anticoagulants and antiplatelet drugs are summarised in Table 1.

The comparative efficacy and safety between anticoagulants and antiplatelet drugs has been assessed. Two systematic reviews have shown that both warfarin and apixaban are superior to antiplatelet drugs for preventing stroke, with a similar rate of major bleeding and intracranial haemorrhage [34, 35].

Table 1 The classification, mechanism, and examples of anticoagulants and antiplatelet drugs

Class	Mechanism	Examples
Anticoagulants	Affect the coagulation cascade to prevent blood coagulation [112]	Vitamin K-dependent antagonists<br>•Warfarin<br>Non-vitamin K-dependent antagonists<br>•Dabigatran<br>•Rivaroxaban<br>•Apixaban<br>•Edoxaban<br>Heparin<br>•Unfractionated heparin<br>•Low-molecular-weight heparin
Antiplatelet drugs	Theoretically affect the migration and aggregation of platelets, consequently aiming at inhibiting thrombus formation [112]	•Aspirin<br>•Clopidogrel<br>•Prasugrel<br>•Ticagrelor<br>•Cangrelor<br>•Abciximab<br>•Eptifibatid<br>•Dipyridamole

The comparative efficacy and safety between warfarin and non-vitamin K-dependent antagonist has been assessed. Ruff et al. showed in a systematic review that the non-vitamin K-dependent antagonists compared with warfarin significantly reduced the risk of all-cause mortality by 10%, stroke by 19%, and intracranial haemorrhage by 52%. However, the risk of gastrointestinal bleeding was increased by 25% by the non-vitamin K-dependent antagonists [36].

Description of the interventions

Two different overall intervention strategies may be used for atrial fibrillation and atrial flutter—a rhythm control strategy and a rate control strategy [16]. The overall aims of a rhythm control strategy and a rate control strategy differ. A rhythm control strategy aims at obtaining and maintaining sinus rhythm, while a rate control strategy is an overall term for a strategy where the short- and long-term aim is to lower the ventricular frequency [25]. Patients that receive rhythm control will often need some kind of rate control until they have obtained sinus rhythm.

The interventions used for both rhythm- and rate control strategies encompass both drugs and ablation. In addition, electrical cardioversion is also used for rhythm control.

The drugs used in both a rhythm control strategy and a rate control strategy are classified according to two different classifications: the Vaughan Williams classification and the Sicilian Gambit classification.

- The Vaughan Williams classification classifies the drugs in five different classes according to their general effect. Class I and III drugs are mainly used for a rhythm control strategy; class II and IV drugs are mainly used for a rate control strategy; and class V drugs are used for both strategies [37]. The Vaughan Williams classification is summarised in Table 2.
- The Sicilian Gambit classification places a greater approach on the underlying mechanism of the drugs and classifies each drug according to the effects on bio-cellular channels, receptors, and pumps. We will not describe this classification in detail but refer to the work by the European Society of Cardiology [38].

We will in this systematic review use the Vaughan Williams classification which is the most commonly used classification.

Rhythm control strategies

A rhythm control strategy typically uses medical rhythm control or electrical cardioversion as the main rhythm

Table 2 The Vaughan Williams classification

Class	Mechanism	Examples
Ia	Na^+ channel block (moderate)	•Quinidine<br>•Ajmaline<br>•Procainamide<br>•Disopyramide
Ib	Na^+ channel block (weak)	•Lidocaine<br>•Phenytoin<br>•Mexiletine<br>•Tocainide
Ic	Na^+ channel block (strong)	•Flecainide<br>•Propafenone<br>•Encainide<br>•Moricizine
II	Beta-blocker	•Propranolol<br>•Carvedilol<br>•Esmolol<br>•Timolol<br>•Metoprolol<br>•Atenolol<br>•Bisoprolol<br>•Nebivolol
III	K^+ channel blocker	•Amiodarone<br>•Dronedarone<br>•Sotalol<br>•Ibutilide<br>•Dofetilide<br>•Vernakalant
IV	Ca^{2+} channel blocker	•Verapamil<br>•Diltiazem
V	Variable	•Adenosine<br>•Digoxin<br>•Magnesium sulphate

control intervention. If they do not work, catheter ablation or surgical ablation may be considered [39].

Medical rhythm control

Medical rhythm control involves antiarrhythmic drugs and is used for either (1) cardioversion of atrial fibrillation or atrial flutter to sinus rhythm or (2) maintenance of sinus rhythm [16].

The main drugs used for medical cardioversion of atrial fibrillation and atrial flutter are flecainide (class Ic), propafenone (class Ic), dofetilide (class III), and amiodarone (class III) [16]. A systematic review showed that intravenous vernakalant (class III), intravenous propafenone (class Ic), and both oral and intravenous flecainide (class Ic) seemed to be significantly more effective than placebo at restoring sinus rhythm within 2 h of administration in recent-onset atrial fibrillation [40]. When they analysed for successful cardioversion within 8–24 h, oral amiodarone (class III), oral flecainide (Ic), and both oral and intravenous propafenone (class Ic) seemed to be significantly more effective than placebo [40].

The main drugs used for maintenance of sinus rhythm are amiodarone (class III), dofetilide (class III), dronedarone (class III), flecainide (class Ic), propafenone (class Ic), beta blockers (class II), and sotalol (class III) [16]. Lafuente-Lafuente et al. showed in a Cochrane review that drugs belonging to class Ia (disopyramide and quinidine), class Ic (flecainide and propafenone), class II (metoprolol), and class III (amiodarone, dofetilide, dronedarone, and sotalol) were moderately effective in maintaining sinus rhythm compared with patients not receiving antiarrhythmic drugs (56). Nonetheless, all drugs were associated with adverse events, including proarrhythmia (new or more frequent occurrence of pre-existing arrhythmias) [41]. Treatment with quinidine (class Ia), disopyramide (class Ia), or sotalol (class III) compared with not receiving antiarrhythmic drugs was associated with higher risks of all-cause mortality and serious adverse events [41]. In regard to class Ic drugs (flecainide and propafenone), no increased risk of mortality was found. However, as the data obtained on mortality with flecainide (class Ic) and propafenone (class Ic) seemed sparse, the authors concluded that the result was uncertain [41]. Guidelines recommend that flecainide (class Ic) and propafenone (class Ic) should only be used in patients without ischaemic heart disease or heart failure [16, 42]. This is based on a randomised trial from 1989, the Cardiac Arrhythmia Suppression Trial (CAST), that compared antiarrhythmic drug therapy with placebo in patients with asymptomatic or mildly symptomatic ventricular arrhythmia (six or more ventricular premature beats per hour) after myocardial infarction [43]. The trial showed an increased risk of mortality and sudden cardiac death caused by ventricular arrhythmias in the patients receiving encainide (class Ic) and flecainide (class Ic) compared with placebo [43].

In several systematic reviews, amiodarone (class III) was shown to be better than class I (flecainide, propafenone, disopyramide, and quinidine) and other class III drugs (sotalol, dofetilide, and dronedarone) at maintaining sinus rhythm [41, 44–46]. However, in one systematic review, amiodarone (class III) was shown to induce a higher number of adverse events [46].

Electrical cardioversion rhythm control

Electrical cardioversion is a non-invasive procedure that uses electrical shock to convert atrial fibrillation and atrial flutter (or other arrhythmias) into sinus rhythm [16]. Current evidence supports the use of electrical cardioversion with biphasic waveforms with an intensity of 200 J for atrial fibrillation, as the proportion of success is 91 to 94% [47–49]. The monophasic waveform requires intensity up to 360 J to work, and the proportion of success is 79 to 85% [47–49]. For atrial flutter, evidence supports the use of biphasic waveforms with an intensity of 50 to 100 J [50, 51]. It has been shown that longer duration of atrial fibrillation was inversely associated with cardioversion proportions [52, 53]. To enhance

the effectiveness of the procedure, antiarrhythmic drugs, such as amiodarone (class III), flecainide (class Ic), ibutilide (class Ic), propafenone (class Ic), and sotalol (class III), can be used [54].

Ablation procedures for rhythm control

Two different ablation procedures may be used for rhythm control in patients with atrial fibrillation and atrial flutter—catheter ablation or surgical ablation. Both procedures do not involve controlling the heart rate but aim at obtaining sinus rhythm.

Catheter ablation may be indicated in patients with paroxysmal, persistent, or long-standing persistent atrial fibrillation or atrial flutter that is refractory or intolerant to medical rhythm control [15]. Catheter ablation of atrial fibrillation is mostly performed in the left atrium, usually entering via the vena femoralis, vena jugularis interna, or vena subclavia. In the left atrium, a series of lesions are created, and the lesions are thought either to eliminate possible triggers originating from the pulmonary veins or to modify the substrate that maintains the atrial fibrillation [15]. Catheter ablation may also be indicated in patients with typical atrial flutter where the lesions caused by catheter ablation are thought to interrupt the macro-reentrant circuit maintaining the atrial flutter [55]. The main technique for either atrial fibrillation or atrial flutter is radiofrequency ablation that achieves myocardial necrosis through tissue heating [15]. In an observational study, the risk of major complications (e.g. death, tamponade, total femoral pseudoaneurysm, or transient ischemic attack) after catheter ablation was found to be 4.5% based on 20,825 procedures [56].

Surgical ablation is performed doing a Cox-Maze procedure by open-heart surgery or a less-invasive right mini thoracotomy [57]. A Cox-Maze procedure is done by creating a number of surgical lesions in the left and right atrium in order to form scar tissue. The scar tissue inhibits the conduction of electricity, consequently disrupting the abnormal electrical impulses [58]. The procedure is most often done concomitantly with other cardiac surgery than as a lone procedure [57]. A randomised clinical trial compared surgical ablation with no surgical ablation during mitral-valve surgery and showed significantly higher conversion rates in the surgical ablation group [59]. The risk of mortality was similar to both groups. However, there was a significantly higher rate of permanent pacemaker implantation in the surgical ablation group than in the no surgical ablation group [59].

Rate control strategies

Medical rate control

The drugs used for rate control in atrial fibrillation and atrial flutter are mainly beta blockers (class II), non-dihydropyridine calcium channel blockers (class IV), and digoxin (class V) [16, 22]. All three types of drugs work by lowering the heart rate which might consequently prevent excessive tachycardia and limit symptoms. Lowering the heart rate might theoretically prevent the development of heart failure and tachycardia-mediated cardiomyopathy [18, 20, 60]. An observational study compared rate control with no rate control and showed lower risk of mortality in the patients receiving beta blockers (class II) or non-dihydropyridine calcium channel blockers (class IV). In contrast, the patients receiving digoxin (class V) seemed to have a higher risk of mortality [61]. A systematic review compared rate control interventions with placebo and showed that selective beta blockers (class II), verapamil (class IV), diltiazem (class IV), and partial digoxin (class V) were better than placebo at lowering the heart rate [62]. According to guidelines, beta blockers (class II) or non-dihydropyridine calcium channel blockers (class IV) are first-line therapy, while digoxin (class V) may be combined with one of them if they alone are insufficient to control the heart rate [16]. The Atrial Fibrillation Follow-up Investigation of Rhythm Management (AFFIRM) trial showed that beta-blockers (class II) (with or without digoxin (class V)) achieved rate control (rest ≤80 beats/min) in 70% of the patients compared with 54% for non-dihydropyridine calcium channel blockers (class IV) (with or without digoxin (class V)) and 58% for digoxin (class V) (used without beta-blockers (class II) or non-dihydropyridine calcium channel blockers (class IV)) [63]. Amiodarone (class III) may also control the heart rate, as it exhibits beta and calcium channel blockade in addition to its antiarrhythmic activity. However, amiodarone (class III) has extensive non-cardiac adverse events and is only used if other rate control drugs are not effective enough, not well tolerated, or contraindicated [16]. According to guidelines, physicians should consider the patient's degree of symptoms, haemodynamic status, presence or absence of heart failure, and comorbidities when choosing which rate control intervention to use [16].

During recent years, observational studies have compared digoxin versus no digoxin in patients with atrial fibrillation or atrial flutter and showed conflicting results [64–68]. Some studies have shown that digoxin seemed to increase the risk of all-cause mortality regardless of concomitant heart failure [64, 65], while others did not show any difference between the compared groups [66–68]. Nonetheless, guidelines recommend using digoxin as the primary drug for rate control in patients with atrial fibrillation or atrial flutter who have concomitant heart failure and reduced ejection fraction. Digoxin is also recommended for acute rate control in patients with preserved ejection fraction [16, 42, 69].

Ablation procedures for rate control

Atrioventricular node ablation procedure is a rare but highly effective procedure that controls the ventricular

heart rate with the help of a pacemaker [70]. The procedure is done by ablation of the atrioventricular node via assess from vena femoralis dexter, consequently cancelling all electrical impulses from the atriums to the ventricles. Hence, the procedure induces complete atrioventricular block and permanent pacing is necessary [71]. The procedure does not involve rhythm control, and atrial fibrillation or atrial flutter is still present after the procedure. The atrioventricular node ablation procedure primarily benefits patients with atrial fibrillation or atrial flutter who have symptoms refractory to medical rate control [70].

Why is it important to do this review?

Atrial fibrillation and atrial flutter are the most common arrhythmias of the heart and are associated with an increased risk of death and morbidities [1–3, 6–12]. The treatment of atrial fibrillation and atrial flutter is based on two overall treatment strategies—a rhythm control strategy and a rate control strategy [16].

Several meta-analyses of randomised trials have compared rhythm control strategies with rate control strategies in patients with atrial fibrillation or atrial flutter [72–75]. Cordina et al. from 2005 included 2 trials with 4312 participants [73]; Caldeira et al. from 2012 included 8 trials with 7499 participants [75]; Chatterjee et al. from 2013 included 10 trials with 7867 participants [74]; and Al-Khatib et al. from 2014 included 16 trials with 7608 participants [72]. None of the reviews showed any difference in effects of any of the strategies on all-cause death and other patient-centred clinical outcomes. However, Chatterjee et al. showed lower risk of all-cause mortality in patients younger than 65 years in the rhythm control group compared with the rate control group [74]. Cordina et al. showed significantly higher rates of adverse events in the rhythm control group compared with the rate control group [73].

No former review comparing rhythm control strategies with rate control strategies has taken into account both risks of systematic errors and risks of random errors (Cochrane methodology, Trial Sequential Analysis, and the Grades of Recommendation, Assessment, Development, and Evaluation (GRADE) assessment) [76–79]. Therefore, it is still unclear whether a rhythm control strategy or a rate control strategy is the best treatment strategy in patients with atrial fibrillation and atrial flutter. In the present systematic review, we will collect and present current evidence of rhythm control versus rate control for atrial fibrillation and atrial flutter.

Objective

The objective of the study is to assess the beneficial and harmful effects of rhythm control strategies versus rate control strategies for atrial fibrillation and atrial flutter.

Methods

This systematic review protocol has been developed based on Preferred Reporting Items for Systematic Reviews and Meta-Analysis Protocols (PRISMA-P) guidelines for reporting systematic reviews evaluating healthcare interventions [80, 81]. A PRISMA-P checklist file is attached (Additional file 1).

Criteria for considering studies for this review

Types of studies

Randomised clinical trials irrespective of trial design, setting, publication status, publication year, and language. We will not include quasi-randomised trials and observational studies for the assessments of harms. We are aware that this is a limitation of our review.

Types of participants

Patients with atrial fibrillation or atrial flutter. We will accept the definitions used by the trialists. Patients will be included irrespective of age, sex, and comorbidities.

Types of interventions

Rhythm control group: we will accept any type of rhythm control strategy, i.e. any intervention where the overall aim is to convert the atrial fibrillation or atrial flutter to sinus rhythm. Treatment elements of the rhythm control strategy could for example be flecainide, propafenone, dofetilide, amiodarone, dronedarone, sotalol, or electrical cardioversion (all irrespective of dose, rout of administration, and duration). We will accept if the rhythm control strategy also includes rate control interventions as part of treatment, but the overall aim (short or long term) has to be to obtain sinus rhythm.

Rate control group: we will accept any type of rate control strategy, i.e. any intervention where the overall aim is to control the heart rate and the focus is not to convert the atrial fibrillation or atrial flutter to sinus rhythm. Treatment elements of the rate control strategy could for example be beta blockers, non-dihydropyridine calcium channel blockers, digoxin, or amiodarone (all irrespective of dose, route of administration, and duration).

We will accept any type of co-intervention when such co-intervention is intended to be delivered similar to the rhythm control group and the rate control group.

Types of outcome measures

We will for all outcomes use the trial results reported at maximal follow-up. However, if the trialists report results at multiple time points, we will primarily use the results reported at the time point closest to 24 months.

Primary outcomes

1. All-cause mortality.

2. Serious adverse events. We will define a serious adverse event as any untoward medical occurrence that resulted in death, was life-threatening, required hospitalisation or prolongation of existing hospitalisation, and resulted in persistent or significant disability or jeopardised the patient [82].
3. Quality of life measured on any valid scale.

Secondary outcomes

1. Stroke (as defined by the trialists)
2. Ejection fraction (continuous outcome)

All outcomes, except quality of life and ejection fraction, will be analysed as proportions of participants in each group.

Search methods for identification of studies

Electronic searches

We will search the Cochrane Central Register of Controlled Trials (CENTRAL), Medical Literature Analysis and Retrieval System Online (MEDLINE), Excerpta Medica database (EMBASE), Latin American and Caribbean Health Sciences Literature (LILACS), Science Citation Index Expanded on Web of Science, and BIOSIS in order to identify relevant trials. The preliminary search strategy for MEDLINE (Ovid) is given in Additional file 2.

We will search all databases from their inception to the present.

Searching other resources

The reference lists of relevant publications will be checked for any unidentified randomised trials. We will contact authors of included studies, and major pharmaceutical companies, by email asking for unpublished randomised trials. Further, we will search for ongoing trials on:

- ClinicalTrials.gov (www.clinicaltrials.gov)
- Google Scholar (https://scholar.google.dk/)
- The Turning Research into Practice (TRIP) Database (https://www.tripdatabase.com/)
- European Medicines Agency (EMA) (http://www.ema.europa.eu/ema/)
- United States Food and Drug Administration (FDA) (www.fda.gov)
- China Food and Drug Administration (CFDA) (http://eng.sfda.gov.cn/WS03/CL0755/)
- Medicines and Healthcare products Regulatory Agency (https://www.gov.uk/government/organisations/medicines-and-healthcare-products-regulatory-agency)
- The World Health Organization (WHO) International Clinical Trials Registry Platform (ICTRP) search portal (http://apps.who.int/trialsearch/)

Additionally, we will handsearch conference abstracts from cardiology conferences for relevant trials.

We will also consider relevant for the review unpublished and grey literature trials if we identify these.

Data collection and analysis

We will perform the review following the recommendations of the Cochrane Collaboration [79]. The analyses will be performed using Review Manager 5 [83] and Trial Sequential Analysis [84]. In case of Review Manager statistical software not being sufficient, we will use STATA 14 [85].

Selection of studies

Two authors (NJS and SS) will independently screen titles and abstracts. We will retrieve all relevant full-text study reports/publications, and two review authors (NJS and SS) will independently screen the full text and identify and record reasons for exclusion of the ineligible studies. We will resolve any disagreement through discussion or, if required, we will consult a third person (JCJ). Trial selection will be displayed in an adapted flow diagram as per the Preferred Reporting Items for Systematic Reviews and Meta-Analyses (PRISMA) statement [86].

Data extraction and management

Four authors (NJS, SS, EEN, and JF) will in pairs extract data independently from included trials. Disagreements will be resolved by discussion with a fifth author (JCJ). We will assess duplicate publications and companion papers of a trial together to evaluate all available data simultaneously (maximise data extraction, correct bias assessment). We will contact the trial authors by email to specify any additional data, which may not have been reported sufficiently or at all in the publication.

Trial characteristics

Bias risk components (as defined below); trial design (parallel, factorial, or crossover); number of intervention arms; length of follow-up; estimation of sample size; inclusion and exclusion criteria.

Participant characteristics and diagnosis

Number of randomised participants; number of analysed participants; number of participants lost to follow-up/withdrawals/crossover; compliance with medication; age range (mean or median) and sex ratio; type of arrhythmia (atrial fibrillation or atrial flutter); baseline numbers of cardiovascular risk factors (i.e. diabetes mellitus, hypertension, hyperlipidaemia, or smoking); baseline number of

participants with heart failure; baseline number of participants with valvular heart disease; baseline number of participants with previous myocardial infarction; baseline number of participants with previous revascularisation; and baseline number of participants with previous angina.

We will additionally report the proportion of participants in the compared groups who receive electrical cardioversion, atrioventricular node ablation, catheter ablation, and surgical ablation.

Rhythm control strategy characteristics

Type of rhythm control intervention, type of rate control intervention, dose of intervention, duration of therapy, and mode of administration.

Rate control strategy characteristics

Type of rate control intervention, dose of intervention, duration of therapy, and mode of administration.

Co-intervention characteristics

Type of co-intervention; dose of co-intervention; duration of co-intervention; and mode of administration.

Outcomes

All outcomes listed above will be extracted from each randomised clinical trial, and we will identify if outcomes are incomplete or selectively reported according to the criteria described later in 'incomplete outcome data' bias domain and 'selective outcome reporting' bias domain.

Notes

Funding of the trial and notable conflicts of interest of trial authors will be extracted, if available.

We will note in the 'Characteristics of included studies' table if outcome data were not reported in a usable way. Two review authors (NJS and SS) will independently transfer data into the Review Manager file [83]. Disagreements will be resolved through discussion or, if required, we will consult with a third author (JCJ).

Assessment of risk of bias in included studies

We will use the instructions given in the Cochrane Handbook for Systematic Reviews of Interventions [79] in our evaluation of the methodology and hence the risk of bias of the included trials. We will evaluate the methodology in respect of:

- Random sequence generation
- Allocation concealment
- Blinding of participants and treatment providers
- Blinding of outcome assessment
- Incomplete outcome data
- Selective outcome reporting
- Other risks of bias
- Overall risk of bias

These components enable classification of randomised trials with low risk of bias and high risk of bias. The latter trials tend to overestimate positive intervention effects and underestimate negative effects [87–93].

We will classify the trials according to the following criteria.

Random sequence generation

- Low risk: If sequence generation was achieved using computer random number generator or a random number table. Drawing lots, tossing a coin, shuffling cards, and throwing dice were also considered adequate if performed by an independent adjudicator.
- Unclear risk: If the method of randomisation was not specified, but the trial was still presented as being randomised.
- High risk: If the allocation sequence is not randomised or only quasi-randomised. These trials will be excluded.

Allocation concealment

- Low risk: If the allocation of patients was performed by a central independent unit, on-site locked computer, identical-looking numbered sealed envelopes, drug bottles, or containers prepared by an independent pharmacist or investigator.
- Uncertain risk: If the trial was classified as randomised but the allocation concealment process was not described.
- High risk: If the allocation sequence was familiar to the investigators who assigned participants.

Blinding of participants and treatment providers

- Low risk: If the participants and the treatment providers were blinded to intervention allocation and this was described.
- Uncertain risk: If the procedure of blinding was insufficiently described.
- High risk: If blinding of participants and the treatment providers was not performed.

Blinding of outcome assessment

- Low risk of bias: If it was mentioned that outcome assessors were blinded and this was described.

- Uncertain risk of bias: If it was not mentioned if the outcome assessors in the trial were blinded or the extent of blinding was insufficiently described.
- High risk of bias: If no blinding or incomplete blinding of outcome assessors was performed.

Incomplete outcome data

- Low risk of bias: If missing data were unlikely to make treatment effects depart from plausible values. This could be either (1) there were no drop-outs or withdrawals for all outcomes or (2) the numbers and reasons for the withdrawals and drop-outs for all outcomes were clearly stated and could be described as being similar to both groups. Generally, the trial is judged as at a low risk of bias due to incomplete outcome data if drop-outs are less than 5%. However, the 5% cut-off is not definitive.
- Uncertain risk of bias: If there was insufficient information to assess whether missing data were likely to induce bias on the results.
- High risk of bias: If the results were likely to be biased due to missing data either because the pattern of drop-outs could be described as being different in the two intervention groups or the trial used improper methods in dealing with the missing data (e.g. last observation carried forward).

Selective outcome reporting

- Low risk of bias: If a protocol was published before or at the time the trial was begun and the outcomes specified in the protocol were reported on. If there is no protocol or the protocol was published after the trial has begun, reporting of all-cause mortality and serious adverse events will grant the trial a grade of low risk of bias.
- Uncertain risk of bias: If no protocol was published and the outcome all-cause mortality and serious adverse events were not reported on.
- High risk of bias: If the outcomes in the protocol were not reported on.

Other risks of bias

- Low risk of bias: If the trial appears to be free of other components (for example, academic bias or for-profit bias) that could put it at risk of bias.
- Unclear risk of bias: If the trial may or may not be free of other components that could put it at risk of bias.
- High risk of bias: If there are other factors in the trial that could put it at risk of bias (for example, authors conducted trials on the same topic, for-profit bias, etc.).

Overall risk of bias

- Low risk of bias: The trial will be classified as overall 'low risk of bias' only if all of the bias domains described in the above paragraphs are classified as 'low risk of bias'.
- High risk of bias: The trial will be classified as 'high risk of bias' if any of the bias risk domains described in the above are classified as 'unclear' or 'high risk of bias'.

We will assess the domains 'blinding of outcome assessment', 'incomplete outcome data', and 'selective outcome reporting' for each outcome result. Thus, we can assess the bias risk for each outcome assessed in addition to each trial. Our primary conclusions will be based on the results of our primary outcome results with overall low risk of bias. Both our primary and secondary conclusions will be presented in the summary of findings tables.

Differences between the protocol and the review
We will conduct the review according to this published protocol and report any deviations from it in the 'Differences between the protocol and the review' section of the systematic review.

Measures of treatment effect

Dichotomous outcomes We will calculate risk ratios (RRs) with 95% confidence interval (CI) for dichotomous outcomes, as well as the Trial Sequential Analysis-adjusted CIs (see below).

Continuous outcomes We will calculate the mean differences (MDs) and the standardised mean difference (SMD) with 95% CI for continuous outcomes, as well as the Trial Sequential Analysis-adjusted CIs (see below).

Dealing with missing data
We will, as first option, contact all trial authors to obtain any relevant missing data (i.e. for data extraction and for assessment of risk of bias, as specified above).

Dichotomous outcomes We will not impute missing values for any outcomes in our primary analysis. In two of our sensitivity analyses (see paragraph below), we will impute data.

Continuous outcomes We will primarily analyse scores assessed at single time points. If only changes from

baseline scores are reported, we will analyse the results together with follow-up scores [79]. If standard deviations (SDs) are not reported, we will calculate the SDs using trial data, if possible. We will not use intention-to-treat data if the original report did not contain such data. We will not impute missing values for any outcomes in our primary analysis. In our sensitivity analysis (see paragraph below) for continuous outcomes, we will impute data.

Assessment of heterogeneity

We will primarily investigate forest plots to visually assess any sign of heterogeneity. We will secondly assess the presence of statistical heterogeneity by chi^2 test (threshold $P < 0.10$) and measure the quantities of heterogeneity by the I^2 statistic [94, 95].

We will follow the recommendations for threshold by the *Cochrane Handbook for Systematic Reviews of Interventions* [79]:

- 0 to 40%: might not be important
- 30 to 60%: may represent moderate heterogeneity
- 50 to 90%: may represent substantial heterogeneity
- 75 to 100%: may represent considerable heterogeneity

We will investigate possible heterogeneity through subgroup analyses. Ultimately, we may decide that a meta-analysis should be avoided [79].

Assessment of reporting biases

We will use a funnel plot to assess reporting bias if ten or more trials are included. We will visually inspect funnel plots to assess the risk of bias. We are aware of the limitations of a funnel plot (i.e. a funnel plot assesses bias due to small sample size). From this information, we assess possible reporting bias. For dichotomous outcomes, we will test asymmetry with the Harbord test [96] if τ^2 is less than 0.1 and with the Rücker test if τ^2 is more than 0.1. For continuous outcomes, we will use the regression asymmetry test [97] and the adjusted rank correlation [98].

Unit of analysis issues We will only include randomised clinical trials. For trials using crossover design, only data from the first period will be included [79, 99]. There will therefore not be any unit of analysis issues. We will not include cluster randomised trials.

Data synthesis

Meta-analysis We will undertake this meta-analysis according to the recommendations stated in the *Cochrane Handbook for Systematic Reviews of Interventions* [79], Keus et al. [78], and the eight-step assessment suggested by Jakobsen et al. [76]. We will use the statistical software Review Manager 5.3 [83] provided by Cochrane to analyse data.

We will assess our intervention effects with both random-effects meta-analyses [100] and fixed-effects meta-analyses [101]. We will use the more conservative point estimate of the two [76]. The more conservative point estimate is the estimate closest to zero effect. If the two estimates are similar, we will use the estimate with the widest CI. We use three primary outcomes, and therefore, we will consider a P value of 0.025 or less as the threshold for statistical significance [76]. We use two secondary outcomes, and therefore, we will consider a P value of 0.033 or less as threshold for statistical significance [76, 102]. We will investigate possible heterogeneity through subgroup analyses. Ultimately, we may decide that a meta-analysis should be avoided [79]. We will use the eight-step procedure to assess if the thresholds for significance are crossed [76]. Our primary conclusion will be based on results with low risk of bias [76].

Where multiple trial arms are reported in a single trial, we will include only the relevant arms. If two comparisons are combined in the same meta-analysis, we will halve the control group to avoid double-counting [79].

Trials with a factorial design will be included. In case of, e.g. a 2×2 factorial designed trial, the two groups receiving rhythm control interventions will be considered rhythm control groups, while the two groups receiving rate control interventions will be considered rate control groups.

If quantitative synthesis is not appropriate, we will report the results in a narrative way.

Trial Sequential Analysis Traditional meta-analysis runs the risk of random errors due to sparse data and repetitive testing of accumulating data when updating reviews. We wish to control the risks of type I errors and type II errors. We will therefore perform Trial Sequential Analysis on the outcomes, in order to calculate the required information size (that is the number of participants needed in a meta-analysis to detect or reject a certain intervention effect) and the cumulative Z-curve's breach of relevant trial sequential monitoring boundaries [77, 84, 103–110]. A more detailed description of Trial Sequential Analysis can be found in the Trial Sequential Analysis manual [108] and at http://www.ctu.dk/tsa/.

For dichotomous outcomes, we will estimate the required information size based on the observed proportion of patients with an outcome in the control group (the cumulative proportion of patients with an event in the control groups relative to all patients in the control groups), a relative risk reduction of 15%, an alpha of

2.5% for our primary outcomes and an alpha of 3.3% for our secondary outcomes, a beta of 10%, and diversity as suggested by the trials in the meta-analysis. For continuous outcomes, we will in the Trial Sequential Analysis use the observed SD, a mean difference of the observed SD/2, an alpha of 2.5% for our primary outcomes and an alpha of 3.3% for our secondary outcomes, and a beta of 10%.

Subgroup analysis and investigation of heterogeneity

Subgroup analysis We will perform the following subgroup analysis when analysing the primary outcomes (all-cause mortality, serious adverse event, and quality of life).

1. High risk of bias trials compared to low risk of bias trials
2. Comparison of individual rhythm control interventions with any rate control intervention
3. Comparison of individual rate control interventions with any rhythm control intervention
4. Participants with atrial fibrillation compared to participants with atrial flutter
5. Age of participants: 0 to 59 years, 60 to 79 years, and above 80 years
6. Duration of atrial fibrillation: recent-onset atrial fibrillation (as defined by the trialists), paroxysmal atrial fibrillation (less than 7 days of onset), persistent atrial fibrillation (more than 7 days and less than 1 year of onset), and long-standing persistent atrial fibrillation (more than 1 year of onset)
7. Duration of anticoagulation therapy: anticoagulation therapy until sinus rhythm for at least 4 weeks, anticoagulation therapy until sinus rhythm for at least 12 weeks, or anticoagulation therapy until end of follow-up
8. Men compared to women

We will use the formal test for subgroup interactions in Review Manager [83].

Sensitivity analysis To assess the potential impact of the missing data for dichotomous outcomes, we will perform the two following sensitivity analyses on both the primary and secondary outcomes.

- 'Best-worst-case' scenario: We will assume that all participants lost to follow-up in the rhythm control group have survived, had no serious adverse event, and had no stroke and that all those participants lost to follow-up in the rate control group have not survived, had a serious adverse event, and had a stroke.
- 'Worst-best-case' scenario: We will assume that all participants lost to follow-up in the rhythm control group have not survived, had a serious adverse event, and had a stroke and that all those participants lost to follow-up in the rate control group have survived, had no serious adverse event, and had no stroke.

We will present results of both scenarios in our review.

When analysing quality of life or ejection fraction, a 'beneficial outcome' will be the group mean plus two standard deviations (SDs) (we will secondly use one SD in another sensitivity analysis) of the group mean and a 'harmful outcome' will be the group mean minus two SDs (we will secondly use one SD in another sensitivity analysis) of the group mean [76].

To assess the potential impact of missing SDs for continuous outcomes, we will perform the following sensitivity analysis.

- Where SDs are missing and it is not possible to calculate them, we will impute SDs from trials with similar populations and low risk of bias. If we find no such trials, we will impute SDs from trials with a similar population. As the final option, we will impute SDs from all trials.

We will present results of this scenario in our review.

Other post hoc sensitivity analyses might be warranted if unexpected clinical or statistical heterogeneity is identified during the analysis of the review results [76].

'Summary of Findings' table We will create a Summary of Findings table using each of the prespecified outcomes (all-cause mortality, serious adverse event, quality of life, stroke, and ejection fraction). We will use the five GRADE considerations (bias risk of the trials, consistency of effect, imprecision, indirectness, and publication bias) to assess the quality of a body of evidence as it relates to the studies which contribute data to the meta-analyses for the prespecified outcomes [76, 111–113]. We will use methods and recommendations described in Chapter 8 (Section 8.5) and Chapter 12 of the *Cochrane Handbook for Systematic Reviews of Interventions* [79] using GRADEpro software. We will justify all decisions to downgrade the quality of studies using footnotes, and we will make comments to aid the reader's understanding of the review where necessary. Firstly, we will present our results in the Summary of Findings table based on the results from the trials with low risk of bias, and secondly, we will present the results based on all trials.

Discussion

This protocol aims at comparing the effects of rhythm control strategies with the effects of rate control strategies in patients with atrial fibrillation and atrial flutter to determine the best overall treatment strategy. The outcomes will be all-cause mortality, serious adverse events, quality of life, stroke, and ejection fraction.

This protocol has a number of strengths. The predefined methodology is based on the *Cochrane Handbook for Systematic Reviews of Interventions* [79], the eight-step assessment suggested by Jakobsen et al. [76], Trial Sequential Analysis [84], and GRADE assessment [111–113]. Hence, this protocol takes into account both risks of random errors and risks of systematic errors. Another strength of this protocol is that we pragmatically compare two overall treatment strategies with each other, i.e. the results of this review will potentially reflect the effects of the two strategies in clinical everyday practice.

Our protocol also has a number of limitations. The primary limitation is that both of the strategies we compare consist of multiple treatment elements and it is likely that different interventions have different effects. Hence, if we show a difference between the compared strategies, it will be difficult to conclude what exactly caused the difference in effect. To minimise this limitation, a number of subgroups are planned, but results of subgroup analyses should always be interpreted with great caution. Another limitation is the large number of comparisons which increase the risk of type 1 error. We have adjusted our thresholds for significance according to the number of primary outcomes, but, as mentioned, we have also included multiple subgroup analyses. This large risk of type 1 error will be taken into account when interpreting the review results.

Additional files

Additional file 1: PRISMA-P checklist. (DOCX 30 kb)

Additional file 2: The preliminary search strategy for MEDLINE (Ovid). (PDF 221 kb)

Abbreviations

AFFIRM: The Atrial Fibrillation Follow-up Investigation of Rhythm Management; CAST: Cardiac Arrhythmia Suppression Trial; CENTRAL: The Cochrane Central Register of Controlled Trials; CFDA: China Food and Drug Administration; CI: Confidence interval; ECG: Electrocardiogram; EMA: European Medicines Agency; EMBASE: Excerpta Medica database; GRADE: The Grades of Recommendation, Assessment, Development, and Evaluation; LILACS: Latin American and Caribbean Health Sciences Literature; MD: Mean difference; MEDLINE: Medical Literature Analysis and Retrieval System Online; PRISMA: Preferred Reporting Items for Systematic Reviews and Meta-Analyses; PRISMA-P: Preferred Reporting Items for Systematic Reviews and Meta-Analysis Protocols; PROSPERO: International Prospective Register of Systematic Reviews; RR: Risk ratio; SMD: Standardised mean difference; TRIP: Turning Research Into Practice; WHO: World Health Organization

Acknowledgements

The expert help from Sarah Louise Klingenberg (Information Specialist, The Cochrane Hepato-Biliary Group, Copenhagen Trial Unit, Copenhagen, Denmark) in making the search strategy is hugely appreciated.
We thank the *Systematic Reviews* for providing us with the PRISMA-P checklist to optimise the protocol. The PRISMA-P checklist is given in Additional file 1.

Funding

No funding has been received for this protocol.

Availability of data and materials

Not applicable.

Authors' contributions

NJS drafted the protocol. JCJ, SS, JF, EEN, and CG amended the protocol. All authors read and approved the final manuscript.

Competing interests

The authors declare that they have no competing interests.

Consent for publication

Not applicable.

Ethics approval and consent to participate

Not applicable.

Sources of support

The making of this protocol was supported by Copenhagen Trial Unit, Centre for Clinical Intervention Research, Rigshospitalet, Copenhagen, Denmark.

Author details

[1]Copenhagen Trial Unit, Centre for Clinical Intervention Research, Department 7812, Rigshospitalet, Copenhagen University Hospital, Copenhagen, Denmark. [2]The Cochrane Hepato-Biliary Group, Copenhagen Trial Unit, Centre for Clinical Intervention Research, Department 7812, Rigshospitalet, Copenhagen University Hospital, Copenhagen, Denmark. [3]Department of Cardiology, Holbæk Hospital, Holbæk, Denmark.

Received: 17 November 2016 Accepted: 28 February 2017
Published online: 06 March 2017

References

1. Pistoia F, Sacco S, Tiseo C, Degan D, Ornello R, Carolei A. The epidemiology of atrial fibrillation and stroke. Cardiol Clin. 2016;34(2):255–68.
2. Camm AJ, Lip GY, De Caterina R, Savelieva I, Atar D, Hohnloser SH, Hindricks G, Kirchhof P. 2012 focused update of the ESC Guidelines for the management of atrial fibrillation: an update of the 2010 ESC Guidelines for the management of atrial fibrillation. Europace. 2012;14(10):1385–413.
3. Granada J, Uribe W, Chyou PH, Maassen K, Vierkant R, Smith PN, Hayes J, Eaker E, Vidaillet H. Incidence and predictors of atrial flutter in the general population. J Am Coll Cardiol. 2000;36(7):2242–6.
4. Chugh SS, Havmoeller R, Narayanan K, Singh D, Rienstra M, Benjamin EJ, Gillum RF, Kim YH, McAnulty Jr JH, Zheng ZJ, et al. Worldwide epidemiology of atrial fibrillation: a global burden of disease 2010 study. Circulation. 2014;129(8):837–47.
5. Fitzmaurice DA, Hobbs FD, Jowett S, Mant J, Murray ET, Holder R, Raftery JP, Bryan S, Davies M, Lip GY, et al. Screening versus routine practice in detection of atrial fibrillation in patients aged 65 or over: cluster randomised controlled trial. BMJ. 2007;335(7616):383.
6. Stewart S, Hart CL, Hole DJ, McMurray JJ. A population-based study of the long-term risks associated with atrial fibrillation: 20-year follow-up of the Renfrew/Paisley study. Am J Med. 2002;113(5):359–64.
7. Benjamin EJ, Wolf PA, D'Agostino RB, Silbershatz H, Kannel WB, Levy D. Impact of atrial fibrillation on the risk of death: the Framingham Heart Study. Circulation. 1998;98(10):946–52.
8. Rahman F, Wang N, Yin X, Ellinor PT, Lubitz SA, LeLorier PA, McManus DD, Sullivan LM, Seshadri S, Vasan RS, et al. Atrial flutter: clinical risk factors and adverse outcomes in the Framingham Heart Study. Heart Rhythm. 2016; 13(1):233–40.
9. Healey JS, Oldgren J, Ezekowitz M, Zhu J, Pais P, Wang J, Commerford P, Jansky P, Avezum A, Sigamani A, et al. Occurrence of death and stroke in

patients in 47 countries 1 year after presenting with atrial fibrillation: a cohort study. Lancet. 2016;388(10050):1161–9.
10. Wolf PA, Abbott RD, Kannel WB. Atrial fibrillation as an independent risk factor for stroke: the Framingham Study. Stroke. 1991;22(8):983–8.
11. Odutayo A, Wong CX, Hsiao AJ, Hopewell S, Altman DG, Emdin CA. Atrial fibrillation and risks of cardiovascular disease, renal disease, and death: systematic review and meta-analysis. BMJ. 2016;354:i4482.
12. Go AS, Hylek EM, Phillips KA, Chang Y, Henault LE, Selby JV, Singer DE. Prevalence of diagnosed atrial fibrillation in adults: national implications for rhythm management and stroke prevention: the Anticoagulation and Risk Factors in Atrial Fibrillation (ATRIA) Study. JAMA. 2001;285(18):2370–5.
13. Stewart S, Murphy NF, Walker A, McGuire A, McMurray JJ. Cost of an emerging epidemic: an economic analysis of atrial fibrillation in the UK. Heart. 2004;90(3):286–92.
14. Mozaffarian D, Benjamin EJ, Go AS, Arnett DK, Blaha MJ, Cushman M, Das SR, de Ferranti S, Despres JP, Fullerton HJ, et al. Heart disease and stroke statistics—2016 update: a report from the American Heart Association. Circulation. 2016;133(4):e38–360.
15. Calkins H, Kuck KH, Cappato R, Brugada J, Camm AJ, Chen SA, Crijns HJ, Damiano Jr RJ, Davies DW, DiMarco J, et al. 2012 HRS/EHRA/ECAS expert consensus statement on catheter and surgical ablation of atrial fibrillation: recommendations for patient selection, procedural techniques, patient management and follow-up, definitions, endpoints, and research trial design. Europace. 2012;14(4):528–606.
16. January CT, Wann LS, Alpert JS, Calkins H, Cigarroa JE, Cleveland Jr JC, Conti JB, Ellinor PT, Ezekowitz MD, Field ME, et al. 2014 AHA/ACC/HRS guideline for the management of patients with atrial fibrillation: a report of the American College of Cardiology/American Heart Association Task Force on Practice Guidelines and the Heart Rhythm Society. J Am Coll Cardiol. 2014; 64(21):e1–76.
17. Camm AJ, Kirchhof P, Lip GY, Schotten U, Savelieva I, Ernst S, Van Gelder IC, Al-Attar N, Hindricks G, Prendergast B, et al. Guidelines for the management of atrial fibrillation: the task force for the management of atrial fibrillation of the European Society of Cardiology (ESC). Europace. 2010;12(10):1360–420.
18. Wyse DG. Therapeutic considerations in applying rate control therapy for atrial fibrillation. J Cardiovasc Pharmacol. 2008;52(1):11–7.
19. Daoud EG, Weiss R, Bahu M, Knight BP, Bogun F, Goyal R, Harvey M, Strickberger SA, Man KC, Morady F. Effect of an irregular ventricular rhythm on cardiac output. Am J Cardiol. 1996;78(12):1433–6.
20. Gopinathannair R, Etheridge SP, Marchlinski FE, Spinale FG, Lakkireddy D, Olshansky B. Arrhythmia-induced cardiomyopathies: mechanisms, recognition, and management. J Am Coll Cardiol. 2015;66(15):1714–28.
21. Lip GY, Fauchier L, Freedman SB, Van Gelder I, Natale A, Gianni C, Nattel S, Potpara T, Rienstra M, Tse HF, et al. Atrial fibrillation. Nat Rev Dis Primers. 2016;2:16016.
22. Page RL, Joglar JA, Caldwell MA, Calkins H, Conti JB, Deal BJ, Estes 3rd NA, Field ME, Goldberger ZD, Hammill SC, et al. 2015 ACC/AHA/HRS guideline for the management of adult patients with supraventricular tachycardia: a report of the American College of Cardiology/American Heart Association Task Force on Clinical Practice Guidelines and the Heart Rhythm Society. J Am Coll Cardiol. 2016;67(13):e27–e115.
23. Fauchier L, Philippart R, Clementy N, Bourguignon T, Angoulvant D, Ivanes F, Babuty D, Bernard A. How to define valvular atrial fibrillation? Arch Cardiovasc Dis. 2015;108(10):530–9.
24. De Caterina R, Camm AJ. What is 'valvular' atrial fibrillation? A reappraisal. Eur Heart J. 2014;35(47):3328–35.
25. Markides V, Schilling RJ. Atrial fibrillation: classification, pathophysiology, mechanisms and drug treatment. Heart. 2003;89(8):939–43.
26. Saoudi N, Cosio F, Waldo A, Chen SA, Iesaka Y, Lesh M, Saksena S, Salerno J, Schoels W. Classification of atrial flutter and regular atrial tachycardia according to electrophysiologic mechanism and anatomic bases: a statement from a joint expert group from the Working Group of Arrhythmias of the European Society of Cardiology and the North American Society of Pacing and Electrophysiology. J Cardiovasc Electrophysiol. 2001; 12(7):852–66.
27. Haissaguerre M, Jais P, Shah DC, Takahashi A, Hocini M, Quiniou G, Garrigue S, Le Mouroux A, Le Metayer P, Clementy J. Spontaneous initiation of atrial fibrillation by ectopic beats originating in the pulmonary veins. NEJM. 1998; 339(10):659–66.
28. Aviles RJ, Martin DO, Apperson-Hansen C, Houghtaling PL, Rautaharju P, Kronmal RA, Tracy RP, Van Wagoner DR, Psaty BM, Lauer MS, et al. Inflammation as a risk factor for atrial fibrillation. Circulation. 2003;108(24): 3006–10.
29. Burstein B, Nattel S. Atrial fibrosis: mechanisms and clinical relevance in atrial fibrillation. J Am Coll Cardiol. 2008;51(8):802–9.
30. Morillo CA, Klein GJ, Jones DL, Guiraudon CM. Chronic rapid atrial pacing. Structural, functional, and electrophysiological characteristics of a new model of sustained atrial fibrillation. Circulation. 1995;91(5):1588–95.
31. Wijffels MC, Kirchhof CJ, Dorland R, Allessie MA. Atrial fibrillation begets atrial fibrillation. A study in awake chronically instrumented goats. Circulation. 1995;92(7):1954–68.
32. Lip GY, Nieuwlaat R, Pisters R, Lane DA, Crijns HJ. Refining clinical risk stratification for predicting stroke and thromboembolism in atrial fibrillation using a novel risk factor-based approach: the euro heart survey on atrial fibrillation. Chest. 2010;137(2):263–72.
33. Pisters R, Lane DA, Nieuwlaat R, de Vos CB, Crijns HJ, Lip GY. A novel user-friendly score (HAS-BLED) to assess 1-year risk of major bleeding in patients with atrial fibrillation: the Euro Heart Survey. Chest. 2010;138(5): 1093–100.
34. Connolly SJ, Eikelboom J, Joyner C, Diener HC, Hart R, Golitsyn S, Flaker G, Avezum A, Hohnloser SH, Diaz R, et al. Apixaban in patients with atrial fibrillation. NEJM. 2011;364(9):806–17.
35. Shi XX, Ren GH, Wang J, Zhang N, Yu MZ, Wang YQ, Shao YK. Effectiveness and safety of warfarin and anti-platelet drugs for the primary prevention of stroke in patients with non-valvular atrial fibrillation: a meta-analysis. Int J Clin Exp Med. 2015;8(6):8384–97.
36. Ruff CT, Giugliano RP, Braunwald E, Hoffman EB, Deenadayalu N, Ezekowitz MD, Camm AJ, Weitz JI, Lewis BS, Parkhomenko A, et al. Comparison of the efficacy and safety of new oral anticoagulants with warfarin in patients with atrial fibrillation: a meta-analysis of randomised trials. Lancet. 2014;383(9921):955–62.
37. Vaughan Williams EM. A classification of antiarrhythmic actions reassessed after a decade of new drugs. J Clin Pharmacol. 1984;24(4):129–47.
38. The Sicilian gambit. A new approach to the classification of antiarrhythmic drugs based on their actions on arrhythmogenic mechanisms. Task Force of the Working Group on Arrhythmias of the European Society of Cardiology. Circulation. 1991;84(4):1831–51.
39. Fuster V, Ryden LE, Cannom DS, Crijns HJ, Curtis AB, Ellenbogen KA, Halperin JL, Kay GN, Le Huezey JY, Lowe JE, et al. 2011 ACCF/AHA/HRS focused updates incorporated into the ACC/AHA/ESC 2006 guidelines for the management of patients with atrial fibrillation: a report of the American College of Cardiology Foundation/American Heart Association Task Force on practice guidelines. Circulation. 2011;123(10):e269–367.
40. Bash LD, Buono JL, Davies GM, Martin A, Fahrbach K, Phatak H, Avetisyan R, Mwamburi M. Systematic review and meta-analysis of the efficacy of cardioversion by vernakalant and comparators in patients with atrial fibrillation. Cardiovasc Drugs Ther. 2012;26(2):167–79.
41. Lafuente-Lafuente C, Valembois L, Bergmann JF, Belmin J: Antiarrhythmics for maintaining sinus rhythm after cardioversion of atrial fibrillation. Cochrane Database Syst Rev. 2015;(3):CD005049. doi:10.1002/14651858. CD005049.pub4.
42. Kirchhof P, Benussi S, Kotecha D, Ahlsson A, Atar D, Casadei B, Castella M, Diener HC, Heidbuchel H, Hendriks J et al. 2016 ESC Guidelines for the management of atrial fibrillation developed in collaboration with EACTS. Eur Heart J. 2016;37(38):2893–2962.
43. The Cardiac Arrhythmia Suppression Trial (CAST) Investigators. Preliminary report: effect of encainide and flecainide on mortality in a randomized trial of arrhythmia suppression after myocardial infarction. NEJM. 1989;321(6):406–12.
44. Singh BN, Singh SN, Reda DJ, Tang XC, Lopez B, Harris CL, Fletcher RD, Sharma SC, Atwood JE, Jacobson AK, et al. Amiodarone versus sotalol for atrial fibrillation. NEJM. 2005;352(18):1861–72.
45. Roy D, Talajic M, Dorian P, Connolly S, Eisenberg MJ, Green M, Kus T, Lambert J, Dubuc M, Gagne P, et al. Amiodarone to prevent recurrence of atrial fibrillation. NEJM. 2000;342(13):913–20. Canadian Trial of Atrial Fibrillation Investigators.
46. Freemantle N, Lafuente-Lafuente C, Mitchell S, Eckert L, Reynolds M. Mixed treatment comparison of dronedarone, amiodarone, sotalol, flecainide, and propafenone, for the management of atrial fibrillation. Europace. 2011;13(3):329–45.
47. Mittal S, Ayati S, Stein KM, Schwartzman D, Cavlovich D, Tchou PJ, Markowitz SM, Slotwiner DJ, Scheiner MA, Lerman BB. Transthoracic

cardioversion of atrial fibrillation: comparison of rectilinear biphasic versus damped sine wave monophasic shocks. Circulation. 2000;101(11):1282–87.
48. Page RL, Kerber RE, Russell JK, Trouton T, Waktare J, Gallik D, Olgin JE, Ricard P, Dalzell GW, Reddy R, et al. Biphasic versus monophasic shock waveform for conversion of atrial fibrillation: the results of an international randomized, double-blind multicenter trial. J Am Coll Cardiol. 2002;39(12): 1956–63.
49. Inacio JF, da Rosa MS, Shah J, Rosario J, Vissoci JR, Manica AL, Rodrigues CG. Monophasic and biphasic shock for transthoracic conversion of atrial fibrillation: systematic review and network meta-analysis. Resuscitation. 2016;100:66–75.
50. Reisinger J, Gstrein C, Winter T, Zeindlhofer E, Hollinger K, Mori M, Schiller A, Winter A, Geiger H, Siostrzonek P. Optimization of initial energy for cardioversion of atrial tachyarrhythmias with biphasic shocks. Am J Emerg Med. 2010;28(2):159–65.
51. Pinski SL, Sgarbossa EB, Ching E, Trohman RG. A comparison of 50-J versus 100-J shocks for direct-current cardioversion of atrial flutter. Am Heart J. 1999;137(3):439–42.
52. Frick M, Frykman V, Jensen-Urstad M, Ostergren J, Rosenqvist M. Factors predicting success rate and recurrence of atrial fibrillation after first electrical cardioversion in patients with persistent atrial fibrillation. Clin Cardiol. 2001; 24(3):238–44.
53. Van Gelder IC, Crijns HJ, Van Gilst WH, Verwer R, Lie KI. Prediction of uneventful cardioversion and maintenance of sinus rhythm from direct-current electrical cardioversion of chronic atrial fibrillation and flutter. Am J Cardiol. 1991;68(1):41–6.
54. Psotka MA, Lee BK. Atrial fibrillation: antiarrhythmic therapy. Curr Probl Cardiol. 2014;39(10):351–91.
55. Wu RC, Berger R, Calkins H. Catheter ablation of atrial flutter and macroreentrant atrial tachycardia. Curr Opin Cardiol. 2002;17(1):58–64.
56. Cappato R, Calkins H, Chen SA, Davies W, Iesaka Y, Kalman J, Kim YH, Klein G, Natale A, Packer D, et al. Updated worldwide survey on the methods, efficacy, and safety of catheter ablation for human atrial fibrillation. Circ Arrhythm Electrophysiol. 2010;3(1):32–8.
57. Lawrance CP, Henn MC, Damiano Jr RJ. Surgery for atrial fibrillation. Heart Fail Clin. 2016;12(2):235–43.
58. Cox JL. Surgery for cardiac arrhythmias. Curr Probl Cardiol. 1983;8(4):1–60.
59. Gillinov AM, Gelijns AC, Parides MK, DeRose Jr JJ, Moskowitz AJ, Voisine P, Ailawadi G, Bouchard D, Smith PK, Mack MJ, et al. Surgical ablation of atrial fibrillation during mitral-valve surgery. NEJM. 2015;372(15):1399–409.
60. Shinbane JS, Wood MA, Jensen DN, Ellenbogen KA, Fitzpatrick AP, Scheinman MM. Tachycardia-induced cardiomyopathy: a review of animal models and clinical studies. J Am Coll Cardiol. 1997;29(4):709–15.
61. Chao TF, Liu CJ, Tuan TC, Chen SJ, Wang KL, Lin YJ, Chang SL, Lo LW, Hu YF, Chen TJ, et al. Rate-control treatment and mortality in atrial fibrillation. Circulation. 2015;132(17):1604–12.
62. Segal JB, McNamara RL, Miller MR, Kim N, Goodman SN, Powe NR, Robinson K, Yu D, Bass EB. The evidence regarding the drugs used for ventricular rate control. J Fam Pract. 2000;49(1):47–59.
63. Olshansky B, Rosenfeld LE, Warner AL, Solomon AJ, O'Neill G, Sharma A, Platia E, Feld GK, Akiyama T, Brodsky MA, et al. The Atrial Fibrillation Follow-up Investigation of Rhythm Management (AFFIRM) study: approaches to control rate in atrial fibrillation. J Am Coll Cardiol. 2004;43(7):1201–8.
64. Whitbeck MG, Charnigo RJ, Khairy P, Ziada K, Bailey AL, Zegarra MM, Shah J, Morales G, Macaulay T, Sorrell VL, et al. Increased mortality among patients taking digoxin—analysis from the AFFIRM study. Eur Heart J. 2013;34(20): 1481–8.
65. Shah M, Avgil Tsadok M, Jackevicius CA, Essebag V, Behlouli H, Pilote L. Relation of digoxin use in atrial fibrillation and the risk of all-cause mortality in patients ≥65 years of age with versus without heart failure. Am J Cardiol. 2014;114(3):401–6.
66. Okin PM, Hille DA, Wachtell K, Kjeldsen SE, Boman K, Dahlof B, Devereux RB. Digoxin use and risk of mortality in hypertensive patients with atrial fibrillation. J Hypertens. 2015;33(7):1480–6.
67. Mulder BA, Van Veldhuisen DJ, Crijns HJ, Tijssen JG, Hillege HL, Alings M, Rienstra M, Van den Berg MP, Van Gelder IC. Digoxin in patients with permanent atrial fibrillation: data from the RACE II study. Heart Rhythm. 2014;11(9):1543–50.
68. Gheorghiade M, Fonarow GC, van Veldhuisen DJ, Cleland JG, Butler J, Epstein AE, Patel K, Aban IB, Aronow WS, Anker SD, et al. Lack of evidence of increased mortality among patients with atrial fibrillation taking digoxin: findings from post hoc propensity-matched analysis of the AFFIRM trial. Eur Heart J. 2013;34(20):1489–97.
69. Ponikowski P, Voors AA, Anker SD, Bueno H, Cleland JG, Coats AJ, Falk V, Gonzalez-Juanatey JR, Harjola VP, Jankowska EA, et al. 2016 ESC guidelines for the diagnosis and treatment of acute and chronic heart failure: the task force for the diagnosis and treatment of acute and chronic heart failure of the European Society of Cardiology (ESC). Developed with the special contribution of the Heart Failure Association (HFA) of the ESC. Eur Heart J. 2016;37(27):2129–200.
70. Wood MA, Brown-Mahoney C, Kay GN, Ellenbogen KA. Clinical outcomes after ablation and pacing therapy for atrial fibrillation: a meta-analysis. Circulation. 2000;101(10):1138–44.
71. Patel D, Daoud EG. Atrioventricular junction ablation for atrial fibrillation. Heart Fail Clin. 2016;12(2):245–55.
72. Al-Khatib SM, Allen LaPointe NM, Chatterjee R, Crowley MJ, Dupre ME, Kong DF, Lopes RD, Povsic TJ, Raju SS, Shah B, et al. Rate- and rhythm-control therapies in patients with atrial fibrillation: a systematic review. Ann Intern Med. 2014;160(11):760–73.
73. Cordina J, Mead G. Pharmacological cardioversion for atrial fibrillation and flutter. Cochrane Database Syst Rev. 2005;(2):CD003713. doi:10.1002/14651858.CD003713.pub2.
74. Chatterjee S, Sardar P, Lichstein E, Mukherjee D, Aikat S. Pharmacologic rate versus rhythm-control strategies in atrial fibrillation: an updated comprehensive review and meta-analysis. PACE. 2013;36(1):122–33.
75. Caldeira D, David C, Sampaio C. Rate versus rhythm control in atrial fibrillation and clinical outcomes: updated systematic review and meta-analysis of randomized controlled trials. Arch Cardiovasc Dis. 2012;105(4):226–38.
76. Jakobsen JC, Wetterslev J, Winkel P, Lange T, Gluud C. Thresholds for statistical and clinical significance in systematic reviews with meta-analytic methods. BMC Med Res Methodol. 2014;14:120.
77. Wetterslev J, Thorlund K, Brok J, Gluud C. Trial sequential analysis may establish when firm evidence is reached in cumulative meta-analysis. J Clin Epidemiol. 2008;61(1):64–75.
78. Keus F, Wetterslev J, Gluud C, van Laarhoven CJ. Evidence at a glance: error matrix approach for overviewing available evidence. BMC Med Res Methodol. 2010;10:90.
79. Higgins J, Green S. Cochrane Handbook for Systematic Reviews of Interventions version 5.1.0 *The Cochrane Collaboration*. 2011 www.handbook.cochrane.org.
80. Moher D, Shamseer L, Clarke M, Ghersi D, Liberati A, Petticrew M, Shekelle P, Stewart LA. Preferred Reporting Items for Systematic Review and Meta-Analysis Protocols (PRISMA-P) 2015 statement. Syst Rev. 2015;4:1.
81. Shamseer L, Moher D, Clarke M, Ghersi D, Liberati A, Petticrew M, Shekelle P, Stewart LA. Preferred Reporting Items for Systematic Review and Meta-Analysis Protocols (PRISMA-P) 2015: elaboration and explanation. BMJ. 2015;349:g7647.
82. International conference on harmonisation of technical requirements for registration of pharmaceuticals for human use (ICH) adopts consolidated guideline on good clinical practice in the conduct of clinical trials on medicinal products for human use. Int Dig Health Legis. 1997;48(2):231-234.
83. Review Manager (RevMan) [Computer program]. Version 5.3. Copenhagen: the Nordic Cochrane Centre, The Cochrane Collaboration. 2014.
84. Copenhagen Trial Unit. TSA—trial sequential analysis. http://www.ctu.dk/tsa/.
85. StataCorp: Stata: Release 14. Statistical software. College Station, TX: StataCorp LP. 2014. http://www.stata.com.
86. Moher D, Liberati A, Tetzlaff J, Altman DG. Preferred Reporting Items for Systematic Reviews and Meta-Analyses: the PRISMA statement. Ann Intern Med. 2009;151(4):264–9. w264.
87. Gluud LL. Bias in clinical intervention research. Am J Epidemiol. 2006;163(6):493–501.
88. Kjaergard LL, Villumsen J, Gluud C. Reported methodologic quality and discrepancies between large and small randomized trials in meta-analyses. Ann Intern Med. 2001;135(11):982–9.
89. Lundh A, Sismondo S, Lexchin J, Busuioc OA, Bero L. Industry sponsorship and research outcome. Cochrane Database Syst Rev. 2012;12:Mr000033.
90. Moher D, Pham B, Jones A, Cook DJ, Jadad AR, Moher M, Tugwell P, Klassen TP. Does quality of reports of randomised trials affect estimates of intervention efficacy reported in meta-analyses? Lancet. 1998;352(9128):609–13.

91. Schulz KF, Chalmers I, Hayes RJ, Altman DG. Empirical evidence of bias. Dimensions of methodological quality associated with estimates of treatment effects in controlled trials. JAMA. 1995;273(5):408–12.
92. Wood L, Egger M, Gluud LL, Schulz KF, Juni P, Altman DG, Gluud C, Martin RM, Wood AJ, Sterne JA. Empirical evidence of bias in treatment effect estimates in controlled trials with different interventions and outcomes: meta-epidemiological study. BMJ. 2008;336(7644):601–5.
93. Savovic J, Jones H, Altman D, Harris R, Juni P, Pildal J, Als-Nielsen B, Balk E, Gluud C, Gluud L, et al. Influence of reported study design characteristics on intervention effect estimates from randomised controlled trials: combined analysis of meta-epidemiological studies. Health Technol Assess. 2012;16(35):1–82.
94. Higgins JP, Thompson SG. Quantifying heterogeneity in a meta-analysis. Stat Med. 2002;21(11):1539–58.
95. Higgins JP, Thompson SG, Deeks JJ, Altman DG. Measuring inconsistency in meta-analyses. BMJ. 2003;327(7414):557–60.
96. Harbord RM, Egger M, Sterne JA. A modified test for small-study effects in meta-analyses of controlled trials with binary endpoints. Stat Med. 2006; 25(20):3443–57.
97. Egger M, Davey Smith G, Schneider M, Minder C. Bias in meta-analysis detected by a simple, graphical test. BMJ. 1997;315(7109):629–34.
98. Begg CB, Mazumdar M. Operating characteristics of a rank correlation test for publication bias. Biometrics. 1994;50(4):1088–101.
99. Elbourne DR, Altman DG, Higgins JP, Curtin F, Worthington HV, Vail A. Meta-analyses involving cross-over trials: methodological issues. Int J Epidemiol. 2002;31(1):140–9.
100. DerSimonian R, Laird N. Meta-analysis in clinical trials. Control Clin Trials. 1986;7(3):177–88.
101. Demets DL. Methods for combining randomized clinical trials: strengths and limitations. Stat Med. 1987;6(3):341–50.
102. Jakobsen JC, Wetterslev J, Lange T, Gluud C. Viewpoint: taking into account risks of random errors when analysing multiple outcomes in systematic reviews. Cochrane Database Syst Rev. 2016;3:Ed000111.
103. Brok J, Thorlund K, Gluud C, Wetterslev J. Trial sequential analysis reveals insufficient information size and potentially false positive results in many meta-analyses. J Clin Epidemiol. 2008;61(8):763–9.
104. Brok J, Thorlund K, Wetterslev J, Gluud C. Apparently conclusive meta-analyses may be inconclusive—trial sequential analysis adjustment of random error risk due to repetitive testing of accumulating data in apparently conclusive neonatal meta-analyses. Int J Epidemiol. 2009;38(1):287–98.
105. Thorlund K, Devereaux PJ, Wetterslev J, Guyatt G, Ioannidis JP, Thabane L, Gluud LL, Als-Nielsen B, Gluud C. Can trial sequential monitoring boundaries reduce spurious inferences from meta-analyses? Int J Epidemiol. 2009;38(1):276–86.
106. Wetterslev J, Thorlund K, Brok J, Gluud C. Estimating required information size by quantifying diversity in random-effects model meta-analyses. BMC Med Res Methodol. 2009;9:86.
107. Thorlund K, Anema A, Mills E. Interpreting meta-analysis according to the adequacy of sample size. An example using isoniazid chemoprophylaxis for tuberculosis in purified protein derivative negative HIV-infected individuals. Clin Epidemiol. 2010;2:57–66.
108. Thorlund K EJ, Wetterslev J, Brok J, Imberger G, Gluud C. User manual for trial sequential analysis (TSA). 2011. http://www.ctu.dk/tsa/files/tsa_manual.pdf.
109. Imberger G, Gluud C, Boylan J, Wetterslev J. Systematic reviews of anesthesiologic interventions reported as statistically significant: problems with power, precision, and type 1 error protection. Anesth Analg. 2015; 121(6):1611–22.
110. Imberger G, Thorlund K, Gluud C, Wetterslev J. False-positive findings in Cochrane meta-analyses with and without application of trial sequential analysis: an empirical review. BMJ Open. 2016;6(8):e011890.
111. Guyatt GH, Oxman AD, Vist GE, Kunz R, Falck-Ytter Y, Alonso-Coello P, Schunemann HJ. GRADE: an emerging consensus on rating quality of evidence and strength of recommendations. BMJ. 2008;336(7650):924–6.
112. Schunemann HJ, Best D, Vist G, Oxman AD. Letters, numbers, symbols and words: how to communicate grades of evidence and recommendations. CMAJ. 2003;169(7):677–80.
113. Guyatt GH, Oxman AD, Schunemann HJ, Tugwell P, Knottnerus A. GRADE guidelines: a new series of articles in the Journal of Clinical Epidemiology. J Clin Epidemiol. 2011;64(4):380–2.

Submit your next manuscript to BioMed Central and we will help you at every step:

- We accept pre-submission inquiries
- Our selector tool helps you to find the most relevant journal
- We provide round the clock customer support
- Convenient online submission
- Thorough peer review
- Inclusion in PubMed and all major indexing services
- Maximum visibility for your research

Submit your manuscript at
www.biomedcentral.com/submit

BioMed Central
