# Supplementary material for: The effects of rhythm control strategies versus rate control strategies for atrial fibrillation and atrial flutter: A systematic review with meta-analysis and Trial Sequential Analysis
Source: PLoS One. 2017 Oct 26;12(10):e0186856. doi: 10.1371/journal.pone.0186856 (PMC5658096; doi:10.1371/journal.pone.0186856)
Supplement: S3 Text — (PDF) [file pone.0186856.s003.pdf]

Search strategy for MEDLINE (OvidSP; 1946 to October 2016)

1. exp Atrial Fibrillation/

2. exp Atrial Flutter/

3. (atri* and (fibrillation* or flutter*)).mp. [mp=title, abstract, original title, name of substance word, subject heading word, keyword heading word, protocol supplementary concept word, rare disease supplementary concept word, unique identifier]

4. 1 or 2 or 3

5. exp Heart Rate/

6. exp Anti-Arrhythmia Agents/

7. (((rate or rhythm) and control) or antiarrythmic therap* or ((atrioventricular node or catheter or surgical) and ablation*) or cardioversion*).mp. [mp=title, abstract, original title, name of substance word, subject heading word, keyword heading word, protocol supplementary concept word, rare disease supplementary concept word, unique identifier]

8. ((beta or calcium channel) and blocker*).mp. [mp=title, abstract, original title, name of substance word, subject heading word, keyword heading word, protocol supplementary concept word, rare disease supplementary concept word, unique identifier]

9. (Propranolol or carvediol or esmolol or timolol or metoprolol or atenolol or bisoprolol or nebivolol or verapamil or diltiazem or digoxin or flecainide or propafenone or encainide or moricizine or dofetilide or amiodarone or sotalol or disopyramide or quinidine or ajmaline or procainamide or dronedarone or ibutilide or lidocaine or phenytoin or mexiletine or tocainide).mp. [mp=title, abstract, original title, name of substance word, subject heading word, keyword heading word, protocol supplementary concept word, rare disease supplementary concept word, unique identifier]

10. 5 or 6 or 7 or 8 or 9

11. 4 and 10

12. (random* or blind* or placebo* or meta-analys*).mp. [mp=title, abstract, original title, name of substance word, subject heading word, keyword heading word, protocol supplementary concept word, rare disease supplementary concept word, unique identifier]

13. 11 and 12
