# Supplementary figures and images for: The effects of rhythm control strategies versus rate control strategies for atrial fibrillation and atrial flutter: A systematic review with meta-analysis and Trial Sequential Analysis
Source: PLoS One. 2017 Oct 26;12(10):e0186856. doi: 10.1371/journal.pone.0186856 (PMC5658096; doi:10.1371/journal.pone.0186856)

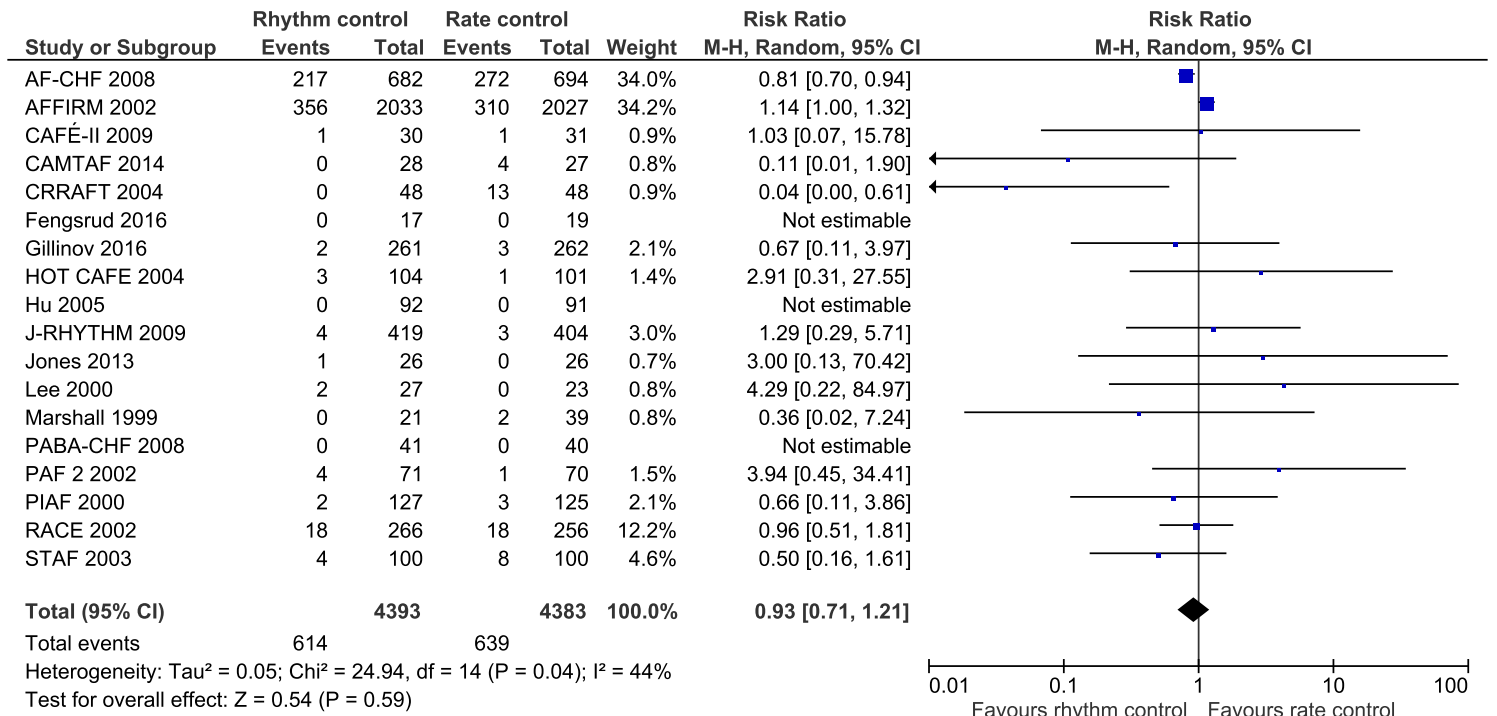

Supplement: S1 Fig — (PDF) [file pone.0186856.s004.pdf]

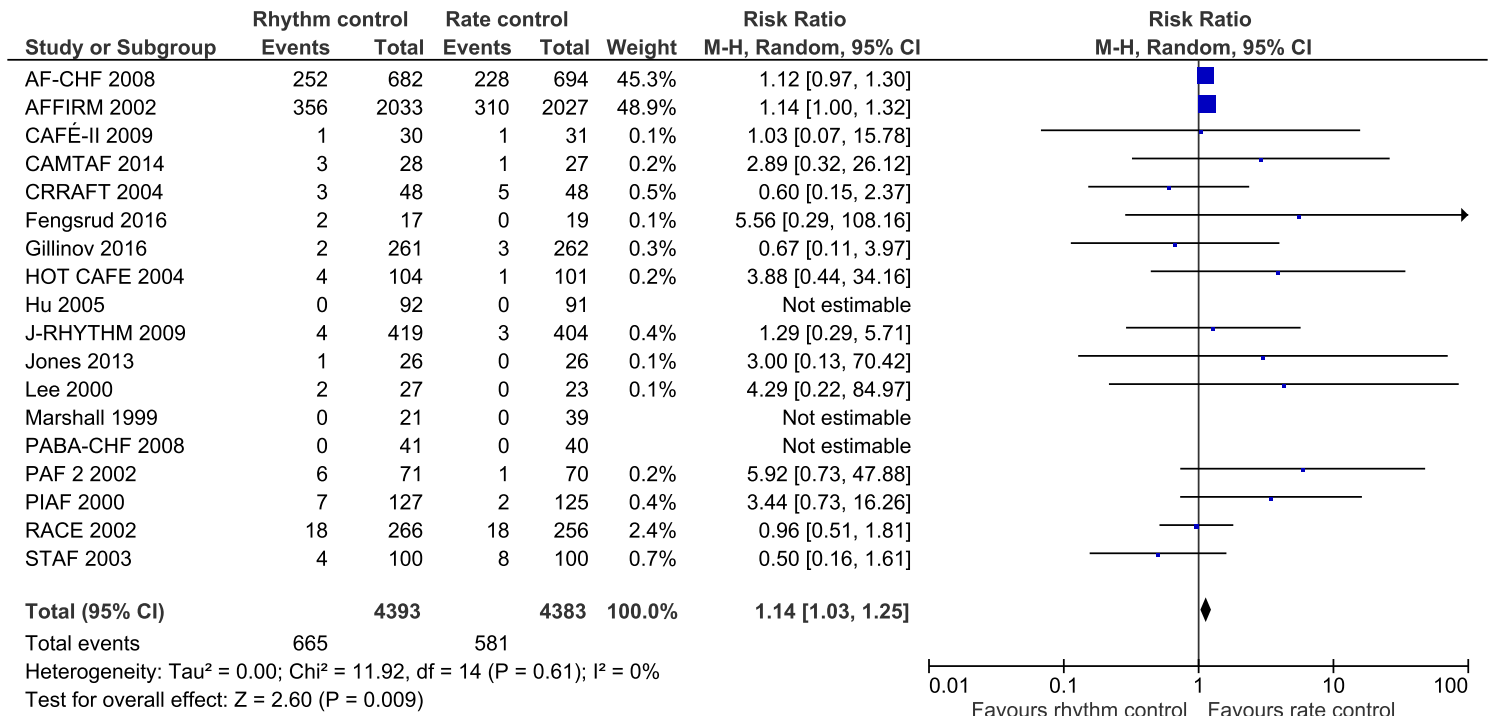

Supplement: S2 Fig — (PDF) [file pone.0186856.s005.pdf]

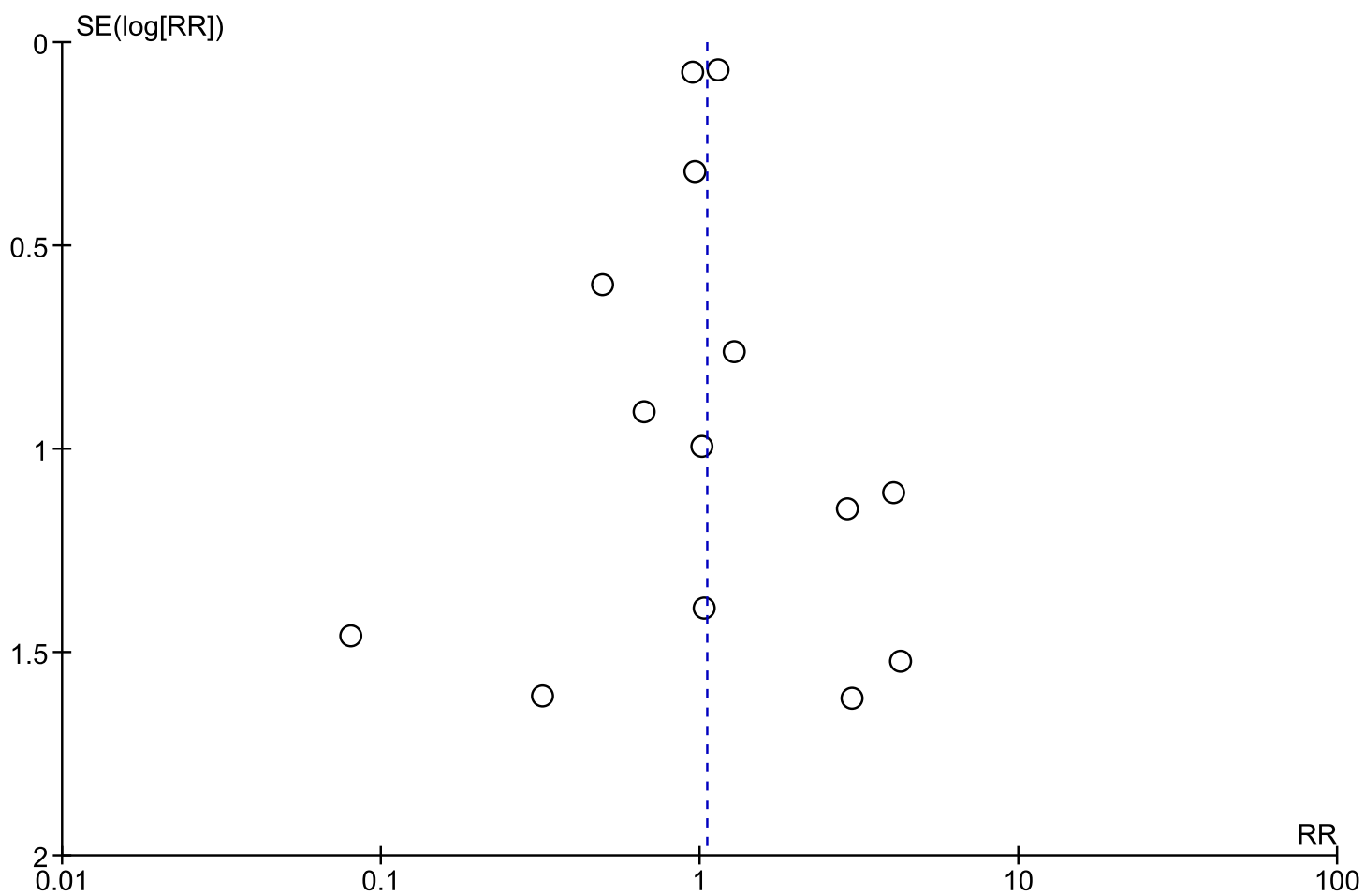

Supplement: S3 Fig — (PDF) [file pone.0186856.s006.pdf]

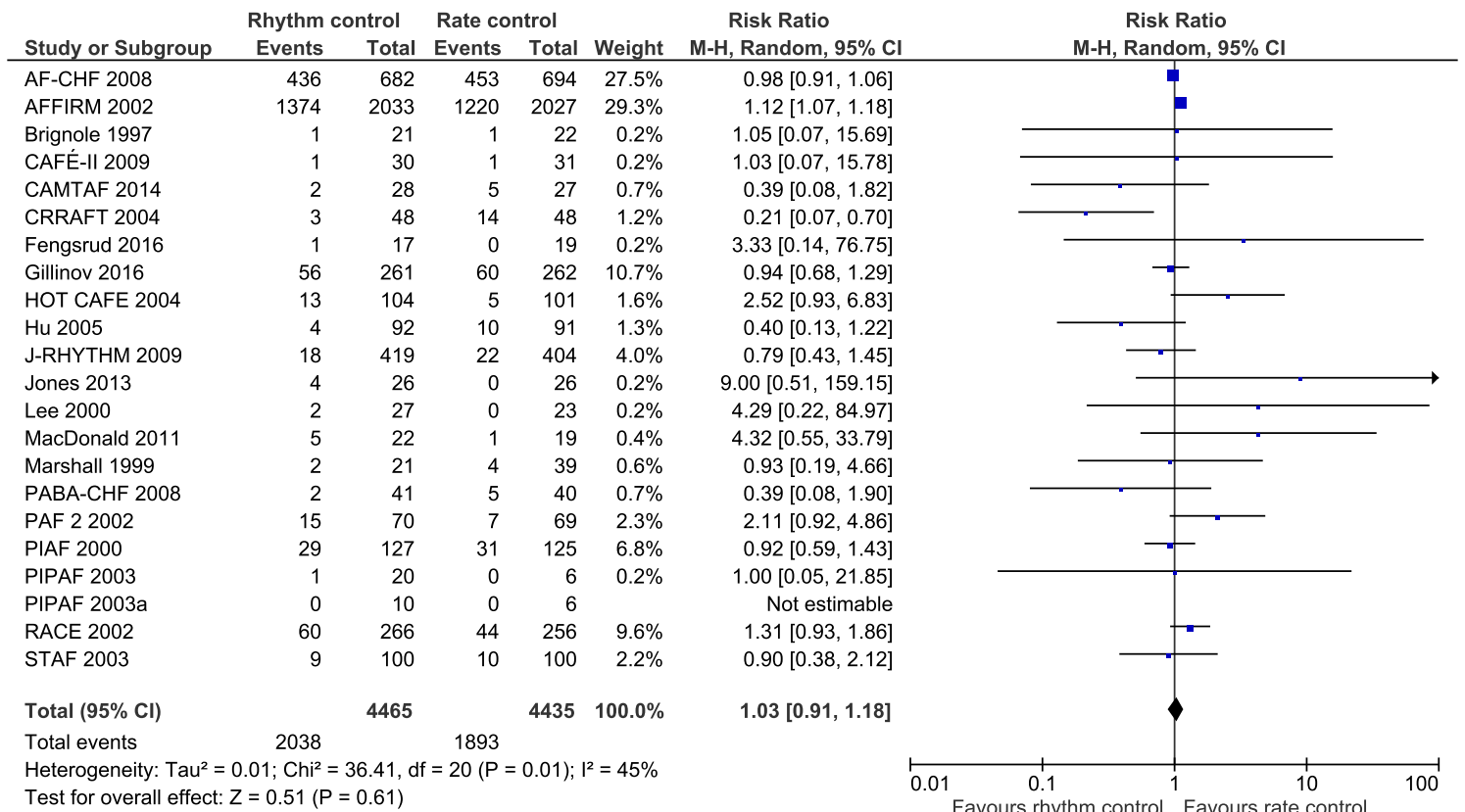

Supplement: S4 Fig — (PDF) [file pone.0186856.s007.pdf]

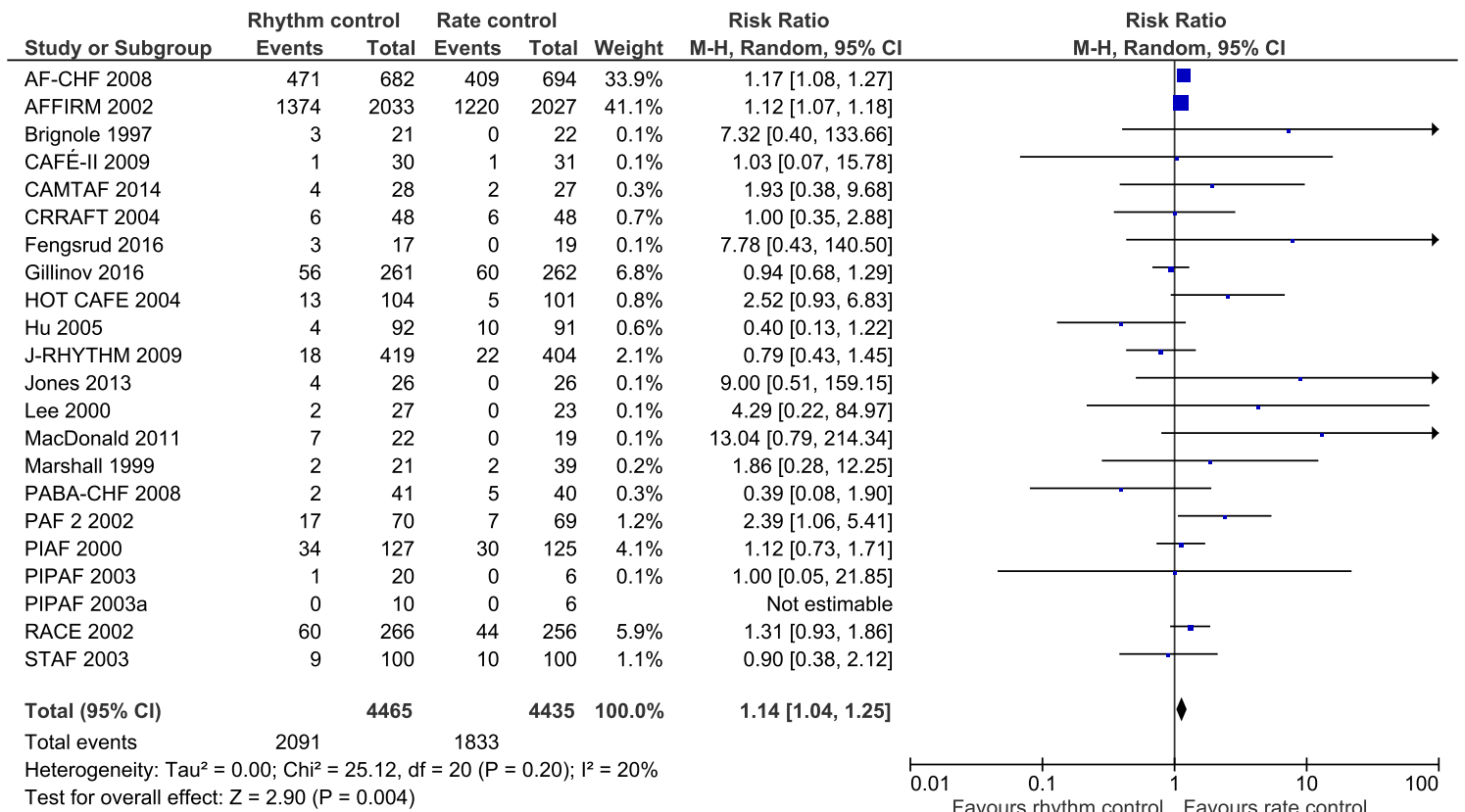

Supplement: S5 Fig — (PDF) [file pone.0186856.s008.pdf]

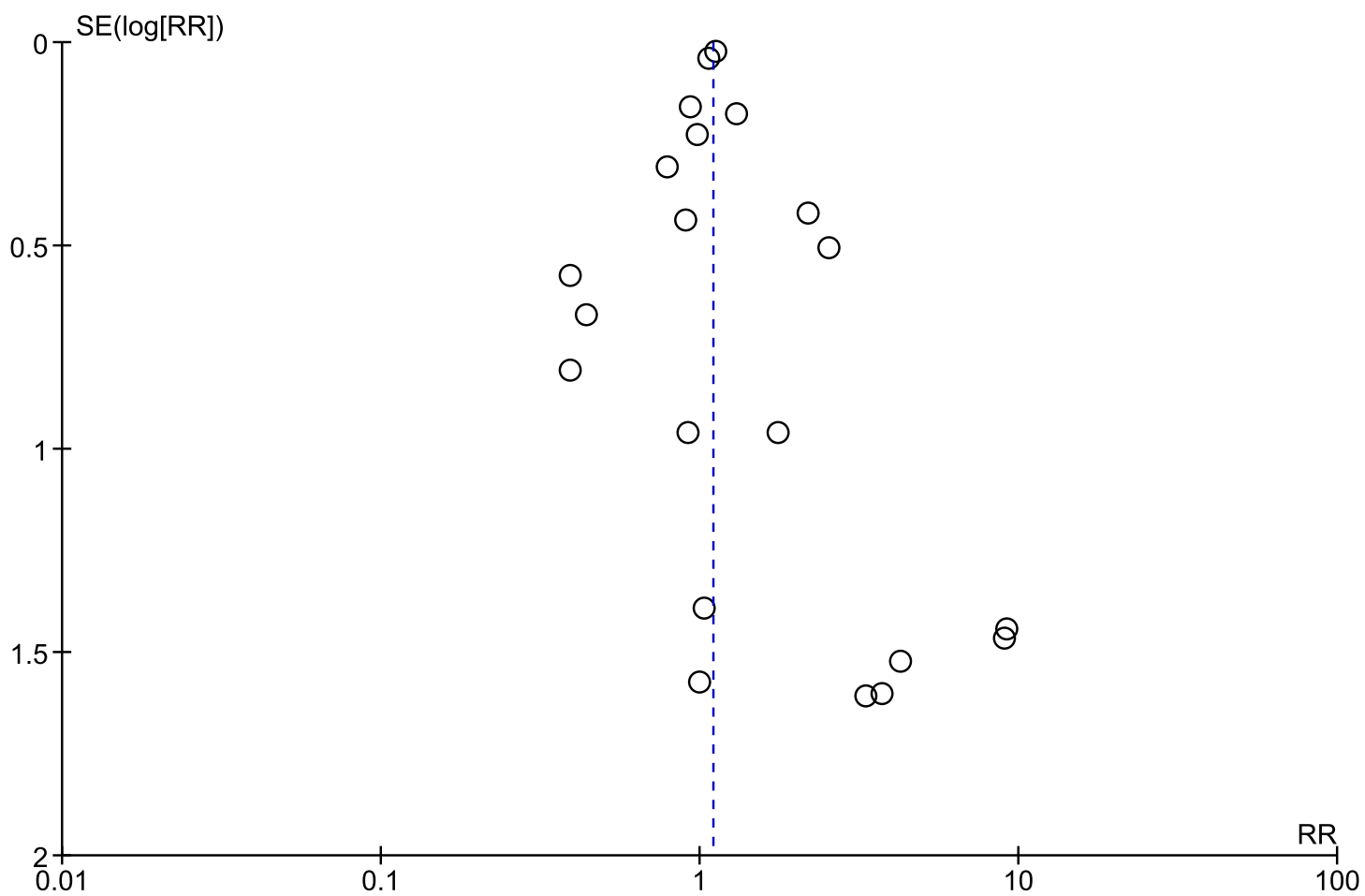

Supplement: S6 Fig — (PDF) [file pone.0186856.s009.pdf]

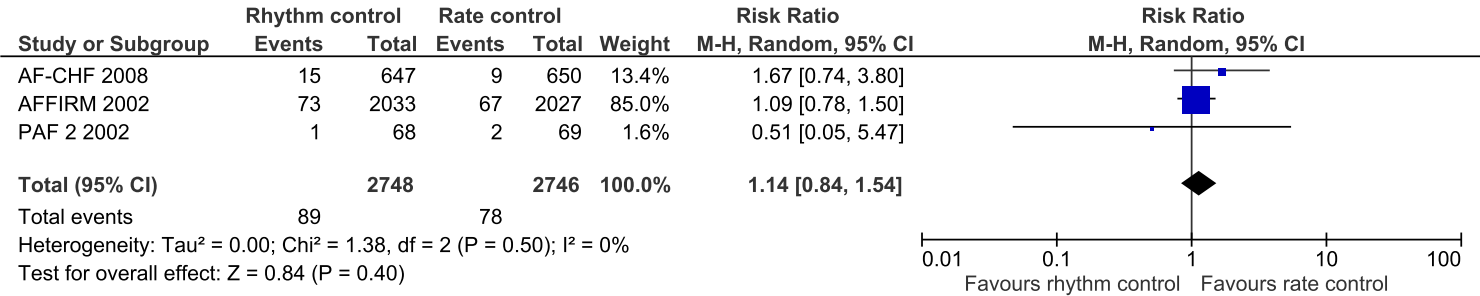

Supplement: S7 Fig — (PDF) [file pone.0186856.s010.pdf]

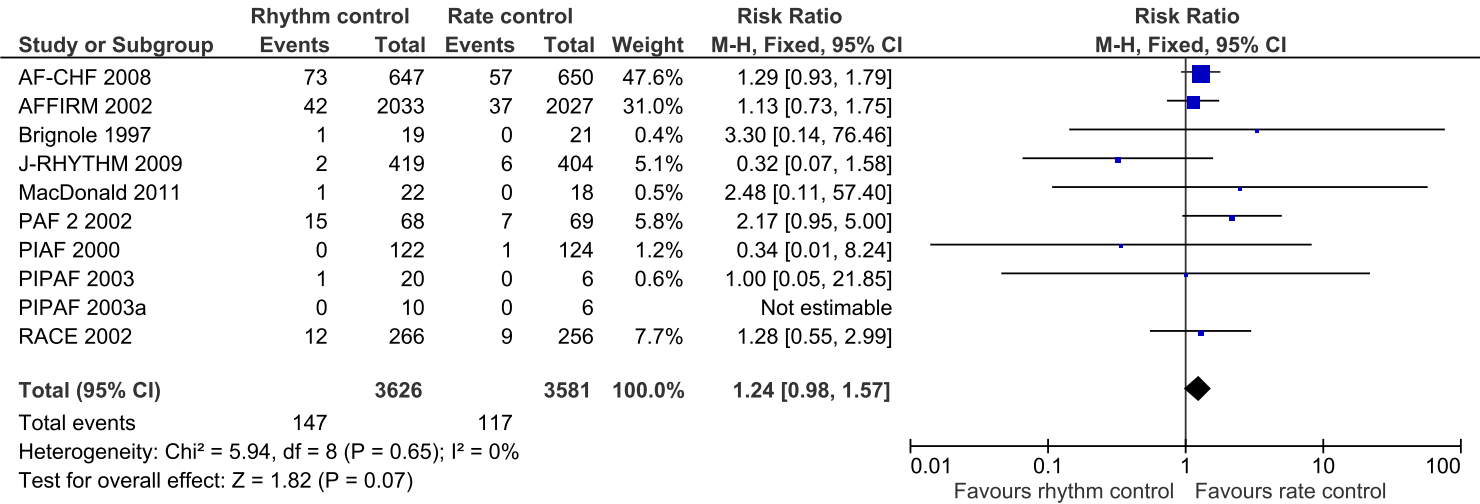

Supplement: S8 Fig — (PDF) [file pone.0186856.s011.pdf]

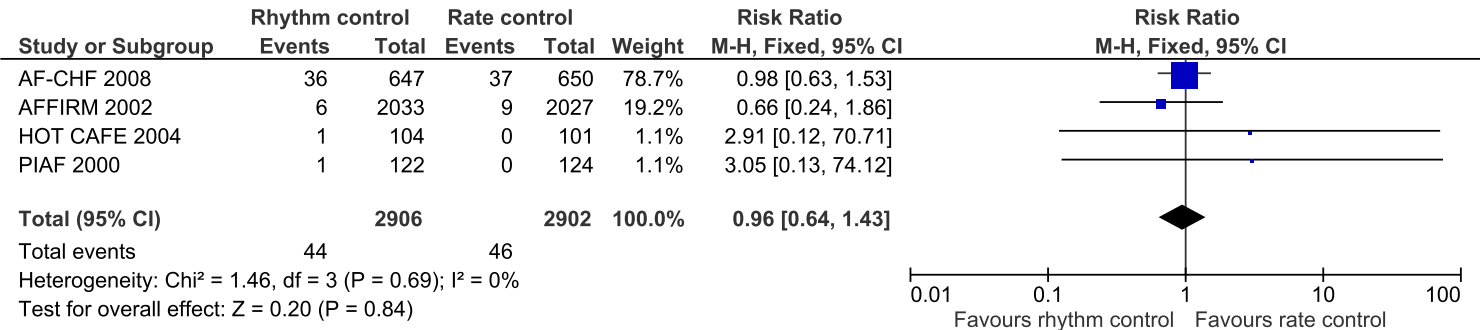

Supplement: S9 Fig — (PDF) [file pone.0186856.s012.pdf]

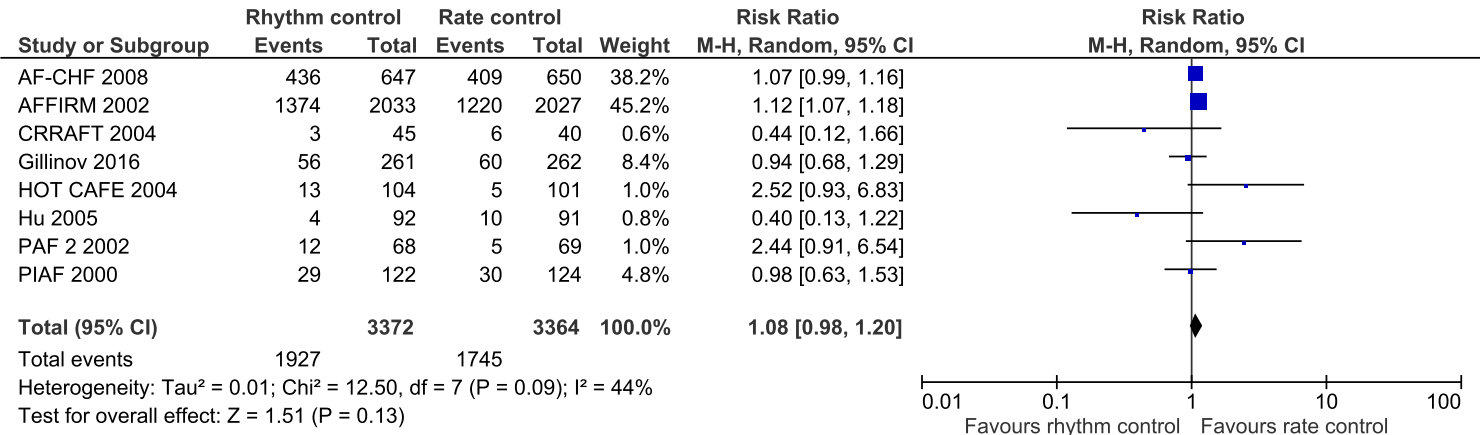

Supplement: S10 Fig — (PDF) [file pone.0186856.s013.pdf]

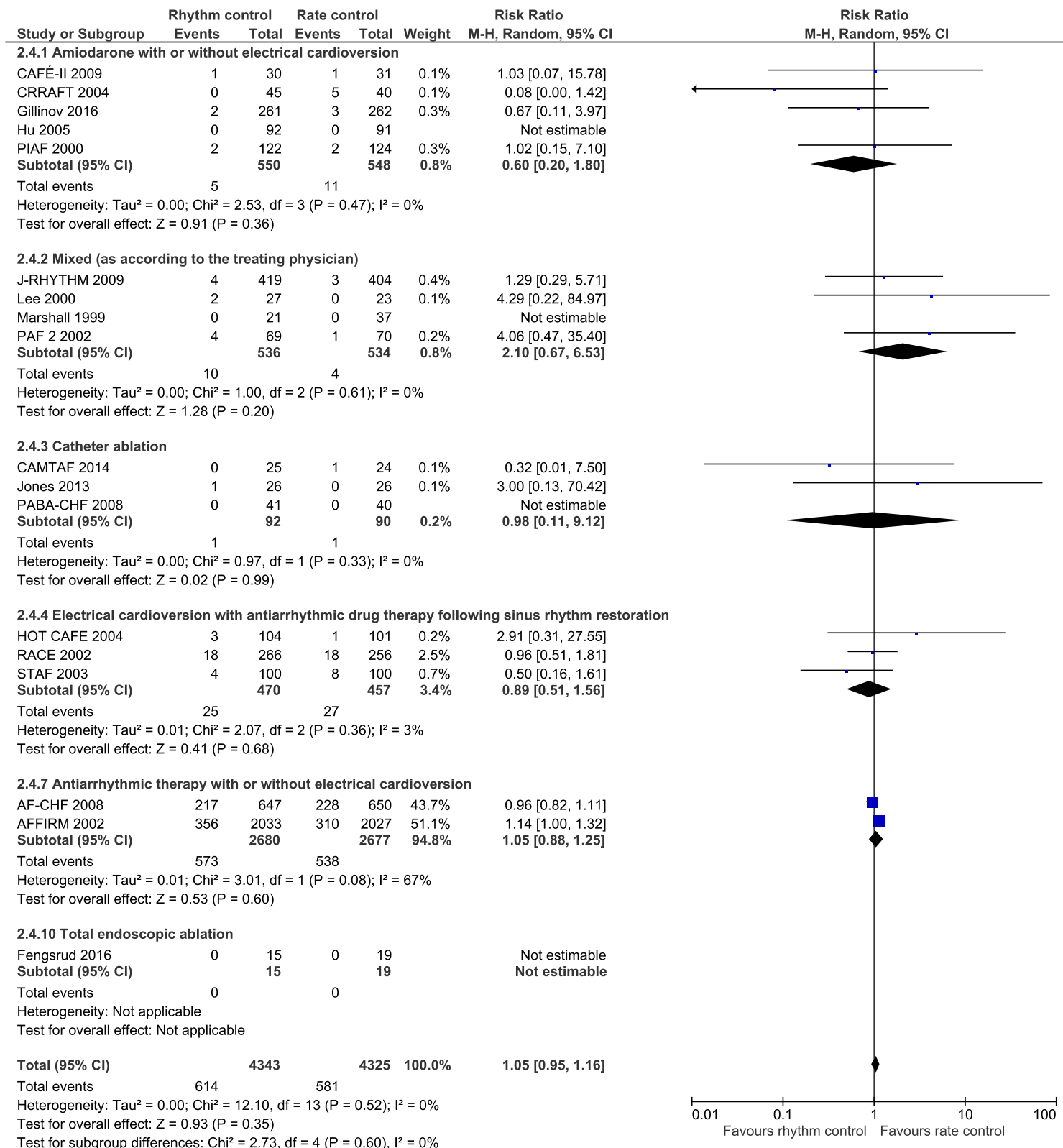

Supplement: S11 Fig — (PDF) [file pone.0186856.s014.pdf]

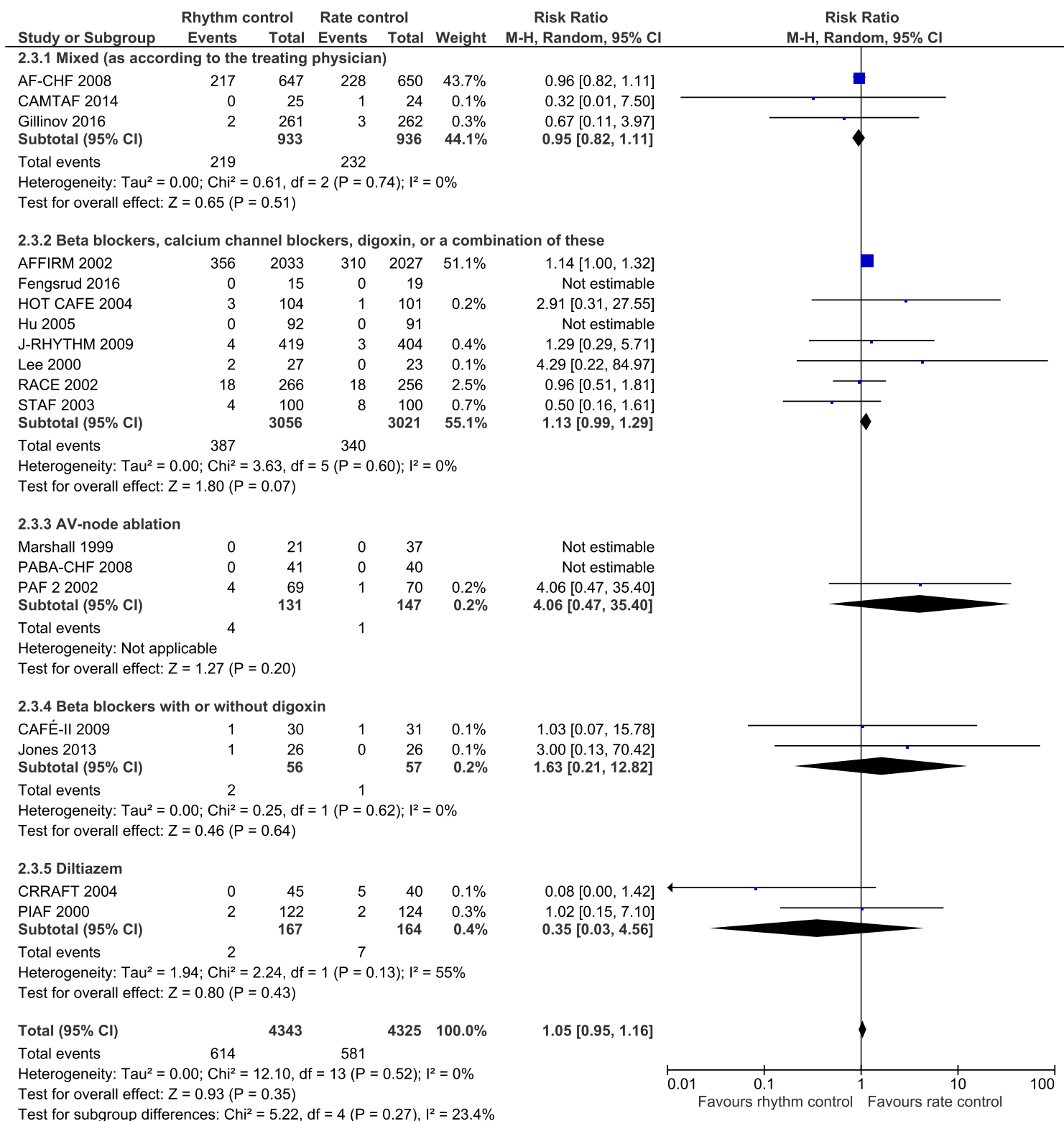

Supplement: S12 Fig — (PDF) [file pone.0186856.s015.pdf]

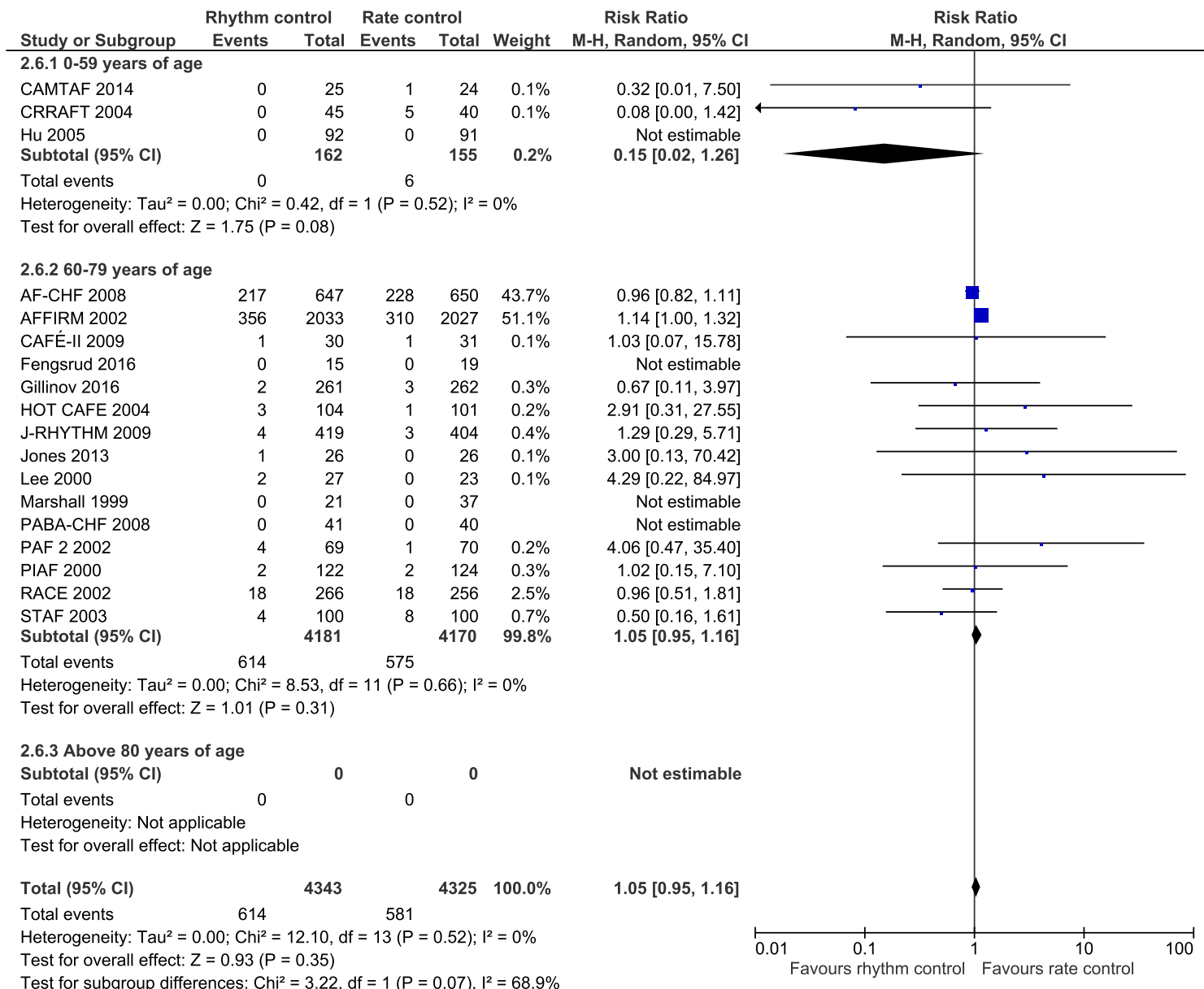

Supplement: S13 Fig — (PDF) [file pone.0186856.s016.pdf]

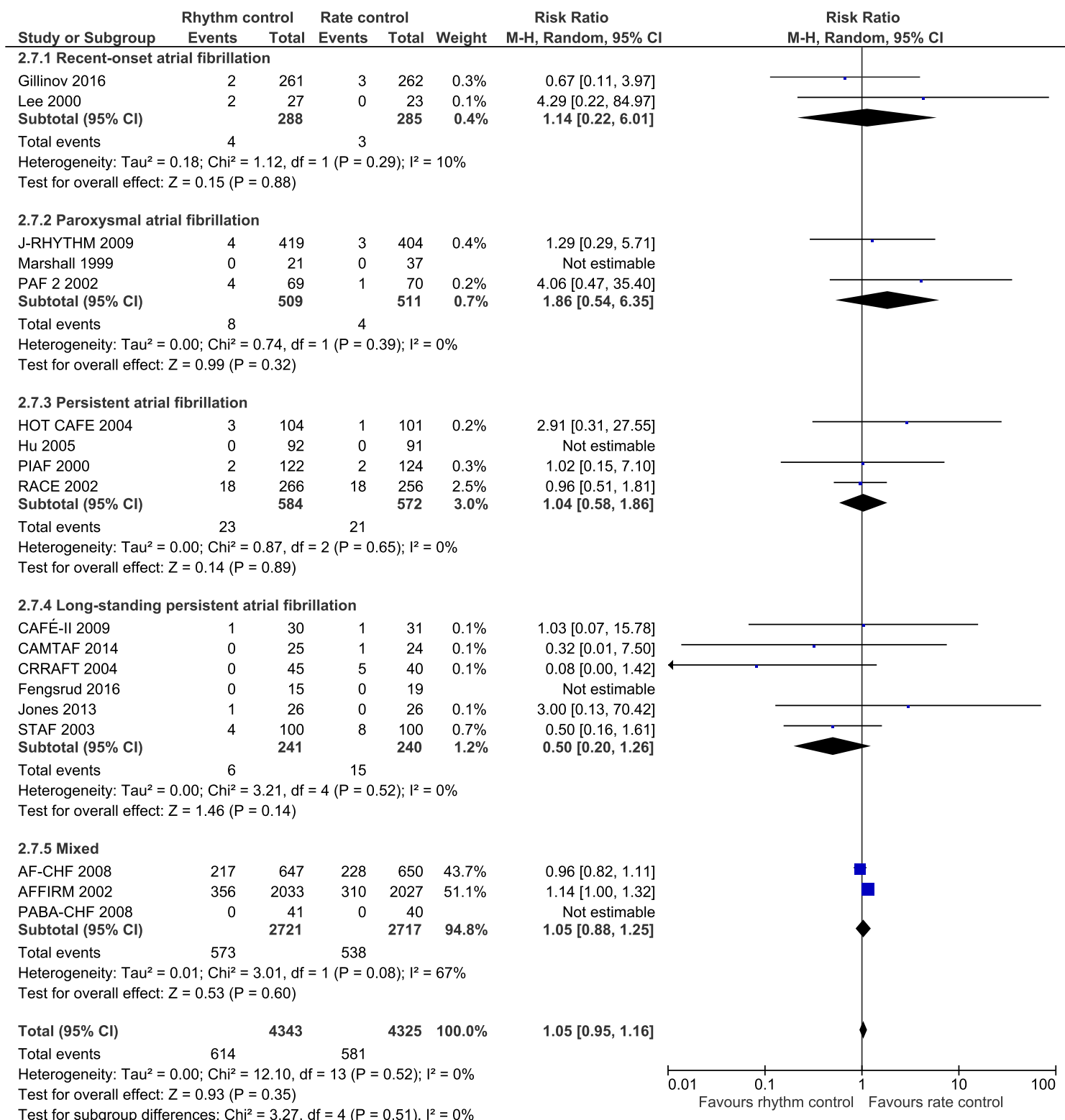

Supplement: S14 Fig — (PDF) [file pone.0186856.s017.pdf]

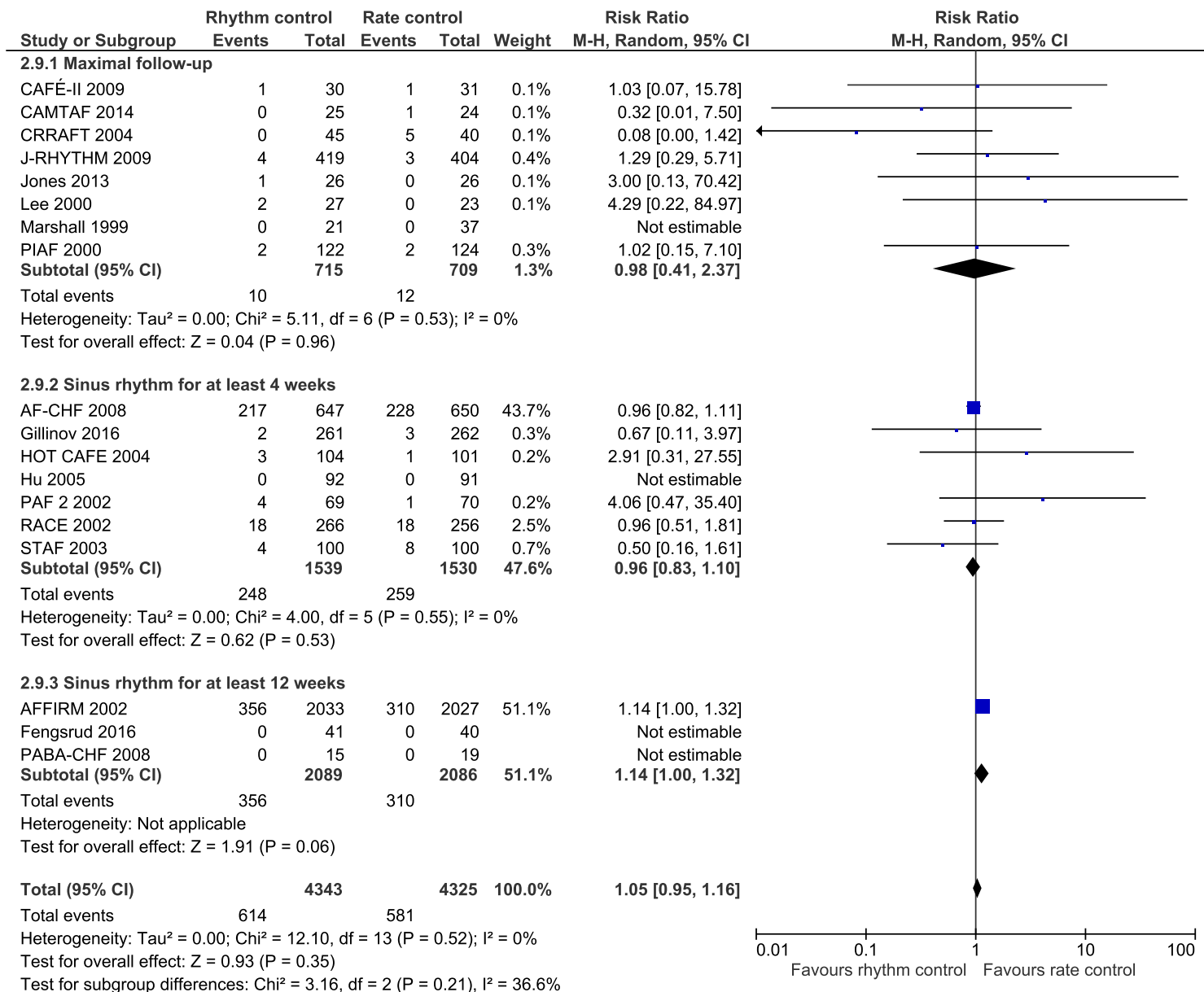

Supplement: S15 Fig — (PDF) [file pone.0186856.s018.pdf]

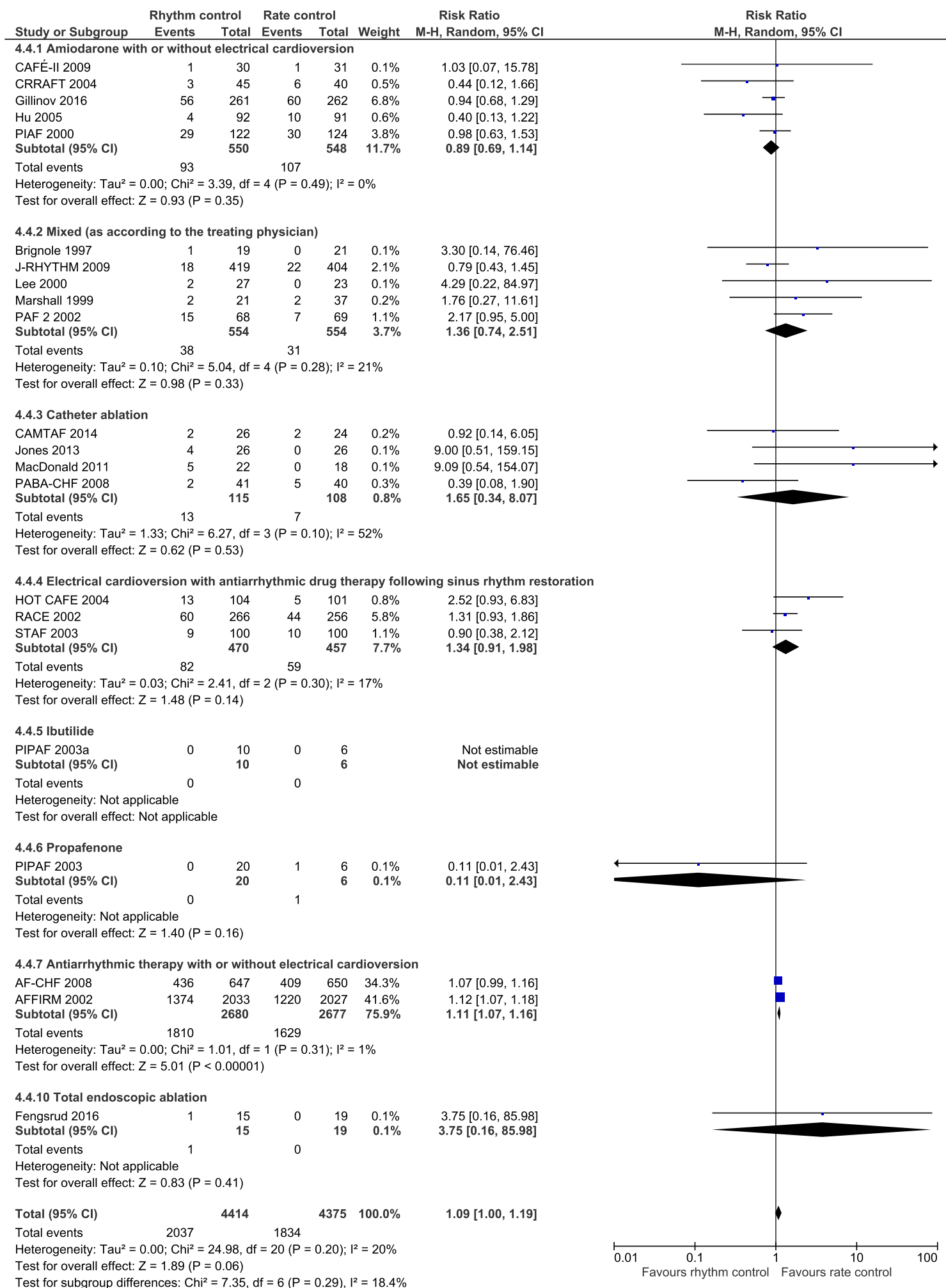

Supplement: S16 Fig — (PDF) [file pone.0186856.s019.pdf]

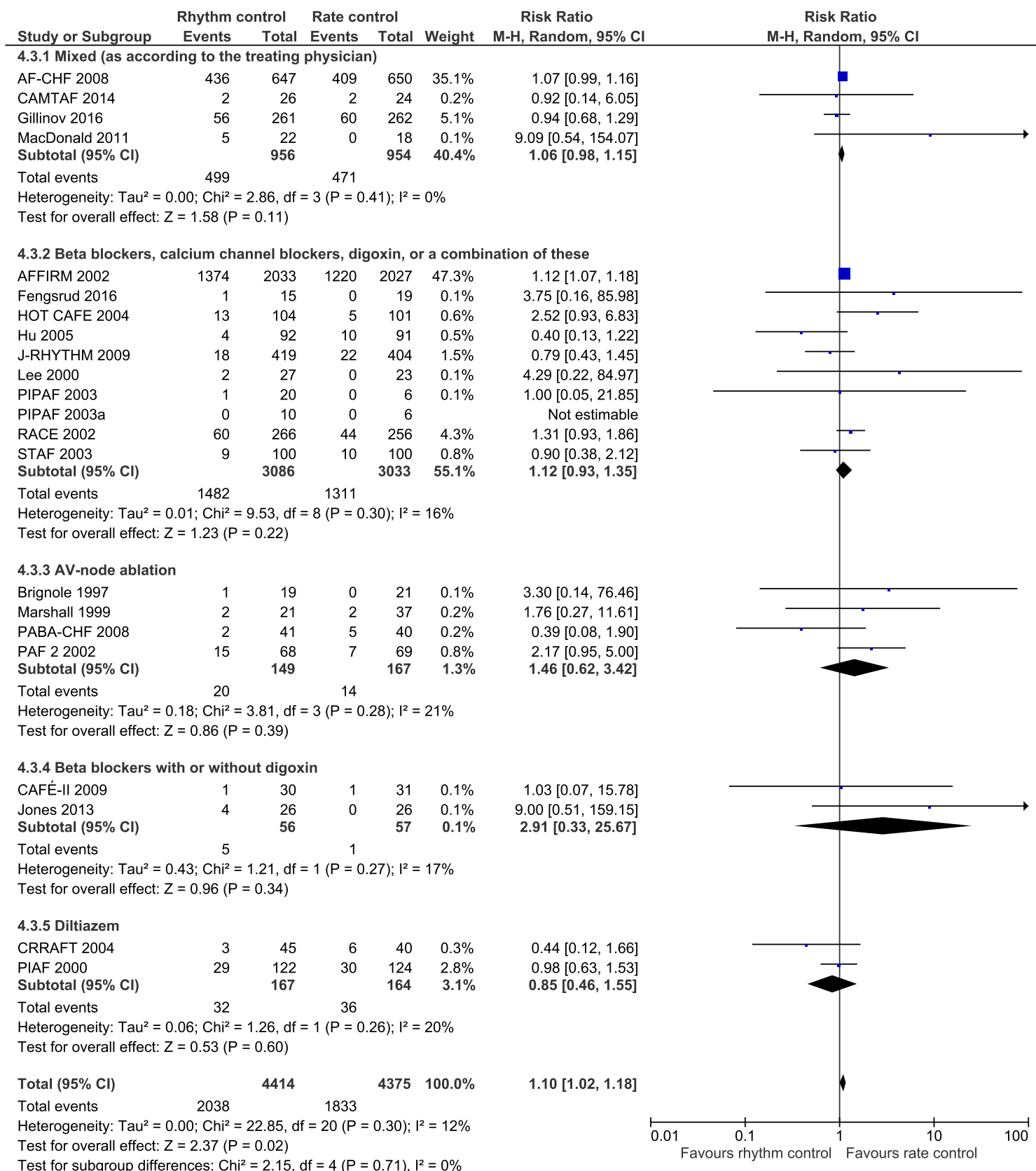

Supplement: S17 Fig — (PDF) [file pone.0186856.s020.pdf]

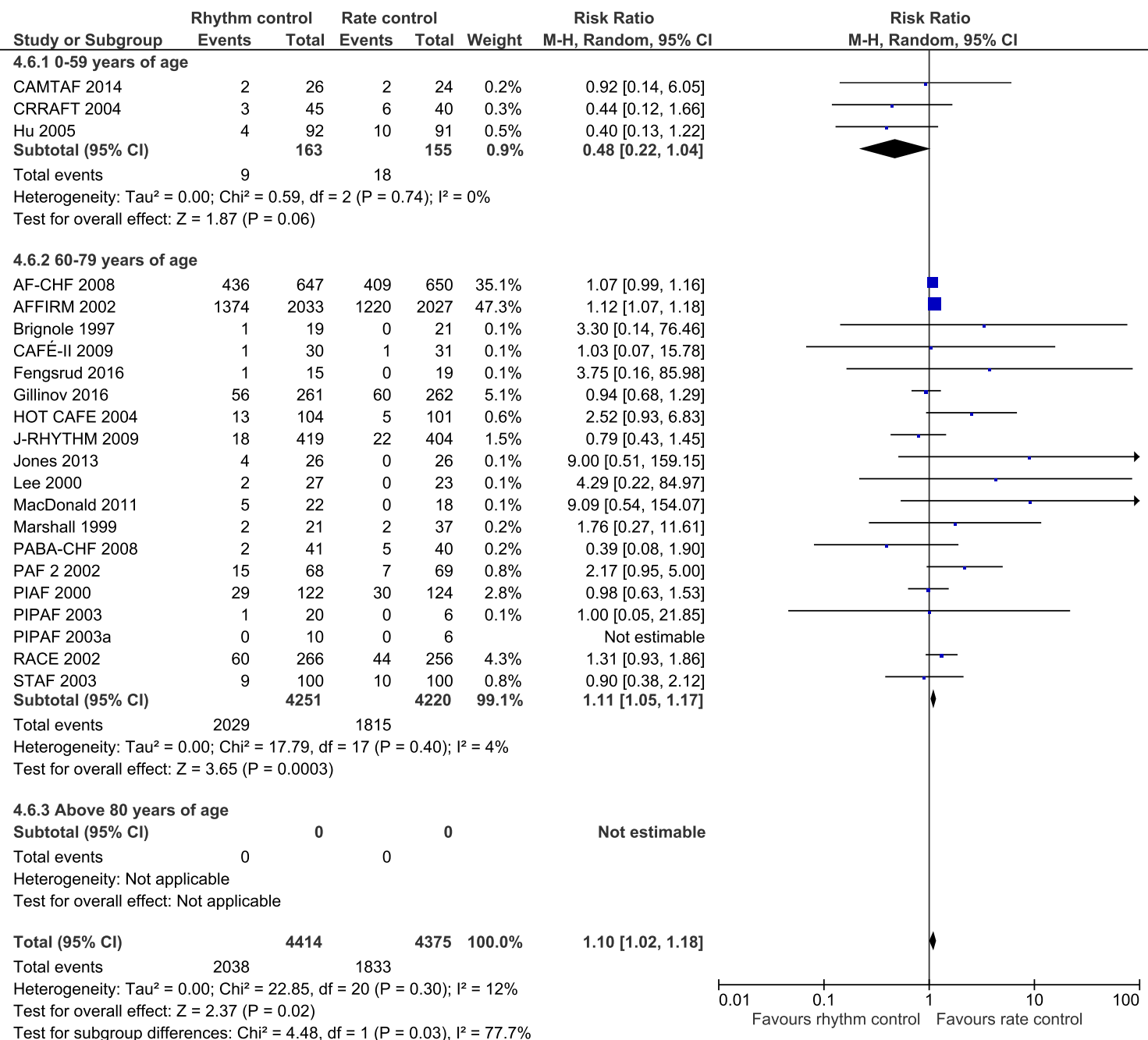

Supplement: S18 Fig — (PDF) [file pone.0186856.s021.pdf]

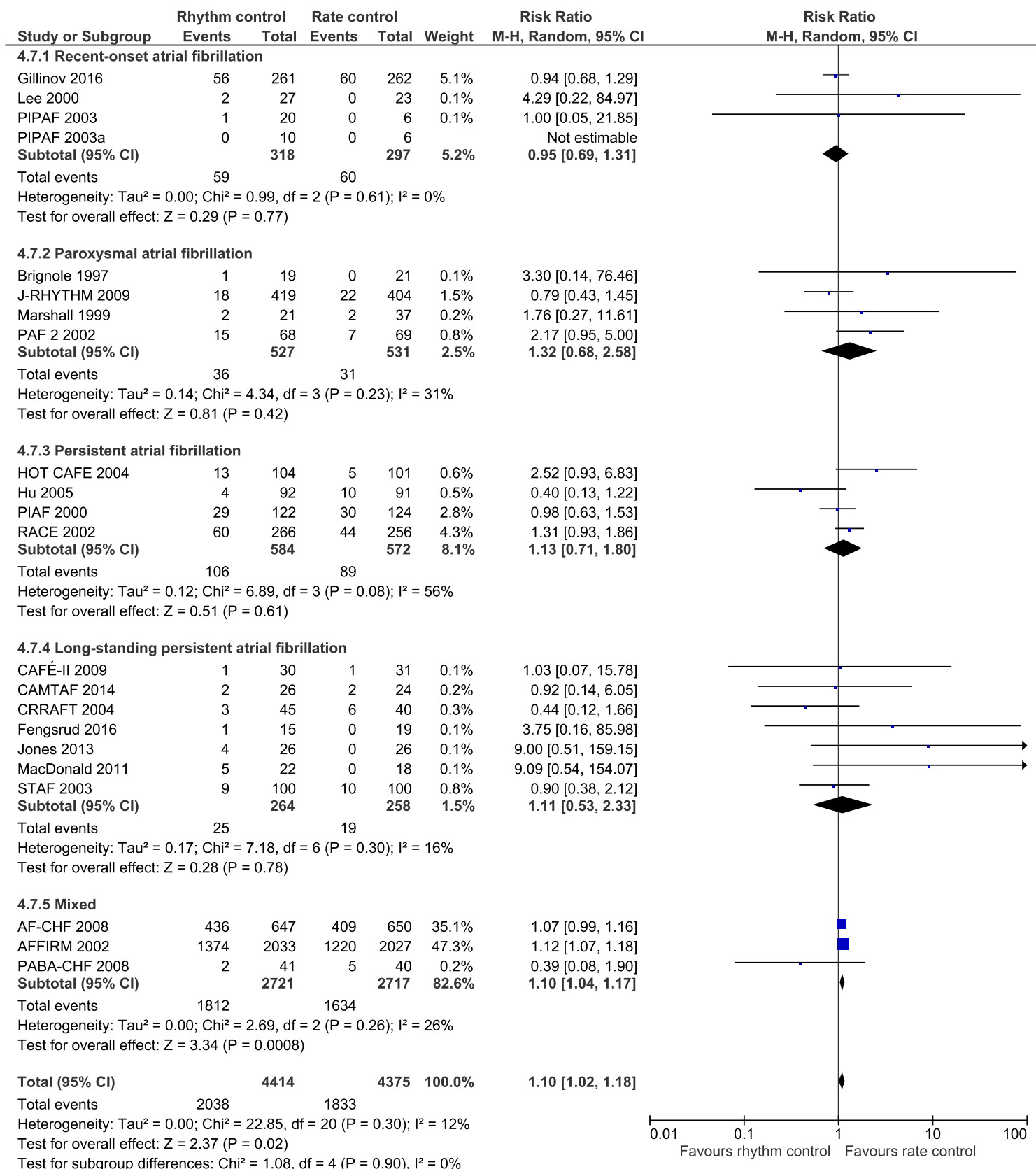

Supplement: S19 Fig — (PDF) [file pone.0186856.s022.pdf]

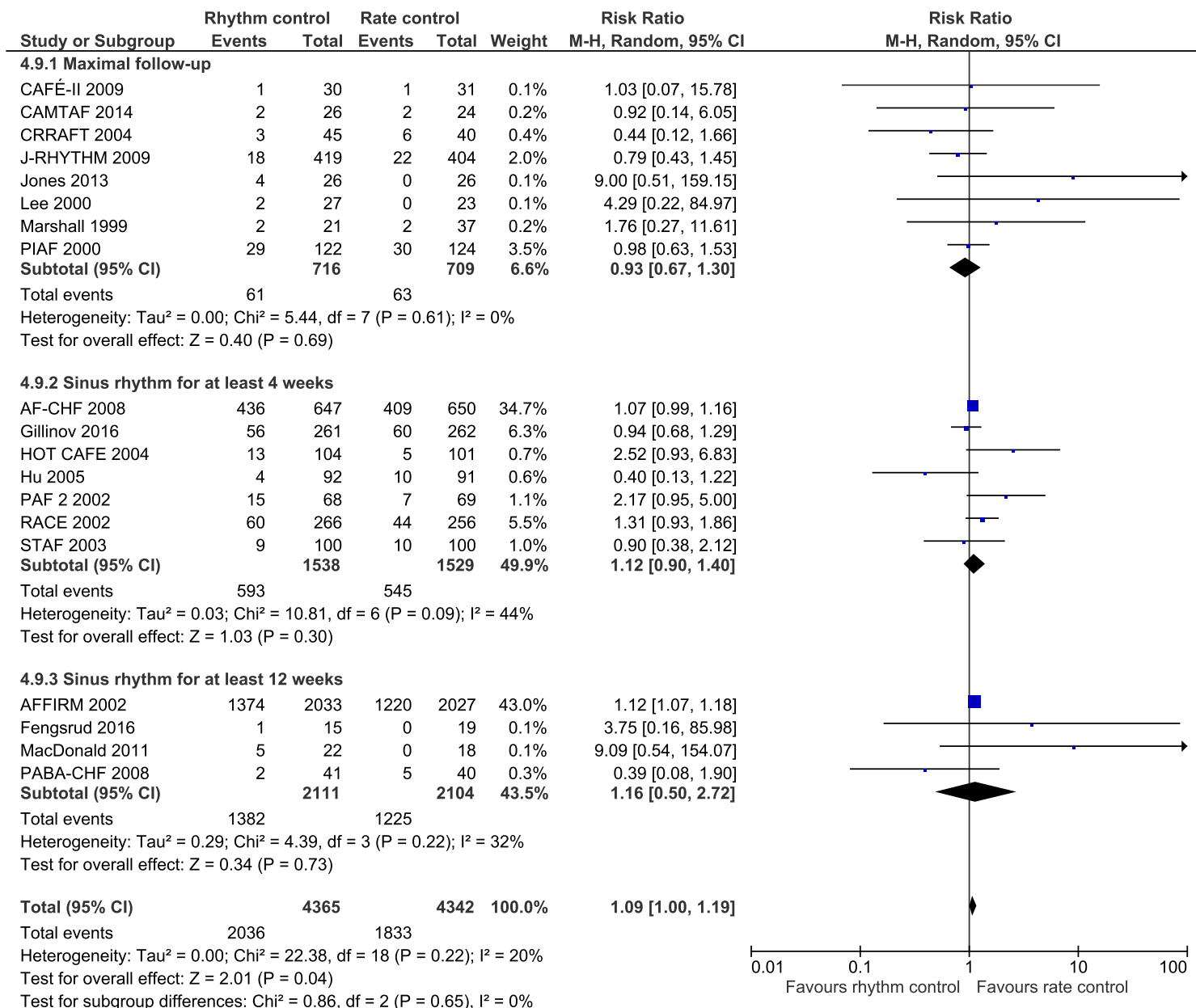

Supplement: S20 Fig — (PDF) [file pone.0186856.s023.pdf]

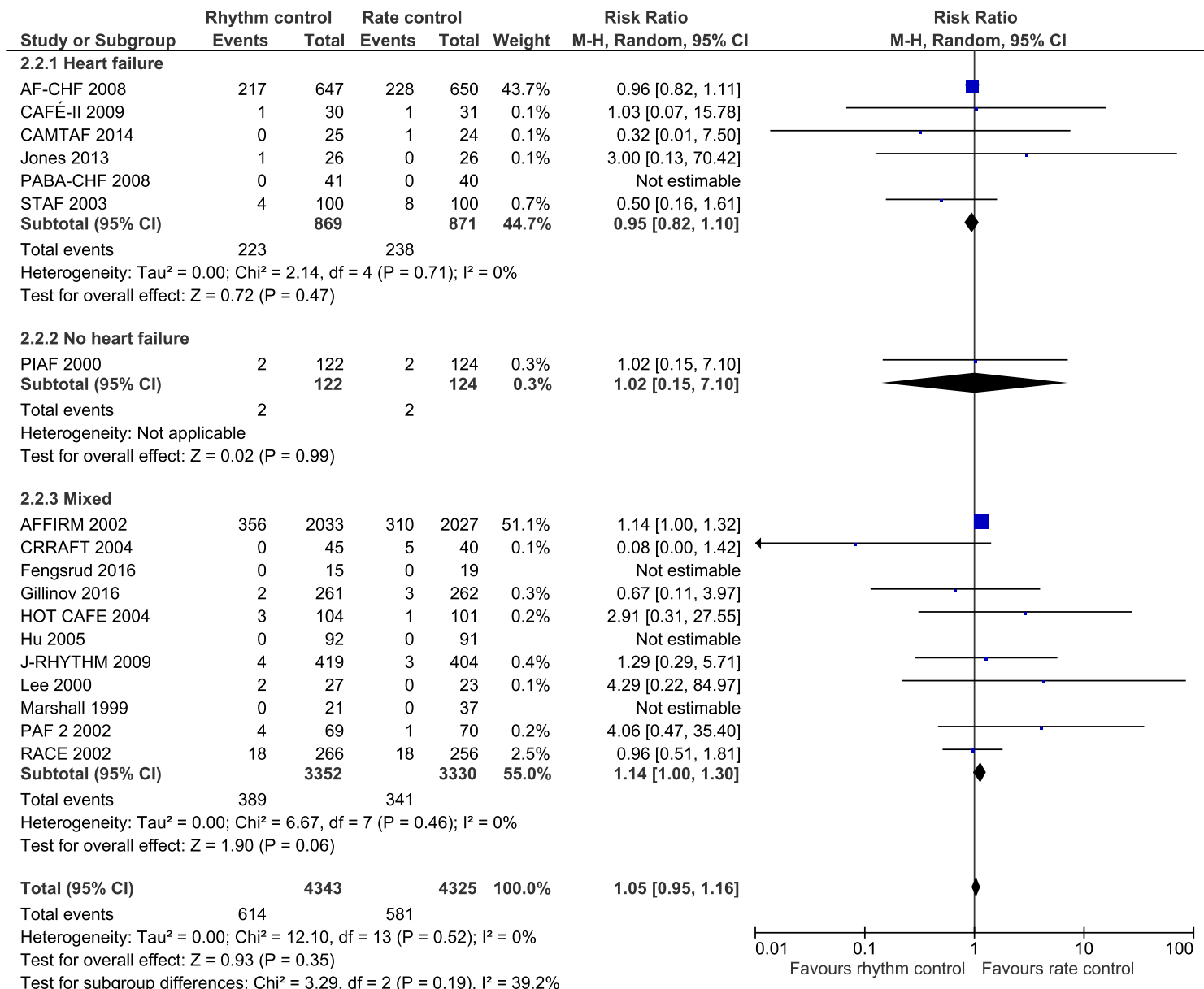

Supplement: S21 Fig — (PDF) [file pone.0186856.s024.pdf]

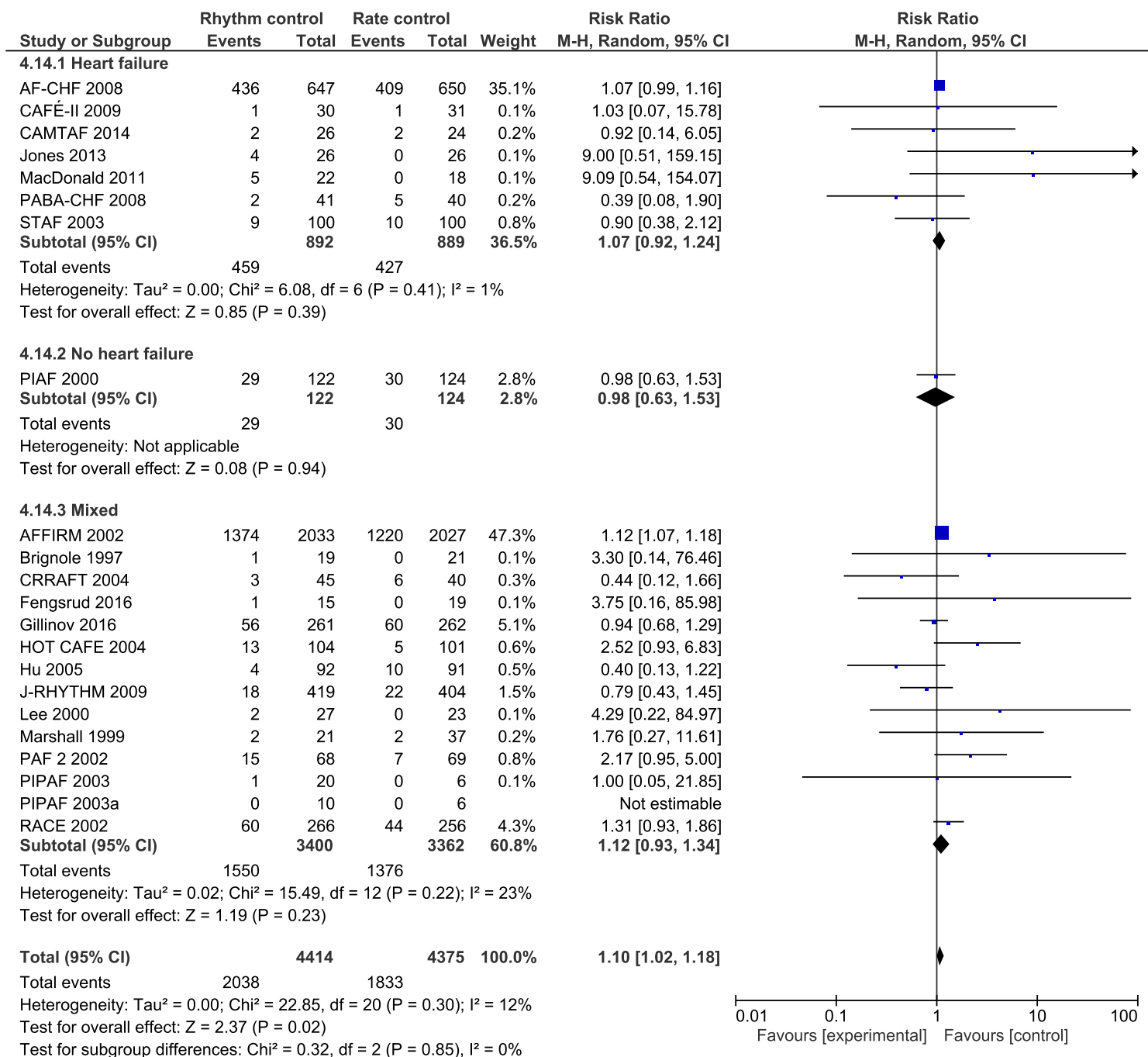

Supplement: S22 Fig — (PDF) [file pone.0186856.s025.pdf]

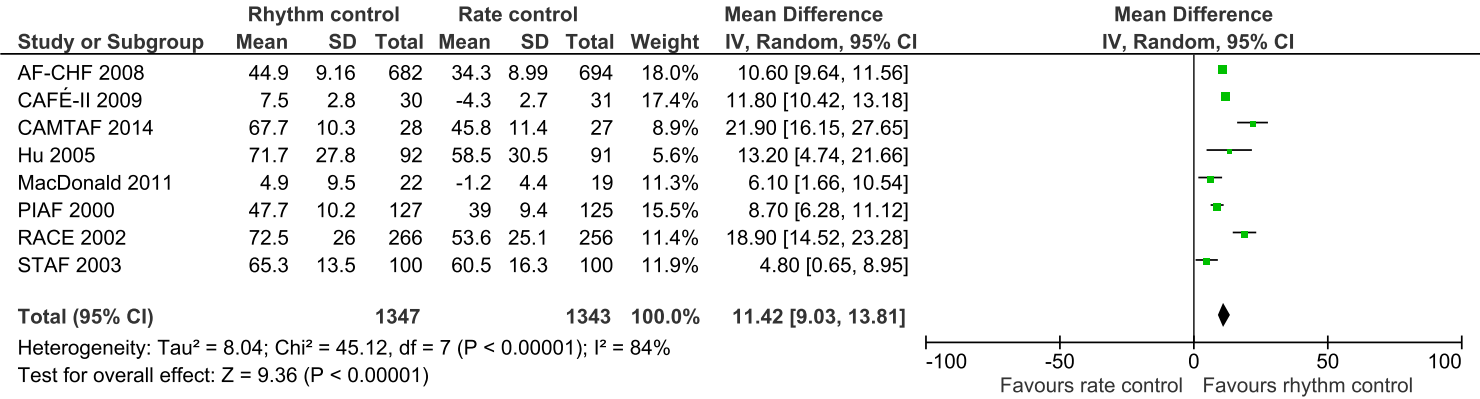

Supplement: S23 Fig — (PDF) [file pone.0186856.s026.pdf]

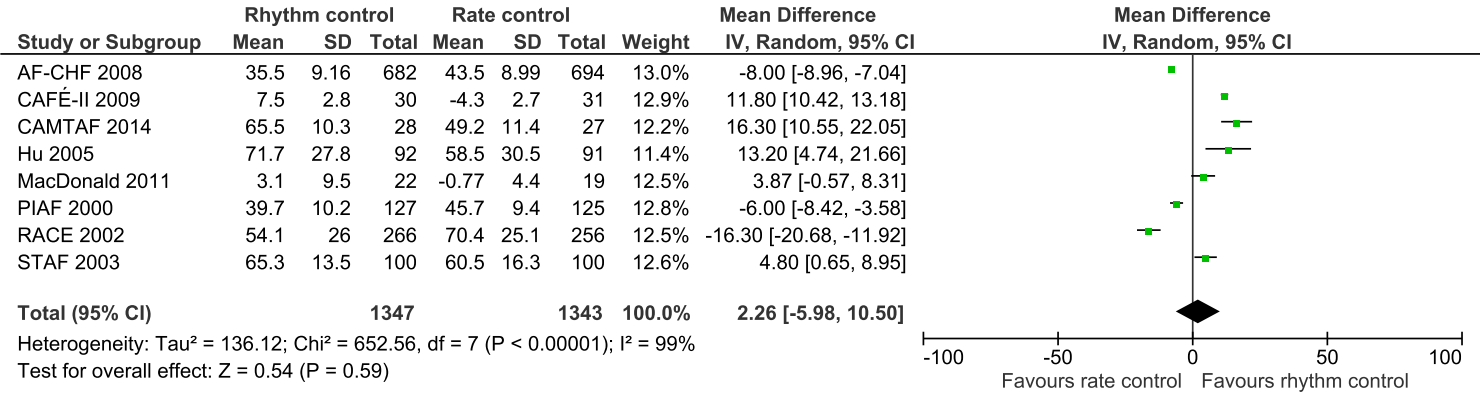

Supplement: S24 Fig — (PDF) [file pone.0186856.s027.pdf]

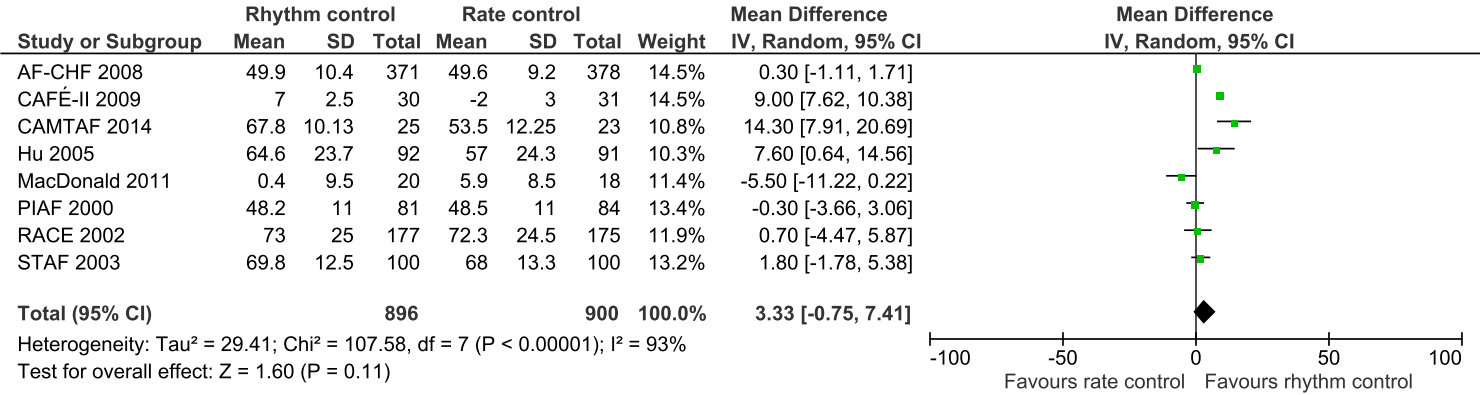

Supplement: S25 Fig — (PDF) [file pone.0186856.s028.pdf]

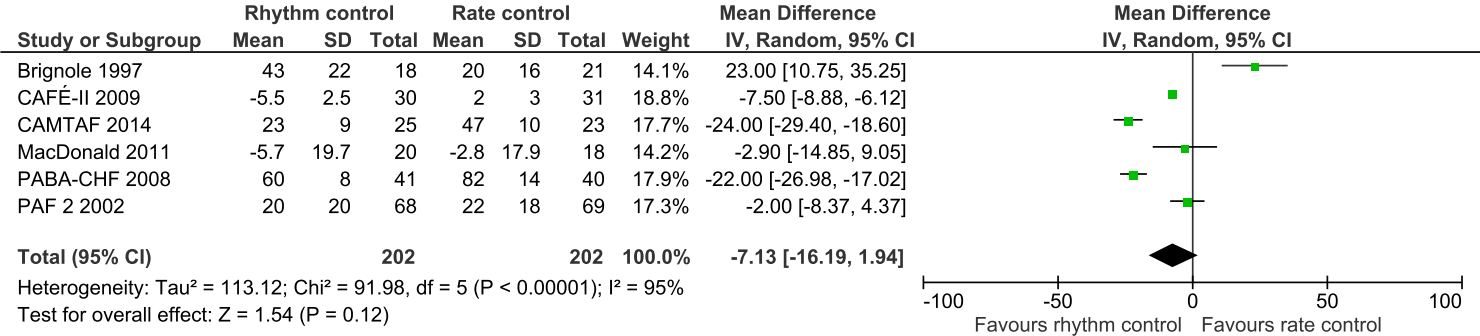

Supplement: S26 Fig — (PDF) [file pone.0186856.s029.pdf]

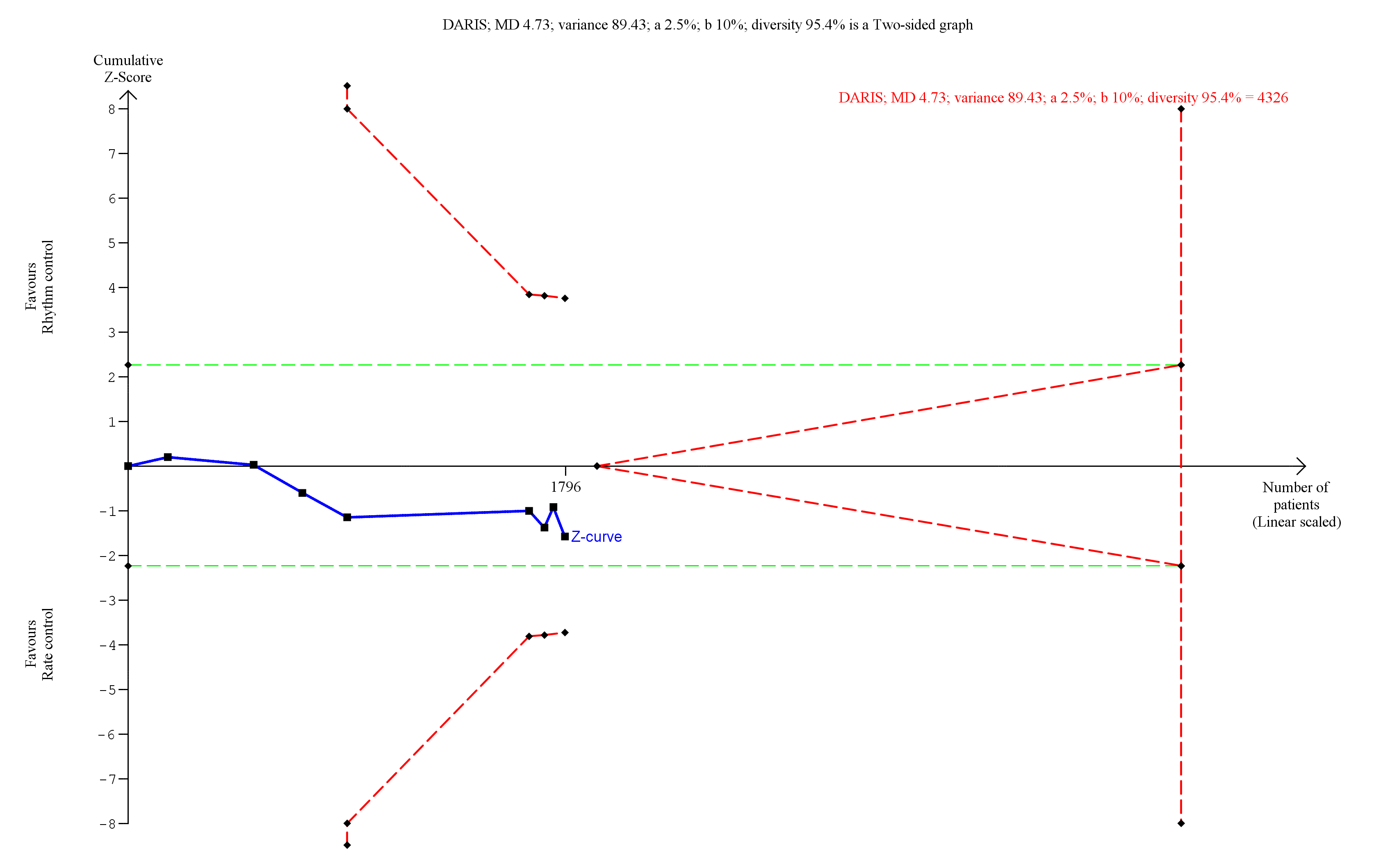

Supplement: S27 Fig — (PNG) [file pone.0186856.s030.png]

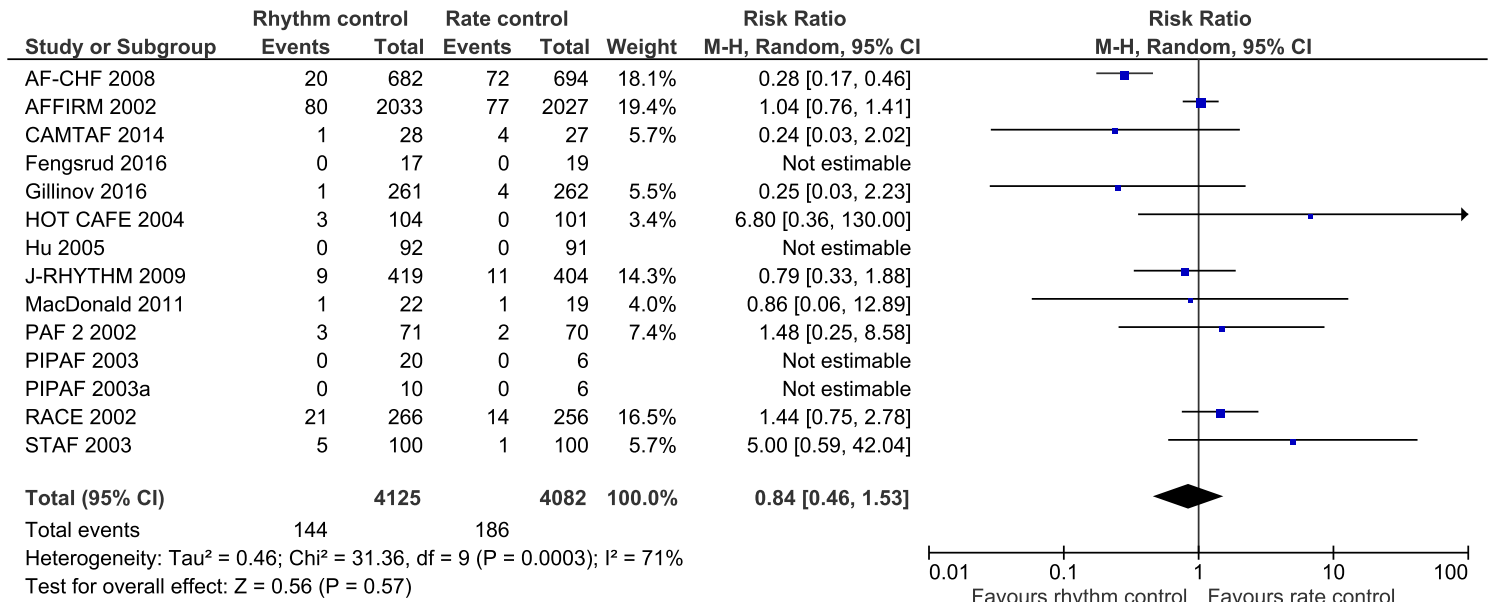

Supplement: S28 Fig — (PDF) [file pone.0186856.s031.pdf]

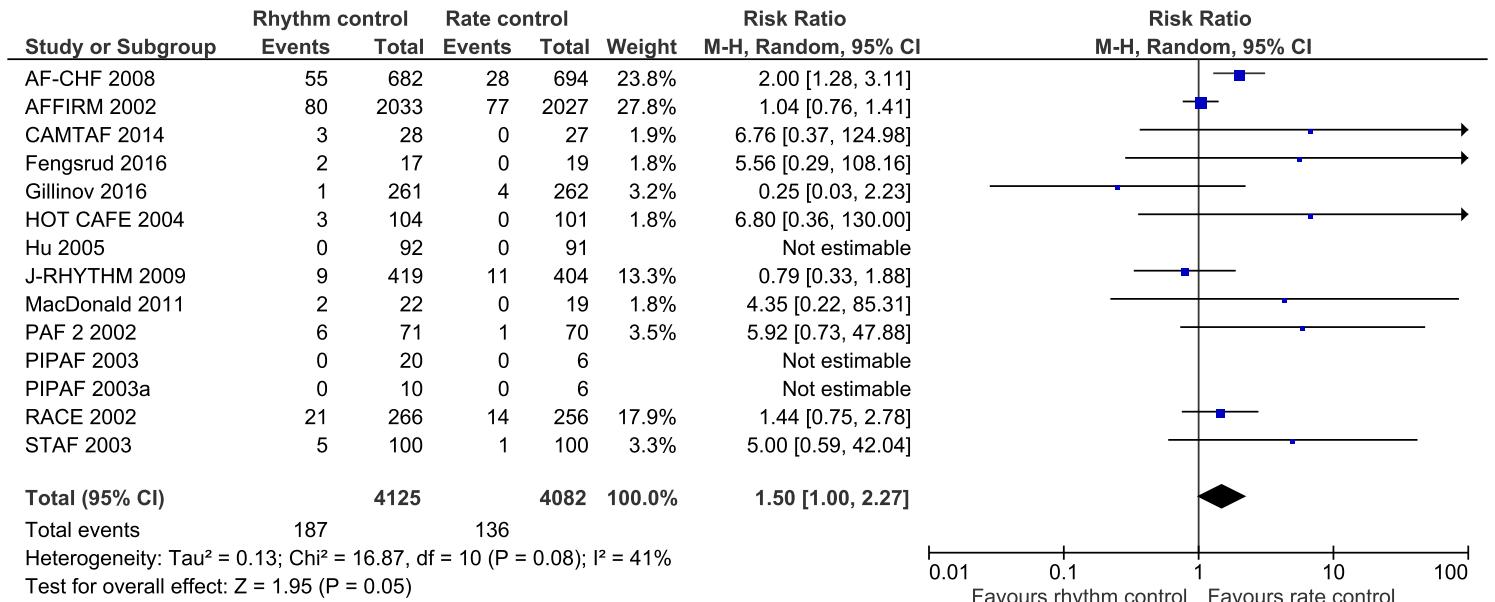

Supplement: S29 Fig — (PDF) [file pone.0186856.s032.pdf]

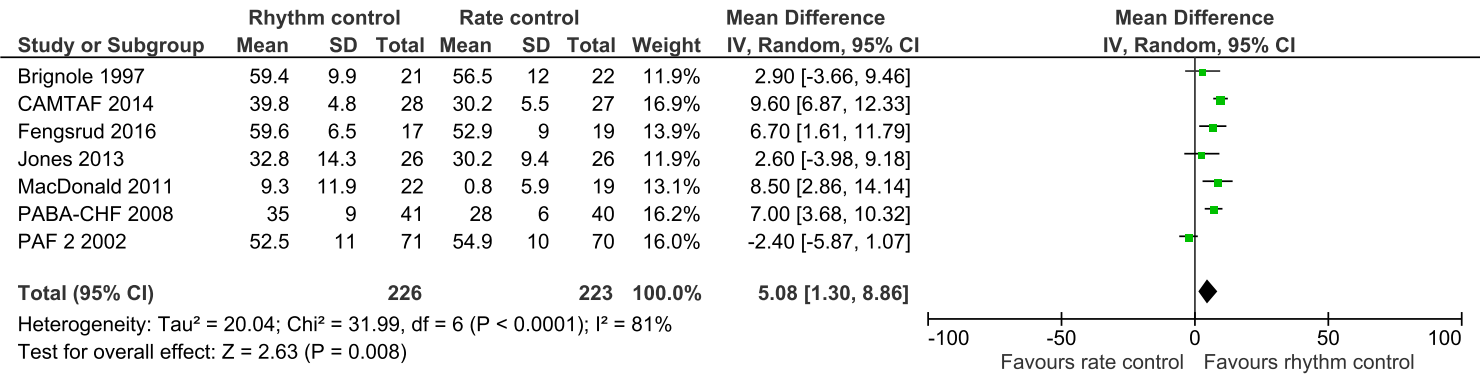

Supplement: S30 Fig — (PDF) [file pone.0186856.s033.pdf]

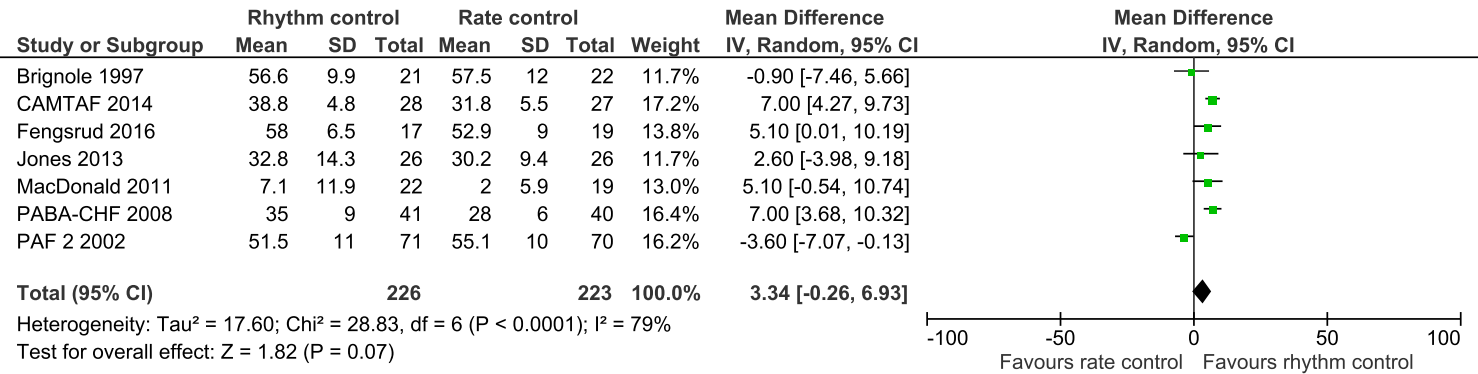

Supplement: S31 Fig — (PDF) [file pone.0186856.s034.pdf]

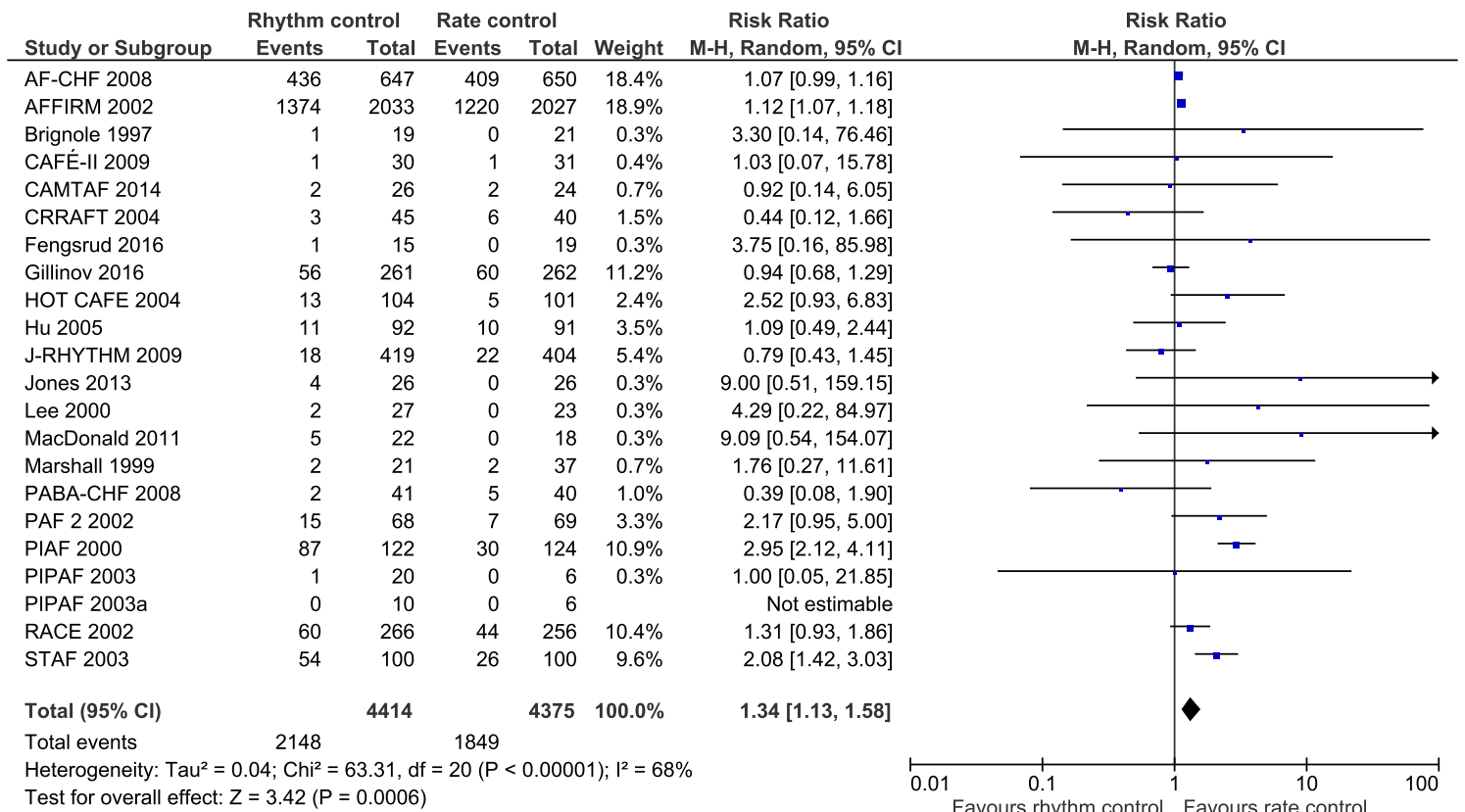

Supplement: S32 Fig — (PDF) [file pone.0186856.s035.pdf]

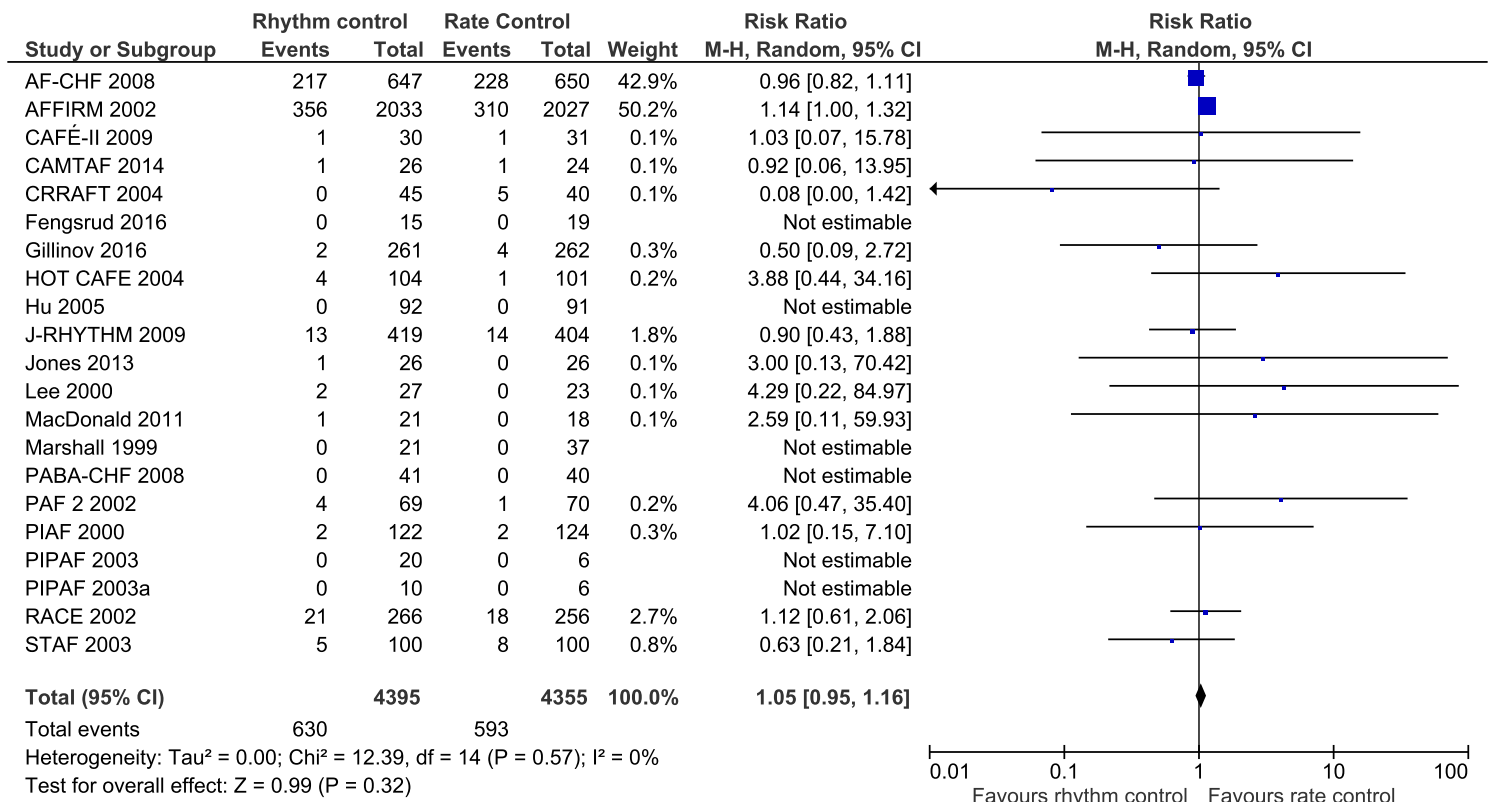

Supplement: S33 Fig — (PDF) [file pone.0186856.s036.pdf]

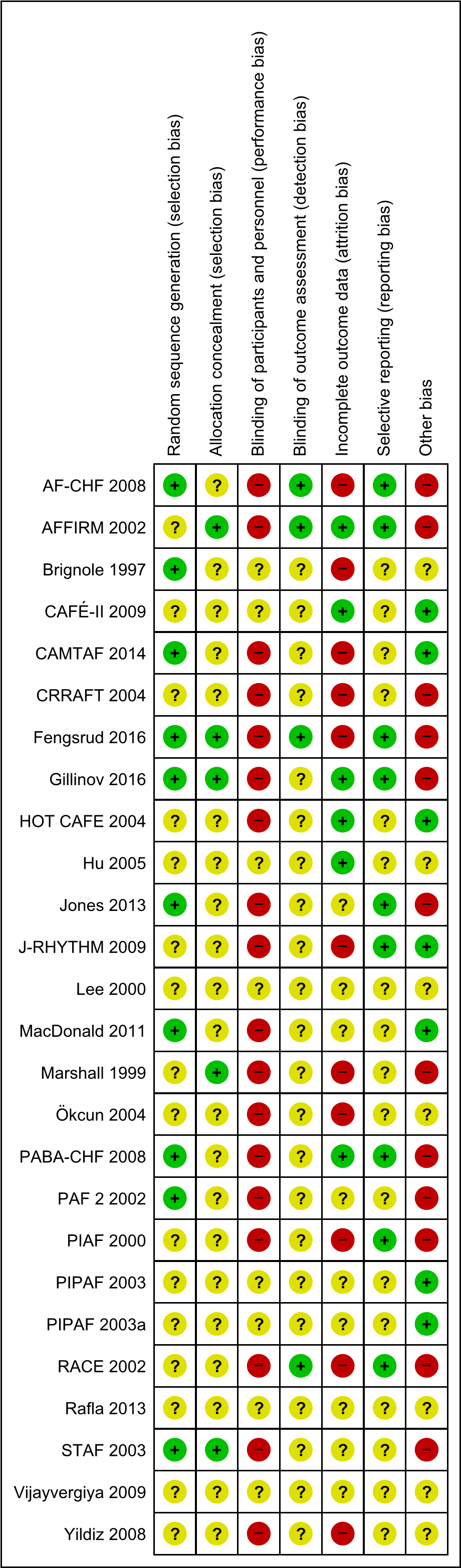

Supplement: S1 Table — (DOCX) [file pone.0186856.s037.docx]
