# Supplementary material for: The effects of rhythm control strategies versus rate control strategies for atrial fibrillation and atrial flutter: A systematic review with meta-analysis and Trial Sequential Analysis
Source: PLoS One. 2017 Oct 26;12(10):e0186856. doi: 10.1371/journal.pone.0186856 (PMC5658096; doi:10.1371/journal.pone.0186856)
Supplement: S2 Table — (DOCX) [file pone.0186856.s038.docx]

**S2 Table - inclusion- and exclusion criteria for each trial included**

|  | **Inclusion criteria** | **Exclusion criteria** |
| --- | --- | --- |
| **AF-CHF (2008)** | - LVEF = ≤35%. - History of congestive heart failure. - History of atrial fibrillation. - Eligibility for long-term therapy in either of the study groups. | - Persistent atrial fibrillation (>12 months). - Reversible cause of atrial fibrillation or heart failure. - Decompensated heart failure within 48 hours before randomisation. - Use of antiarrhythmic drugs for other arrhythmias. - 2^nd^ or 3^rd^ degree AV-block. - History of long-QT syndrome. - Previous AV-node ablation. - Anticipated cardiac transplantation within 6 months. - Renal failure (requiring dialysis). - Lack of birth-control in women of child-bearing potential. - Estimated life expectancy of less than 12 months. - Less than 18 years of age. |
| **AFFIRM (2002)** | - Atrial fibrillation was documented. - Age ≥ 65 years or other risk factors for stroke or death (i.e., systemic hypertension, diabetes mellitus, congestive heart failure, transient ischemic attack, prior stroke, left atrium >50 mm by echocardiogram, left ventricular fractional shortening <25% by echocardiogram, or LVEF < 40%), where atrial fibrillation was likely to be recurrent. - Atrial fibrillation was likely to cause illness or death. - Long-term treatment for atrial fibrillation, - Anticoagulant therapy was not contraindicated. - Eligible to undergo trials of at least two drugs in both treatment strategies. - Treatment with either strategy could be initiated immediately after randomization. | - Valvular heart disease. - Certain prior valve surgery or valvuloplasty. - Hypertrophic obstructive cardiomyopathy. - Reversible cause of atrial fibrillation. - Onset of qualifying atrial fibrillation within 7 days of CABG or myocardial infarction. - NYHA class IV (when optimally treated). - Heart transplant waiting list. - Other requirements for antiarrhythmic therapy. - Congenital long QT syndrome. - Lone atrial fibrillation. - WPW syndrome. - Implanted automatic cardioverter-defibrillator. - Prior maze or corridor procedure. - Prior AV-node ablation or modification. - Prior inability to cardiovert. - Amiodarone use, totaling more than 6 grams within the last 6 weeks. - Expected survival of 2 years or less. - Contraindication to warfarin. - Renal failure (requiring dialysis). - Women of childbearing potential. - Participation in another clinical trial. |
| **Brignole et al. (1997)** | - Age > 50 years. - Intolerable, recurrent paroxysmal atrial fibrillation of three or more episodes in the previous six months not controlled with three or more antiarrhythmic drugs. - Episodes that caused severe symptoms (e.g., palpitations, dyspnea, easy fatigue, and chest discomfort). - Duration of tachyarrhythmia episodes > 12 months. | - Symptomatic bradycardia (bradycardia-tachycardia form of sick sinus syndrome). - Need for a pacemaker implant for reasons other than tachyarrhythmia control. - Acute clinical diseases during the previous 6 months. |
| **CAFÉ-II (2009)** | - Age ≥ 18 years. - Persistent atrial fibrillation. - Chronic symptomatic heart failure (NYHA > class II) - Evidence of systolic dysfunction of echo. | - Contradicted for oral anticoagulants. |
| **CAMTAF (2014)** | - Age ≥ 18 years. - Persistent atrial fibrillation - Symptomatic heart failure (NYHA II-IV) - LVEF < 50%. - Adequate ventricular rate control (<80 bpm at rest and <110 bpm on moderate exertion). | - Reversible cause of heart failure. - Previous left atrial ablation. - Ant contraindication to catheter ablation. - Paroxysmal AF. - Symptoms that were clearly attributable to AF rather than HF (i.e., palpitations or dizziness) that might arguably mandate a rhythm control strategy. - Any event during the past 6 months that might continue to effect on LV function (including implantation of a pacemaker or cardiac resynchronization therapy device, cardiac surgery, myocardial infarction, or coronary revascularization), or a realistic expectation of these occurring within the next year. |
| **CRRAFT (2004)** | - Chronic rheumatic AF. | - AF of less than three month’s duration. - Left atrial size > 6.0 cm. - Valvular heart disease with hemodynamic compromise necessitating surgery. - Valvular surgery or ballon valvotomy past two months. - Pregnancy. - Contraindication to amiodarone. |
| **Fengsrud et al. (2016)** | - Age > 50 years - Symptomatic long-standing persistent atrial fibrillation. | - Left atrial thrombus formation. - Intolerance to warfarin. - FEV < 1.5 L/s. - Left anteroposterior diameter > 60 mm. - BMI > 35 kg/m^2^. - Previous thoracic surgery. |
| **Gillinov et al. (2016)** | - Hemodynamically stable adult participants. - Underwent heart surgery for CABG and/or valve repair or replacement (excluding mechanical valves), including re-operations. - Recent-onset postoperative atrial fibrillation persisting more than 60 minutes or recurrent episodes of atrial fibrillation during the index hospitalisation (<7 days after surgery). - No previous history of atrial fibrillation. | - History of atrial fibrillation. - LVAD insertion or heart transplantation. - Maze procedure. - TAVR. - History of or planned mechanical valve replacement. - Correction of complex congenital cardiac defect (excluding bicuspid aortic valve, atrial septal defect or PFO). - History of ablation for AF (including AFL). - Contraindications to amiodarone. - Contraindications to warfarin. - Received amiodarone within 6 weeks of index surgery. - Need for long-term anticoagulation. - Concurrent participation in an interventional trial. |
| **HOT CAFÉ (2004)** | - Age = 50 to 75 years - Atrial fibrillation present for at least 7 days, but not for more than 2 years - First clinically overt persistent episode of atrial fibrillation | - Documented inefficiency, intolerance to, or contraindications for treatment with antiarrhythmic drugs. - Presence of arrhythmia associated with an acute reversible condition. - Thyroid dysfunction. - Pregnancy or lactation. - History of myocardial infarction within 3 months preceding enrolment into the study. - Acute myocarditis. - Cardiac surgery during the previous 30 days. - Severe cardiac disability (i.e., New York Heart Association [NYHA] functional class IV). - Severe systemic hypertension not responding to treatment (diastolic pressure, 115 mm Hg); hypotension (systolic pressure, 90 mm Hg). - History of transient ischemic attack (with significantly marked vascular pathology requiring surgical intervention). - History of hemorrhagic stroke. - Ischemic stroke during the 3 months preceding entrance into the trial. - Any mitral stenosis or other valvular disease suitable for surgical treatment. - R-R intervals exceeding 3 s. - Ventricular response to AF of 90 beats/min (unrelated to drugs used to reduce ventricular rate). - Bundle branch block or QT-segment prolongation (i.e., corrected QT interval of 480 ms or uncorrected QT interval of 500 ms). - Alcoholism. - Contraindications to anticoagulation therapy. - Liver, kidney, or CNS damage. - Advanced chronic lung disease, malignancy, or any noncardiac illness associated with a life expectancy of 1 year. - Participation in another study. - Premenopausal women who were still capable of procreation and had not undergone tubal ligation or hysterectomy. |
| **Hu et al. (2005)** | - Atrial fibrillation after successful percutaneous mitral balloon valvotomy. - Duration of atrial fibrillation equal or less than 12 months. - Left atrial size ≤ 45 mm. | - Received an antiarrhythmic drug within five half-lives of the time of random assignment. |
| **J-RHYTHM (2009)** | - Paroxysmal atrial fibrillation (atrial fibrillation expected to convert spontaneously to sinus rhythm within 48h of onset). | - Initial AF episodes. - Contraindication for anticoagulation. - AF occurring during the acute phase of myocardial infarction or cardiac surgery. |
| **Jones et al. (2013)** | - Age = 18-80 years. - Symptomatic heart failure (NYHA II-IV). - LVEF ≤ 35% - Persistent atrial fibrillation (more than 7 days). | - Cardiovascular implantable electronic device insertion or cerebrovascular event within 6 months. - Coronary revascularization or atrioventricular nodal ablation within 3 months. - Reversible causes of AF or HF including thyroid dysfunction, alcohol, primary valvular disease, or recent major surgery. - Prior heart transplant or on urgent transplant waiting list. - Pregnancy. - Active malignancy. - Severe renal impairment. - Single chamber pacemaker and atrioventricular block. - Contraindications to general anesthesia or oral anticoagulation. |
| **Lee et al. (2000)** | - Age ≥ 18 years - No history of paroxysmal atrial fibrillation. - With atrial fibrillation after heart surgery lasting for at least 1 hour. | - Received antiarrhythmic therapy within 5 half-lives of the time of random assignment. - Neta blockers withdrawn after surgery. - Cardiogenic shock. - Creatinine level of >200 mikrog/mmol. - ASAT or ALAT concentrations 4 times the upper limit of normal. - Conduction disturbances before randomization. - Contraindication to anticoagulation. |
| **MacDonald et al. (2011)** | - Age = 18-80 years. - Advanced heart failure (NYHA II-IV) despite optimal heart failure treatment for at least 3 months. - LVEF < 35%. - Persistent atrial fibrillation, and no contraindication to cardiovascular MRI. | - Paroxysmal AF. - QRS duration >150 ms (or QRS 120-150 with evidence of mechanical cardiac dyssynchrony). - Contraindication to oral anticoagulant drugs. - Primary valvular disease or acute myocarditis as the cause of heart failure. - Coronary revascularisation within the preceding 6 months. - Pregnancy. - Expected cardiac transplantation within 6 months. |
| **Marshall et al. (1999)** | - Electrocardiographically documented PAF diagnosed at least 6 months previously. - AF was defined electrocardiographically as an absence of P waves, the presence of a fibrillating baseline, and an irregular ventricular rhythm. - Symptoms occurring at least monthly or intolerable drug side effects. - At least 2 different attempts at drug therapy (single drugs or drug combinations) to maintain sinus rhythm or control ventricular rate during AF. | - Coronary/valve disease requiring intervention. - Ventricular tachycardia documented on ECG. - Previous major thromboembolic event. - Coexisting medical condition limiting longevity to <1 year. |
| **PABA-CHF (2008)** | - Symptomatic, drug-resistant atrial fibrillation. - LVEF ≤ 40% or less - New York Heart Association class II or III heart failure. - Medication regimen of beta-blockers and angiotensin-converting-enzyme inhibitors and, in patients with NYHA class III heart failure, spironolactone. - Able to complete a 6-minute walk test. | - Reversible causes of atrial fibrillation and heart failure. - Postoperative atrial fibrillation. - Previous maze or maze-like surgery. - Previous left atrial ablation. - A life expectancy of 2 years or less. - A high probability of undergoing cardiac transplantation within the next 12 months. - A contraindication to antiarrhythmic or anticoagulation medications. - Severe pulmonary disease. - A documented intraatrial thrombus, tumor, or other abnormality that precludes catheter placement. - Cardiac surgery, myocardial infarction, or percutaneous coronary intervention within the previous 3 months. |
| **PAF 2 (2002)** | - Severely symptomatic paroxysmal atrial fibrillation. - Tachyarrhythmic episodes causing severe symptoms, including palpitations, dyspnoea, easy fatigue, and chest discomfort. - Failure of three or more antiarrythmic drugs (including amiodarone) to maintain stable sinus rhythm or to control symptoms. - Three or more episodes of paroxysmal tachyarrhythmia during the previous 12 months (an episode of paroxysmal tachyarrhythmia was defined as lasting more than 1 hour duration of tachyarrhythmic episodes >1 year. - Age >50 years. | - Previous implantation of a pacemaker for other reasons. - The need for a pacemaker implant for symptomatic bradycardia. - Intolerance to antiarrhythmic drugs. - Acute clinical diseases during the previous 6 months. - Associated severe general infections. - Geographical impossibility to follow-up. |
| **PIAF (2000)** | - Age = 18-75 years. - Symptomatic persistent atrial fibrillation of between 7 days and 360 days duration. | - Congestive heart failure, New York Heart Association (NYHA) class IV. - Unstable angina pectoris. - Acute myocardial infarction within 30 days. - Atrial fibrillation with an average rate of fewer than 50 beats per minute (BPM). - Known sick-sinus syndrome. - Atrial fibrillation in the setting of Wolff-Parkinson-White syndrome. - Coronary artery bypass graft surgery or valve replacement within the past 3 months. - Echocardiographic documentation of intracardiac thrombus formation. - Central or peripheral embolisation within the past 3 months. - Hypertrophic cardiomyopathy. - Amiodarone therapy within the past 6 months. - Acute thyroid dysfunction. - Pacemaker therapy. - Contraindications for systemic anticoagulation therapy. - Pregnancy. |
| **PIPAF (2003)** | - Haemodynamically stable patients with recent-onset atrial fibrillation (3-72 hours duration) after surgery | - Chronic AF prior to surgery. - Patients receiving IV pressor agents or patients located in the intensive care unit. - Patients with systolic blood pressure < 100mmHg. - Patients requiring immediate transthoracic direct current cardioversion to sinus rhythm because of clinical instability. - AF with a ventricular response of <60 beats per minute without rate controlling medications and without a functioning pacemaker (either permanent or temporary). - Patients with an underlying bundle branch block, history of second or third degree AV-block or known tachycardia/bradycardia syndrome without a functioning pacemaker. - Concurrent illness including untreated overt congestive heart failure, pneumonia, hyperthyroidism, hepatitis (enzymes elevated more than twice normal value), post-operative angina, and clinical evidence of digitalis toxicity. - Evidence of myocardial infarction within 7 days of randomization. - Patients with untreated hypokalemia (K<4.0 mEq/l) or hypomagnesemia (Mg<1.3 mEq/l). - A previous exposure to propafenone or ibutilide or contraindication to beta blockers. - Recent (within 5 half-lives) exposure to a type I or III anti-arrhythmic drug. - A corrected QT interval (Bazett’s correction) of greater than 440ms measured on the baseline surface ECG. - A history of Torsades de Pointes. - A history of embolism without adequate anticoagulation. - Failure of the patient or the attending physician to consent to the procedure. |
| **RACE (2002)** | - Persistent atrial fibrillation or flutter after a previous electrical cardioversion in whom oral anticoagulation was not contraindicated. | - Arrhythmia lasting longer than one year. - NYHA IV heart failure. - Current or previous treatment with amiodarone. - A pacemaker. - Patients in whom rate control was strictly indicated (at the discretion of the treating physician). - Patients in whom electrical cardioversion therapy was strictly indicated (also at the discretion of the treating physician). - Patients who had undergone more than one previous ECV during the last two years. - Patients with AF or AFL in the setting of acute disease (e.g. acute myocardial infarction). - Patients with sick sinus syndrome. - Patients with untreated severe mitral or aortic valve disease. - Patients with clinical hyperthyroidism (inclusion was permitted after three months of euthyroidism). - Patients with severe systemic disease. |
| **Rafla et al. (2013)** | - Persistent AF of more than one week and less than one year duration. - LVEF = <50%. | - Previous thromboembolism. - Left atrial (LA) or LA appendage thrombi. - LA > 60 mm - Intractable heart failure. |
| **STAF (2003)** | - Age > 18 years old. - Persistent atrial fibrillation - Atrial fibrillation for more than 4 weeks - Left atrial size >45mm. - Congestive heart failure, New York Heart Association (NYHA) functional class II or greater. - LVEF < 45%. - 1 or more prior cardioversion with arrhythmia recurrence. | - Permanent AF - A history of paroxysmal AF. - Left atrial size 70 mm. - LVEF < 20%. - WPW syndrome. - History of AV-node ablation or modification. - Absolute contraindications against oral anticoagulation. - Primarily success-less cardioversion within 4 weeks before randomization. - Pregnancy, malignancy, or other concomitant disease that would most likely limit the patient’s life expectancy to 3 years. |
| **Vijayvergiya et al. (2009)** | - Have had a percutaneous transluminal mitral commissurotomy. - Chronic atrial fibrillation | - Not stated. |
| **Yildiz et al. (2008)** | - Age > 18 years. - Hypertension (>140/90, or current use of antihypertensive drugs). - Persistent atrial fibrillation of more than 48 hours. | - Valvular heart diseases. - Coronary artery disease. - Heart failure. - Thyroid disease. - Renal failure. - Sick sinus syndrome. - Pulmonary embolism. - Acute pericarditis. - Diabetes mellitus, - Chronic obstructive lung disease - Hypertrophic obstructive cardiomyopathy - Atrial thrombus. |
| **Ôkcun et al. (2004)** | - Age > 18 years. - Persistent atrial fibrillation lasting more than 48 hours. - LVEF < 50%. | - Valvular heart diseases. - Coronary artery disease. - Thyroid disease. - Renal failure. - Sick sinus syndrome. - Pulmonary embolism. - Acute pericarditis. - Hypertrophic obstructive cardiomyopathy. - Atrial thrombus. |
