# Supplementary material for: The effects of rhythm control strategies versus rate control strategies for atrial fibrillation and atrial flutter: A systematic review with meta-analysis and Trial Sequential Analysis
Source: PLoS One. 2017 Oct 26;12(10):e0186856. doi: 10.1371/journal.pone.0186856 (PMC5658096; doi:10.1371/journal.pone.0186856)
Supplement: S3 Table — (DOCX) [file pone.0186856.s039.docx]

**S3 Table**

**Characteristics of included studies**

|  | **Study design** | **Type of atrial fibrillation** | **Rhythm control intervention** | **Rate control intervention** | **Rhythm control group (no. of participants)** | **Rate control group (no. of participants)** | **Maximum follow-up (months)** |
| --- | --- | --- | --- | --- | --- | --- | --- |
| **AF-CHF (2008)** | Parallel | Mixed | Antiarrhythmic therapy with or without electrical cardioversion | Mixed | 682 | 694 | 37 |
| **AFFIRM (2002)** | Parallel | Mixed | Antiarrhythmic therapy with or without electrical cardioversion | A beta-blocker, calcium blocker, digoxin, or a combination of these | 2033 | 2027 | 41 |
| **Brignole et al. (1997)** | Parallel | Paroxysmal | Mixed | AV-node ablation | 21 | 22 | 6 |
| **CAFÉ-II (2009)** | Parallel | Long-standing persistent | Amiodarone with or without electrical cardioversion | Digoxin and/or beta-blockers | 30 | 31 | 12 |
| **CAMTAF (2014)** | Parallel | Long-standing persistent | Catheter ablation | Mixed | 28 | 27 | 6 |
| **CRRAFT (2004)** | Parallel | Long-standing persistent | Amiodarone with or without electrical cardioversion | Diltiazem | 48 | 48 | 12 |
| **Fengsrud et al. (2016)** | Parallel | Long-standing persistent | Total endoscopic ablation | A beta-blocker, calcium blocker, digoxin, or a combination of these | 17 | 19 | 12 |
| **Gillinov et al. (2016)** | Parallel | Recent-onset | Amiodarone with or without electrical cardioversion | Mixed | 261 | 262 | 2 |
| **HOT CAFE (2004)** | Parallel | Persistent | Electrical cardioversion with antiarrhythmic drug therapy following sinus rhythm restoration | A beta-blocker, calcium blocker, digoxin, or a combination of these | 104 | 101 | 20.4 |
| **Hu et al. (2005)** | Parallel | Persistent | Amiodarone with or without electrical cardioversion | A beta-blocker, calcium blocker, digoxin, or a combination of these | 92 | 91 | 12 |
| **J-RHYHTM (2009)** | Parallel | Paroxysmal | Mixed | A beta-blocker, calcium blocker, digoxin, or a combination of these | 419 | 404 | 19.1 |
| **Jones et al. (2013)** | Parallel | Long-standing persistent | Catheter ablation | Digoxin and/or beta-blockers | 26 | 26 | 12 |
| **Lee et al. (2000)** | Parallel | Recent-onset | Mixed | A beta-blocker, calcium blocker, digoxin, or a combination of these | 27 | 23 | 2 |
| **MacDonald et al. (2011)** | Parallel | Long-standing persistent | Catheter ablation | Mixed | 22 | 19 | 8.3 |
| **Marshall et al. (1999)** | Parallel | Paroxysmal | Mixed | AV-node ablation | 21 | 39 | 4.15 |
| **PABA-CHF (2008)** | Parallel | Mixed | Catheter ablation | AV-node ablation | 41 | 40 | 6 |
| **PAF 2 (2002)** | Parallel | Paroxysmal | Mixed | AV-node ablation | 71 | 70 | 16 |
| **PIAF (2000)** | Parallel | Persistent | Amiodarone with or without electrical cardioversion | Diltiazem | 127 | 125 | 12 |
| **PIPAF I (2003)** | Parallel | Recent-onset | Propafenone | A beta-blocker, calcium blocker, digoxin, or a combination of these | 20 | 6 | (24 hours) |
| **PIPAF II (2003)** | Parallel | Recent-onset | Ibutilide | A beta-blocker, calcium blocker, digoxin, or a combination of these | 10 | 6 | (24 hours) |
| **RACE (2002)** | Parallel | Persistent | Electrical cardioversion with antiarrhythmic drug therapy following sinus rhythm restoration | A beta-blocker, calcium blocker, digoxin, or a combination of these | 266 | 256 | 27.6 |
| **Rafla et al. (2013)** | Parallel | Persistent | Not stated | Digoxin, carvedilol, and/or bisoprolol | 24 | 24 | 1 |
| **STAF (2003)** | Parallel | Long-standing persistent | Electrical cardioversion with antiarrhythmic drug therapy following sinus rhythm restoration | A beta-blocker, calcium blocker, digoxin, or a combination of these | 100 | 100 | 19.6 |
| **Vijayvergiya et al. (2009)** | Parallel | Chronic | Amiodarone | Mixed | 13 | 16 | 6 |
| **Yildiz et al. (2008)** | Crossover (data not included in any analysis) | Long-standing persistent | Electrical cardioversion with antiarrhythmic drug therapy following sinus rhythm restoration | A beta-blocker, calcium blocker, digoxin, or a combination of these | 169 | 52 | 39.5 |
| **Ökcun et al (2004)** | Crossover (data not included in any analysis) | Long-standing persistent | Electrical cardioversion with antiarrhythmic drug therapy following sinus rhythm restoration | A beta-blocker, calcium blocker, digoxin, or a combination of these | 80 | 74 | 36 |

**Characteristics of excluded studies**

|  | **Reason for exclusion** |
| --- | --- |
| **Beaver et al. (2016)** | The control group received both rate and rhythm control drugs. |
| **Kanorsky et al. (2006)** | Not randomized. |
| **Kirkutis et al. (2004)** | Both groups used rate control interventions. |
| **Petrac et al. (2005)** | The participants were in sinus rhythm when randomised. |
| **Schwartsman et al. (2015)** | The participants in the rhythm control group were in sinus rhythm when randomised. |
| **Ökcun et al. (2009)** | The participants in the rhythm control group were in sinus rhythm when randomised. |

**Characteristics of ongoing studies**

|  | **Expected finish date** | **Study design** | **Estimated number of participants** | **Type of atrial fibrillation** | **Rhythm control intervention** | **Rate control intervention** | **Maximum follow-up** |
| --- | --- | --- | --- | --- | --- | --- | --- |
| **ACWAS (2014) (NCT02248753)** | July 2018 | Parallel | 437 | Recent-onset | Flecainide and/or electrical cardioversion and amiodarone | Metoprolol, verapamil, and/or digoxin. | 12 months |
| **AFARC-LVF (2015) (NCT02509754)** | June 2017 | Parallel | 180 | Persistent | Catheter ablation | Medical rate control therapy. If necessary, AV-node ablation and CRT-D. | 12 months |
| **EAST (2013)** | November 2019 | Parallel | 3000 | Recent-onset | Either pulmonary vein isolation or antiarrhythmic drug therapy. | Rate control therapy. | 96 months |
| **Pak et al. I (2014) (NCT02285387)** | October 2019 | Parallel | 300 | No specific type stated. | Antiarrhythmic drugs and/or electrical cardioversion. If recurrence, catheter ablation | Beta blockers, calcium channel blockers, or/and digoxin | 12 months |
| **Pak et al. II (2014) (NCT02321085)** | November 2019 | Parallel | 150 | Persistent | Antiarrhythmic drugs and/or electrical cardioversion. If recurrence, catheter ablation | Beta blockers, calcium channel blockers, or/and digoxin | 12 months |
| **Pak et al. (2015) (NCT02633774)** | November 2017 | Parallel | 200 | Persistent | Antiarrhythmic drugs and/or electrical cardioversion. If recurrence, catheter ablation | Beta blockers, calcium channel blockers, or/and digoxin | 12 months |
| **Pilot-CRAfT (2014)** | March 2017 | Parallel | 60 | Long-standing persistent or permanent | Amiodarone and/or electrical cardioversion | Rate control drugs, and AV-nodal ablation if necessary. | 12 months |
| **RAFT-AF (2011)** | September 2019 | Parallel | 600 | Paroxysmal, persistent, and long-standing persistent | Catheter ablation with or without adjunctive antiarrhythmic drugs | Rate control therapy. | 60 months. |
