# Supplementary material for: The effects of rhythm control strategies versus rate control strategies for atrial fibrillation and atrial flutter: A systematic review with meta-analysis and Trial Sequential Analysis
Source: PLoS One. 2017 Oct 26;12(10):e0186856. doi: 10.1371/journal.pone.0186856 (PMC5658096; doi:10.1371/journal.pone.0186856)
Supplement: S4 Table — (DOCX) [file pone.0186856.s040.docx]

**S4 Table - Specific types of serious adverse events in each trial**

| **Trial** | **Year** | **RHYTHM:**  **Serious adverse events** | **Proportion of participants with a serious adverse event (rhythm)** | **RATE:**  **Serious adverse events** | **Proportion of participants with a serious adverse event (rate)** |
| --- | --- | --- | --- | --- | --- |
| **AF-CHF** | 2008 | 217 deaths from all causes; 20 strokes; 15 myocardial infarctions; 73 congestive heart failures; 14 cancers; 1 renal failure; 11 sepsis’; 70 cardiac procedures; 36 ventricular tachyarrhythmias; 58 bradyarrhythmias; 30 major non-CNS haemorrhages; 436 hospitalisations | 436 out of 647 | 228 deaths from all causes; 28 strokes; 9 myocardial infarctions; 57 congestive heart failures; 20 cancers; 2 renal failures; 25 sepsis’; 66 cardiac procedures; 37 ventricular tachyarrhythmias; 34 bradyarrhythmias; 25 major non-CNS haemorrhages; 409 hospitalisations | 409 out of 650 |
| **AFFIRM** | 2002 | 356 deaths from all causes; 12 torsades de pointes; 6 sustained ventricular tachycardia; 18 cardiac arrests; 106 central nervous system events; 5 disabling anoxic encephalopathies; 73 myocardial infarctions; 96 haemorrhage not involving the central nervous system; 7 systemic embolisms; 6 pulmonary embolisms; 42 congestive heart failures; 81 cancers; 1374 hospitalisations | 1374 out of 2033 | 310 deaths from all causes; 2 torsades de pointes; 9 sustained ventricular tachycardia; 11 cardiac arrests; 105 central nervous system events; 4 disabling anoxic encephalopathies; 67 myocardial infarctions; 107 haemorrhage not involving the central nervous system; 9 systemic embolisms; 2 pulmonary embolisms; 37 congestive heart failures; 52 cancers; 1220 hospitalisations | 1220 out of 2027 |
| **Brignole et al.** | 1997 | 1 heart failure | 1 out of 19 | None | 0 out of 21 |
| **CAFÉ-II** | 2009 | 1 death from all causes | 1 out of 30 | 1 death from all causes | 1 out of 31 |
| **CAMTAF** | 2014 | 1 stroke; 1 cardiac tamponade | 2 out of 26 | 1 death from all causes; 1 intracranial haemorrhage | 2 out of 24 |
| **CRRAFT** | 2004 | 1 thrombosis; 3 hospitalisations | 3 out of 45 | 5 deaths from all causes; 6 hospitalisations | 6 out of 40 |
| **Fengsrud et al.** | 2016 | 1 spinal bleeding | 1 out of 15 | None | 0 out of 19 |
| **Gillinov et al.** | 2016 | 2 deaths from all causes; 56 hospitalisations | 56 out of 261 | 3 deaths from all causes; 60 hospitalisations | 60 out of 262 |
| **HOT CAFÉ** | 2004 | 3 deaths from all causes; 3 ischemic strokes; 1 ventricular tachycardia; 2 implantations of pacemaker; 13 hospitalisations | 13 out of 104 | 1 death from all causes; 1 pulmonary embolism; 1 impotence; 3 implantations of pacemaker; 2 AV nodal ablations; 5 hospitalisations | 5 out of 101 |
| **Hu et al.** | 2005 | 11 hospitalisations | 11 out of 92 | 10 hospitalisations | 10 out of 91 |
| **J-RHYTHM** | 2009 | 4 deaths from all causes; 9 strokes; 1 systemic embolism; 2 major bleedings; 2 heart failures | 9 out of 419 | 3 deaths from all causes; 11 strokes; 1 systemic embolism; 1 major bleeding; 6 heart failures | 11 out of 404 |
| **Jones et al.** | 2013 | 1 death from all causes; 1 cardiac tamponade; 1 chest infection; 1 pulmonary oedema | 4 out of 26 | None | 0 out of 26 |
| **Lee et al.** | 2000 | 2 deaths from all causes; 1 acute pulmonary oedema; 1 myocardial infarction | 2 out of 27 | None | 0 out of 23 |
| **MacDonald et al.** | 2011 | 1 stroke; 2 cardiac tamponades; 1 worsening heart failure; 1 implantation of a pacemaker | 5 out of 22 | None | 0 out of 18 |
| **Marshall et al.** | 1999 | 1 thromboembolic event; 1 thyrotoxicosis | 2 out of 21 | 1 pneumothorax; 1 atrial lead displacement requiring repositioning | 2 out of 37 |
| **PABA-CHF** | 2008 | 1 pericardial effusion; 1 pulmonary oedema | 2 out of 41 | 2 left ventricular-lead dislodgemenst; 2 pocket hematomas; 1 pneumothorax | 5 out of 40 |
| **PAF 2** | 2002 | 4 deaths from all causes; 15 heart failures; 3 strokes; 1 myocardial infarction; 1 heart transplantation; 12 hospitalisations for heart failure | 15 out of 68 | 1 death from all causes; 7 heart failures; 1 stroke; 2 myocardial infarctions; 5 hospitalisations for heart failure | 7 out of 69 |
| **PIAF** | 2000 | 2 deaths from all causes; 1 ventricular fibrillation; 28 hospital admissions | 28 out of 122 | 2 deaths from all causes; 1 heart failure; 1 recurrent pulmonary embolism; 30 hospital admissions | 30 out of 124 |
| **PIPAF I** | 2003 | 1 heart failure | 1 out of 20 | None | 0 out of 6 |
| **PIPAF II** | 2003 | None | 0 out of 10 | None | 0 out of 6 |
| **RACE** | 2002 | 18 deaths from all causes; 12 heart failures; 21 thromboembolic complications; 9 bleedings; 12 severe adverse effects of antiarrhythmic drugs; 8 implantations of a pacemaker | 60 out of 266 | 18 death from all causes; 9 heart failures; 14 thromboembolic complications; 12 bleedings; 2 severe adverse effects of antiarrhythmic drugs; 3 implantations of a pacemaker | 44 out of 256 |
| **STAF** | 2003 | 4 deaths from all causes; 5 strokes | 9 out of 100 | 8 deaths from all causes; 1 stroke; 1 systemic embolism; 1 syncope | 10 out of 100 |
